# Julolidinyl aza-BODIPYs as NIR-II fluorophores for the bioimaging of nanocarriers

**DOI:** 10.1016/j.apsb.2024.04.002

**Published:** 2024-04-10

**Authors:** Chang Liu, Yifan Cai, Zichen Zhang, Yi Lu, Quangang Zhu, Haisheng He, Zhongjian Chen, Weili Zhao, Wei Wu

**Affiliations:** a Shanghai Skin Disease Hospital, Tongji University School of Medicine, Shanghai 200443, China; b Key Laboratory of Smart Drug Delivery of MOE, School of Pharmacy, Fudan University, Shanghai 201203, China; c Department of MediChinal Chemistry, School of Pharmacy, Fudan University, Shanghai 201203, China

**Keywords:** 3,5-Julolidinyl, Aza-BODIPY, Nanocarriers, Near-infrared II, Fluorescence imaging, Aggregation-caused quenching, Polymeric micelles, Polymeric nanoparticles

## Abstract

The aggregation-caused quenching (ACQ) rationale has been employed to improve the fluorescence imaging accuracy of nanocarriers by precluding free probe-derived interferences. However, its usefulness is undermined by limited penetration and low spatiotemporal resolution of NIR-I (700–900 nm) bioimaging owing to absorption and diffraction by biological tissues and tissue-derived autofluorescence. This study aimed to develop ACQ-based NIR-II (1000–1700 nm) probes to further improve the imaging resolution and accuracy. The strategy employed is to install highly planar and electron-rich julolidine into the 3,5-position of aza-BODIPY based on the larger substituent effects. The newly developed probes displayed remarkable photophysical properties, with intense absorption centered at approximately 850 nm and bright emission in the 950–1300 nm region. Compared with the NIR-I counterpart P2, the NIR-II probes demonstrated superior water sensitivity and quenching stability. ACQ1 and ACQ6 exhibited more promising ACQ effects with absolute fluorescence quenching at water fractions above 40% and higher quenching stability with less than 2.0% fluorescence reillumination in plasma after 24 h of incubation. Theoretical calculations verified that molecular planarity is more important than hydrophobicity for ACQ properties. Additionally, *in vivo* and *ex vivo* reillumination studies revealed less than 2.5% signal interference from prequenched ACQ1, in contrast to 15% for P2.

## Graphical abstract

Novel aggregation-caused quenching (ACQ) probes with bright NIR-II fluorescence were developed by incorporating highly planar and electron-rich julolidine at the 3,5-position of aza-BODIPY, improving the fluorescence imaging accuracy of nanocarriers.

**Figure:**
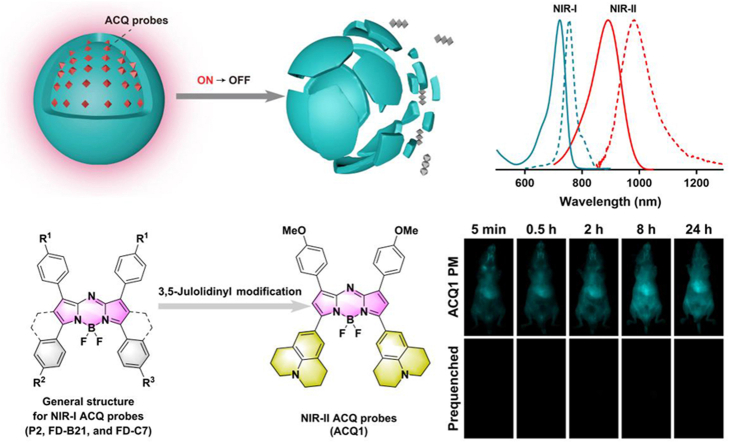

## 1 Introduction

**Figure 1:**
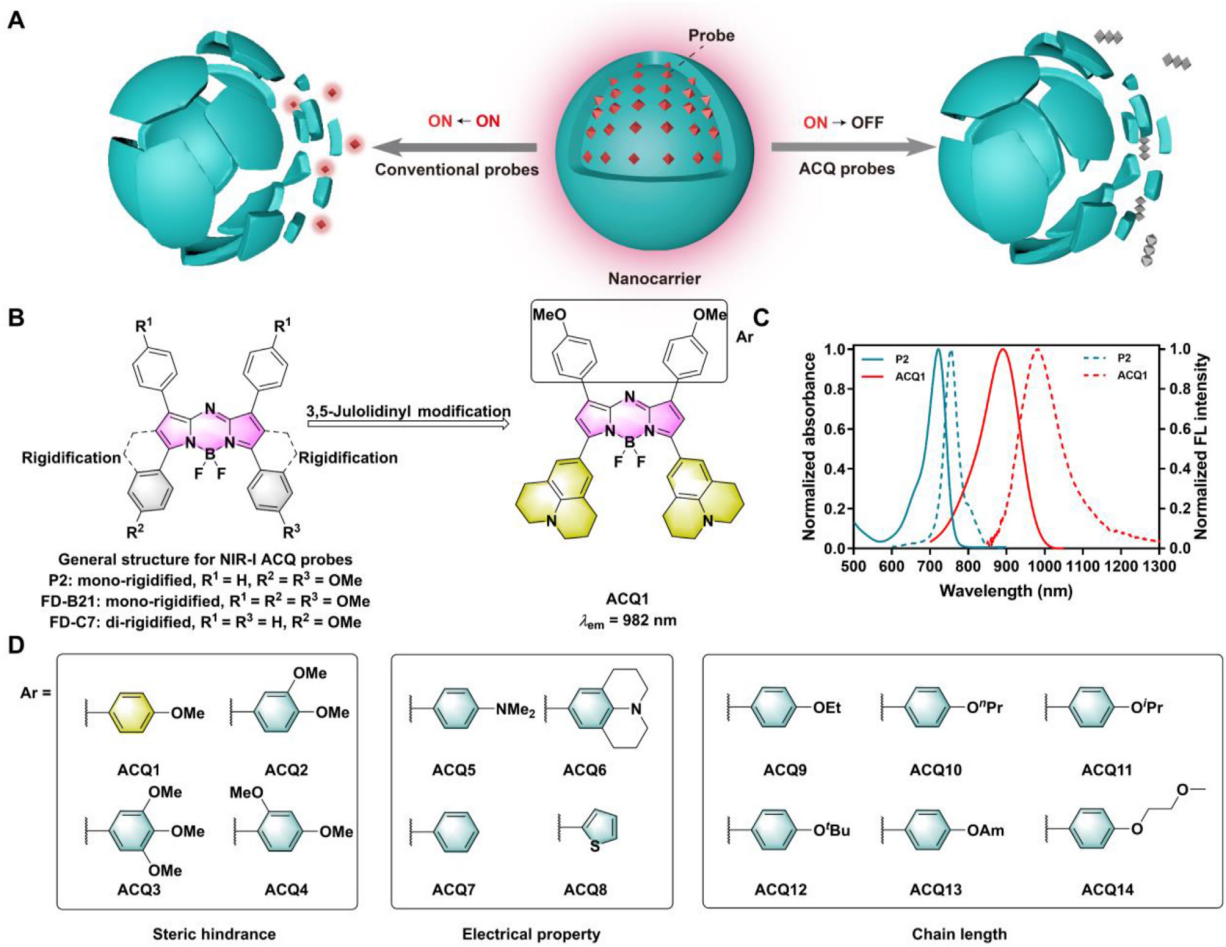
The design concept of 3,5-julolidinyl aza-BODIPYs as NIR-II ACQ probes. (A) Schematic illustration of the differences between conventional probes and ACQ probes for fluorescence tracking of nanocarriers. (B) Schematic illustration of installing julolidine into the 3,5-position of aza-BODIPYs with ACQ1 as an example. (C) Normalized absorption (solid line) and emission (dotted line) spectra of P2 and ACQ1. (D) Optimization of ACQ probes through fine-tuning planarity by changing the steric hindrance and electrical properties of the 1,7-substituents and hydrophobicity by varying the side chains.

Nanoparticle-based drug delivery systems (NDDSs) have shown promising potential for the treatment of cancer, infectious diseases, neurological disorders, etc^1^, ^2^, ^3^, ^4^. However, despite considerable research efforts, only a limited number of nanomedicine products have been successfully commercialized or approved for clinical applications^1^. Ignorance of the *in vivo* behaviors of nanocarriers is well acknowledged as a key factor leading to the current translational gap^5^^,^^6^. By unraveling the *in vivo* fate of nanocarriers, important information on biodistribution, bio-nano interactions, *in vivo* drug release, biodegradation, metabolism, and elimination can also be revealed, based on which more efficient optimization of NDDSs can be realized^6^, ^7^, ^8^, ^9^, ^10^, ^11^. Although various techniques such as computed tomography (CT), positron emission tomography (PET), single photon emission computed tomography (SPECT), and magnetic resonance imaging (MRI) have been utilized to track nanocarriers to study their *in vivo* fate, fluorescence imaging remains a valuable tool for real-time and noninvasive monitoring of nanocarriers *in vivo*^12^, ^13^, ^14^. However, traditional fluorescence imaging suffers from interference from free probes, which continue to emit fluorescence after release from nanocarriers (Fig. 1A)^15^. To distinguish free probe signals from nanocarrier signals, environmentally responsive fluorophores based on different rationales such as Förster resonance energy transfer (FRET), aggregation-induced emission (AIE), and aggregation-caused quenching (ACQ) have been developed^11^^,^^16^, ^17^, ^18^.

The ACQ effect, a phenomenon generally regarded as adverse in common fluorescence imaging, proves to be a viable tool for probing various types of nanocarriers due to its distinctive “on-to-off” features, which correspond well to the degradation dynamics of nanocarriers^11^^,^^19^^,^^20^. Several ACQ-based probes with an aza-dipyrromethene boron difluoride (aza-BODIPY) parent structure, encoded as P2, FD-B21, and FD-C7, have been developed in our laboratory to explore the *in vivo* bio-nano interactions of various nanocarriers such as lipid nanoparticles, nanocrystals, polymeric nanoparticles, and polymeric micelles^11^^,^^19^^,^^21^, ^22^, ^23^, ^24^, ^25^. Such probes emit bright fluorescence when encapsulated and well dispersed in the lipophilic cores of nanocarriers. Upon degradation or disassembly of the nanocarriers, the probes are released into the surrounding physiological environment, rapidly aggregate, and result in immediate and absolute fluorescence quenching, effectively eliminating interference from free probes and enabling accurate real-time tracking of nanocarriers *in vivo* (Fig. 1A)^11^. Unfortunately, their emission falls within the near infrared (NIR)-I region (700–900 nm). The sensitivity and accuracy of *in vivo* imaging are compromised by high tissue scatter and low signal penetration depth associated with NIR-I imaging^19^^,^^21^. Additionally, the nonnegligible reillumination of NIR-I ACQ probes also undermines imaging accuracy^11^^,^^19^^,^^21^. Recently, fluorescence imaging in the NIR-II region (1000–1700 nm) has emerged as a powerful tool at the frontiers of real-time diagnostics and biomarker detection due to reduced tissue absorption and scattering and ultralow autofluorescence^26^, ^27^, ^28^, ^29^, ^30^, ^31^, ^32^. Although not specifically designed for nanocarrier bioimaging, we were motivated to explore NIR-II probes with ACQ properties to address the aforementioned challenges in nanocarrier imaging.

Among various types of fluorescent probes, planar and easily aggregated aza-BODIPY, which has displayed high potential for the construction of NIR-I ACQ probes^19^^,^^21^^,^^23^, is a promising scaffold for the development of NIR-II ACQ probes. Although aza-BODIPY dyes have been widely used in fluorescence imaging, detection, and photodynamic therapy due to their excellent photophysical properties^33^, ^34^, ^35^, most of them are NIR-I dyes with emission wavelengths below 800 nm. To redshift their spectra, two main approaches, rotation restriction of aryl substituents and introduction of electron-donating moieties, have been adopted^35^. The rotation restriction of aryl substituents results in only a modest spectral redshift of approximately 70 nm^36^, ^37^, ^38^, whereas the introduction of highly electron-donating moieties, especially arylamines, results in a dramatically redshifted spectrum of approximately 200 nm with a tail falling in the NIR-II region through intramolecular charge transfer (ICT) from arylamine (donor) to electron-deficient aza-BODIPYs (acceptor), some of which have been utilized in NIR-II bioimaging of tumors and other disease states^35^^,^^39^, ^40^, ^41^, ^42^, ^43^. Julolidines, as *N*-heterocyclic aromatic compounds with restricted nitrogen availability and greater electron-donating ability, are favorable donor moieties for the construction of fluorescent dyes *via* ICT^44^^,^^45^. Moreover, their restricted structure and high planarity can increase the aggregation tendency and improve the ACQ properties. Although julolidine has been installed at the 1,7-position of aza-BODIPYs to obtain fluorophores with *λ*_em_ up to 1060 nm (*e.g.*, NJ1060)^40^, it has not been used for ACQ-based bioimaging. Moreover, density functional theory (DFT) calculations on typical tetraphenyl aza-BODIPY indicated that the 3,5-phenyl moiety contributes more strongly to molecular orbitals with increasing electron delocalization compared to their 1,7-counterparts (Supporting Information Fig. S1), suggesting greater substitution effects at the 3,5-position than at the 1,7-position, which was supported by bathochromic emission after changing the position of electron-rich substituents such as *p*-methoxyphenyl and *p*-(dimethylamino)phenyl from the 1,7-position to the 3,5-position^35^^,^^46^, ^47^, ^48^. Therefore, electron-rich and highly planar julolidine was installed into the 3,5-position of the aza-BODIPYs (Fig. 1B) to develop novel ACQ probes with bright NIR-II fluorescence. The resulting 3,5-julolidinyl ACQ1 with 1,7-(*p*-methoxyphenyl) displayed high brightness in the NIR-II region and outstanding ACQ properties with minimal reillumination after quenching both *in vitro* and *in vivo*.

## 2 Materials and methods

### 2.1 Materials

Methoxy polyethylene (mPEG)_2k_-poly (d,l-lactic acid) (PDLLA)_2k_ were obtained from Xiamen Sinopeg Biotech Co., Ltd. (Xiamen, China), while polycaprolactone (PCL, Mn = 45,000) and polyvinyl alcohol (PVA, Mw = 13,000–23,000, 87%–89% hydrolyzed) were acquired from Merck KGaA (Darmstadt, Germany). Others reagents and solvents were purchased from Bide Pharmatech (Shanghai, China). All reagents and solvents were used without further purification. Anhydrous solvents were acquired by standard methods before use.

### 2.2 Synthesis and characterization of NIR-II ACQ probes

Detailed preparation procedures and characterization results of all the NIR-II ACQ probes are described in the Supporting Information. High-resolution mass spectra were obtained on a mass spectrometer (AB Sciex Pte. Ltd., Triple TOF™ 5600+, Singapore). ^1^H nuclear magnetic resonance (NMR) and ^13^C NMR were recorded on Varian Model Mercury 600 MHz and 150 MHz spectrometers (Bruker Corp., Avance III™ HD 600 M, MA, US), respectively.

### 2.3 Photophysical properties

The spectra of 3,5-julolidinyl aza-BODIPYs were measured by a UV–Visible spectrophotometer (Lengguang Tech., 759S, Shanghai, China) and a spectrofluorometers (Edinburgh Instruments Ltd., FS5, Livingston, UK) for absorption and emission, respectively. The quantum yield of 3,5-julolidinyl aza-BODIPYs was determined with IR26 (*Φ* = 0.05%, 1,2-dichloroethane) as a reference and calculated according to Eq. (1):

(1) $$\Phi_{\left(\text{S}\right)}=\left(S_{\text{s}}/S_{\text{IR}26}\right)\cdot \left(n_{\text{S}}^{2}/n_{\text{IR}26}^{2}\right)\cdot \Phi_{\left(\text{IR}26\right)}$$

where *S* and *n* represent the slopes of the integrated NIR-II fluorescence intensity in the 850–1300 nm region against the absorbance at an excitation wavelength of 808 nm and the refractive index of the solvent, respectively. To improve the accuracy of the quantum yield, the slope was determined using at least five concentrations of 3,5-julolidinyl aza-BODIPYs with a large 0.02 absorbance gap at 808 nm in the 0–0.12 region^49^^,^^50^.

### 2.4 Water sensitivity

A solution of 3,5-julolidinyl aza-BODIPYs (5 mmol/L) in dimethyl sulfoxide (DMSO) was initially prepared and subsequently diluted to 25 μmol/L using DMSO/water binary systems with varying water fractions. The resulting decrease in fluorescence intensity was recorded as a function of the water fraction (Edinburgh Instruments Ltd.). After normalization, the water sensitivity of 3,5-julolidinyl aza-BODIPYs was determined.

### 2.5 Quenching stability

Probes were dissolved in DMSO (1.34 mmol/L) and diluted 100-fold with plasma or phosphate buffered saline (PBS) containing 1% Tween 80. After incubation at 37 °C and 120 rpm, the fluorescence intensity was recorded at different time points using NIRvana short-wave infrared (SWIR) camera (Princeton Instruments Inc., Trenton, NJ, US) with an external excitation of 808 nm laser, and the quenching stability was calculated by setting the fluorescence intensity of the same concentration of probe in DMSO (13.4 μmol/L) to 100%. To determine the quenching stability of the prequenched probes, the probe in DMSO (13.4 mmol/L) was diluted 100-fold with PBS to obtain a prequenched dispersion, which was immediately mixed with different media at a volume ratio of 1:10. The fluorescence recovery percentages at different time points after incubation were calculated by setting the fluorescence of the probe in DMSO (13.4 μmol/L) to 100%.

### 2.6 Preparation of probe-labeled nanocarriers

Probe-labeled polymeric micelles (PMs) were prepared by a thin-film dispersion method. The solutions of mPEG_2k_-PDLLA_2k_ (160 mg) and probe (0.535 μmol) in dichloromethane (DCM) (4 mL) were evaporated under vacuum (60 °C, 85 rpm) and redispersed in CH_3_CN to form a more homogeneous film. After removing the solvents by rotary evaporation, the obtained film was hydrolyzed by preheated PBS (4 mL, 60 °C) for 30 min. The solution was filtered through a 0.22 μm filter to obtain probe-labeled PMs. Probe-labeled polymeric nanoparticles (PNs) were prepared by an emulsification/solvent evaporation method. A solution of PCL (68 mg) and probe (0.268 μmol) in DCM (1 mL) was added dropwise to water (4 mL, 1% PVA, *v*/*v*), and the mixture was subjected to probe sonication (350 W, 3 min) (Ningbo Scientz Biotechnology Co., Ltd., SCIENTZ-IID, Ningbo, China) at 0 °C to prepare a primary emulsion. The primary emulsion was stirred at room temperature for 6 h to remove DCM and filtered through a 0.45 μm filter to obtain probe-labeled PNs.

### 2.7 Characterization of fluorescently labeled nanocarriers

The particle size, polymer dispersity index (PDI), and zeta potential of the probe-labeled nanocarriers were measured using a Malvern Zetasizer Nano ZSP (Malvern Instruments Ltd., Malvern, UK) after dilution to an appropriate concentration with deionized water. The morphology of the nanocarriers was detected *via* a JEM-1230 transmission electron microscope (NRI-MCDB Microscopy Facility, Santa Barbara, CA, US). The fluorescence stability of the nanocarriers was evaluated by the NIRvana SWIR camera (Princeton Instruments Inc.) with an external excitation of 808 nm laser. Fluorescently labeled nanocarriers were diluted 10-fold with either PBS or plasma and incubated at 37 °C and 120 rpm. Samples were taken at different time intervals to record changes in fluorescence intensity, and stability was calculated by setting the fluorescence at time zero to 100%. To measure the nanocarrier encapsulation efficiency (*EE*), 16 μmol/L probe DMSO stock solutions were prepared and diluted to different concentrations, and the fluorescence intensity was measured in triplicate using a spectrofluorometer (Edinburgh Instruments Ltd.) to obtain fluorescence intensity-probe concentration standard curves for different probes. Non-encapsulated free probes were removed by 0.22 μm membrane filtration. Probe-encapsulated nanocarrier dispersions were lyophilized to remove water and then redissolved in DMSO, and the fluorescence intensity of the probe (encapsulated part) in DMSO solution was measured. The concentration of encapsulated probes (*C*) was determined according to the standard curve, and the EE% of the nanocarriers was calculated using Eq. (2):

(2) $$\text{EE}\;\left(\%\right)=C/C_{0}\times 100$$

where *C*_0_ represents the theoretical concentration.

### 2.8 *In vivo* and *ex vivo* imaging

All experimental procedures were executed according to the protocols approved by Institutional Animal Care and Use Committee at the School of Pharmacy, Fudan University (Approval No.: 2022-08-YJ-WW-90). Male nude mice (Shanghai Slac Laboratory Animal Co., Ltd., Shanghai, China) weighing approximately 20 g were randomly divided into four groups, the ACQ1 group, ACQ3 group, ACQ6 group, and P2 group, with three mice in each group. Each group was administered the corresponding fluorescently labeled PMs (200 μL; probe concentration: 0.134 mmol/L) through tail vein injection. The biodistribution of the nanocarriers was monitored using an NIR-II imaging instrument (NIRvana SWIR camera from Princeton Instruments Inc. with an external excitation of 808 nm laser) at different time intervals under 808 nm excitation (43 mW/cm^2^) and imaged with a 1300 nm long-pass (LP) filter (exposure time, 500 ms) for the ACQ1 group, ACQ3 group, and ACQ6 group. To further explore the biodistribution of the nanocarriers *ex vivo*, the mice were sacrificed after 24 h and subjected to cardiac perfusion with 0.9% normal saline. Dissected organs and tissues were imaged with the same parameters except for a reduced exposure time of 300 ms. The *in vivo* and *ex vivo* biodistribution of P2-labeled nanocarriers was monitored using an *in vivo* imaging system (IVIS, 124262, PerkinElmer Inc., MA, US) with excitation at 710 nm and emission at 750–770 nm. Moreover, the *in vivo* and *ex vivo* reillumination of the prequenched probes was also investigated under the corresponding conditions after injection of the same amount of prequenched dispersions. The biodistribution of the nanocarriers and the reillumination of the prequenched probes were quantified using the mean gray value of the corresponding region manipulated by ImageJ.

## 3 Results and discussion

### 3.1 Design and synthesis

Incorporation of highly planar and electron-rich julolidine at the 3,5-position of aza-BODIPY yielded 3,5-julolidinyl ACQ1 with 1,7-(*p*-methoxyphenyl), which exhibited dramatic bathochromic spectra with emission falling in the NIR-II region (Fig. 1C). Encouraged by the bright fluorescence in the NIR-II region, NIR-II ACQ probes were developed by optimizing ACQ1 with fixed 3,5-julolidinyl moieties. Constraining the conformation of ACQ1 by fusion of the 2,6-position and 3,5-julolidinyl, which is believed to be favorable for NIR-II emission and ACQ properties^19^^,^^21^^,^^23^, failed due to preparative difficulties. To avoid harmful steric hindrance and complex preparative procedures^35^, hydrogens were installed at the 2,6-position. Molecular planarity plays a significant role in facilitating the degree of *π*–*π* stacking that determines ACQ properties^11^^,^^21^^,^^51^. Changing the steric hindrance of substituents is a well-known strategy for fine-tuning molecular planarity^52^, ^53^, ^54^. Moreover, changing the electronic properties of substituents can fine-tune the conjugation and dihedral angle between peripheral rings and the aza-BODIPY core and influence molecular planarity. Therefore, analogs of ACQ1 containing various 1,7-substituents with different steric hindrance (ACQ2‒4) and electrical properties (ACQ5‒8) were investigated to fine-tune the molecular planarity (Fig. 1D). Recognized as the driving force for *π*–*π* stacking^11^^,^^51^, the hydrophobicity of the ACQ probes was also optimized by installing hydrophobic (ACQ9‒13) and hydrophilic (ACQ14) side chains on the 1,7-substituents of ACQ1 (Fig. 1D).

**Scheme 1:**
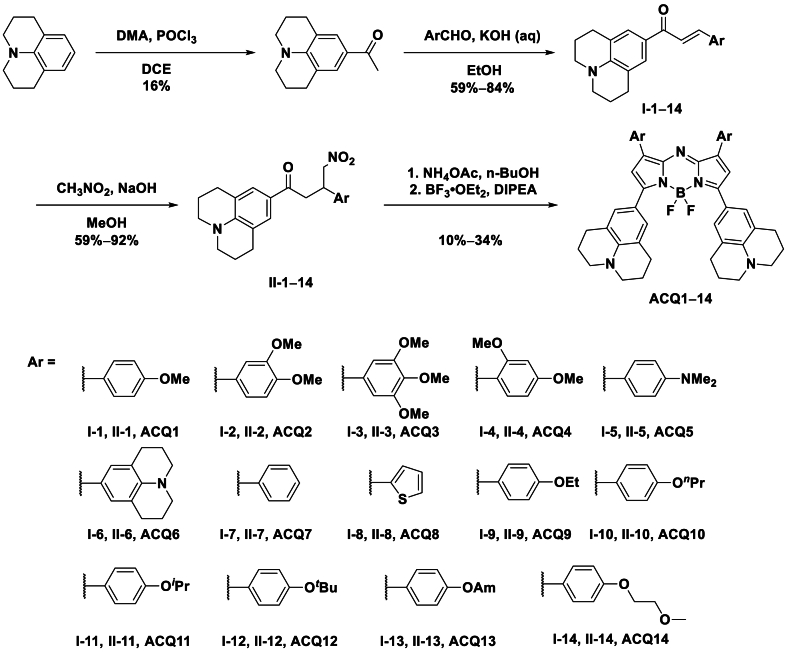
Synthetical routes of 3,5-julolidinyl aza-BODIPYs.

These 3,5-julolidinyl aza-BODIPYs were prepared following previous procedures with modifications^33^^,^^46^. As shown in Scheme 1, 9-acetyljulolidine was first prepared from julolidine through a modified Vilsmeier–Haack reaction. After condensation with aromatic aldehydes under basic conditions, chalcone derivatives (I1‒14) were obtained. Heating with nitromethane and sodium hydroxide in methanol afforded Michael additional products of nitromethane to chalcones (II1‒14). Upon heating with an ammonia source (ammonium acetate) in *n*-butanol and further chelation with boron trifluoride etherate, 3,5-julolidinyl aza-BODIPYs (ACQ1‒14) were synthesized. Notably, some ^1^H NMR signals of propylene in the julolidinyl moiety were missing in common deuterated alkyl halide solvents (CDCl_3_ and CD_2_Cl_2_), which was attributed to their high aggregation tendency and slow relaxation inactivation process. Fortunately, when the deuterated solvents were changed to DMSO-*d*_6_ or pyridine-*d*_5_, the signal peaks of 3,5-julolidinyl aza-BODIPYs appeared completely in the NMR spectra. Therefore, these deuterated solvents were utilized in the NMR experiments. All the prepared aza-BODIPYs were characterized by NMR and high-resolution mass spectrometry (HRMS), except for ACQ9, whose NMR spectrum was inaccessible due to its low solubility in DMSO-*d*_6_ and pyridine-*d*_5_.

### 3.2 Photophysical properties

**Figure 2:**
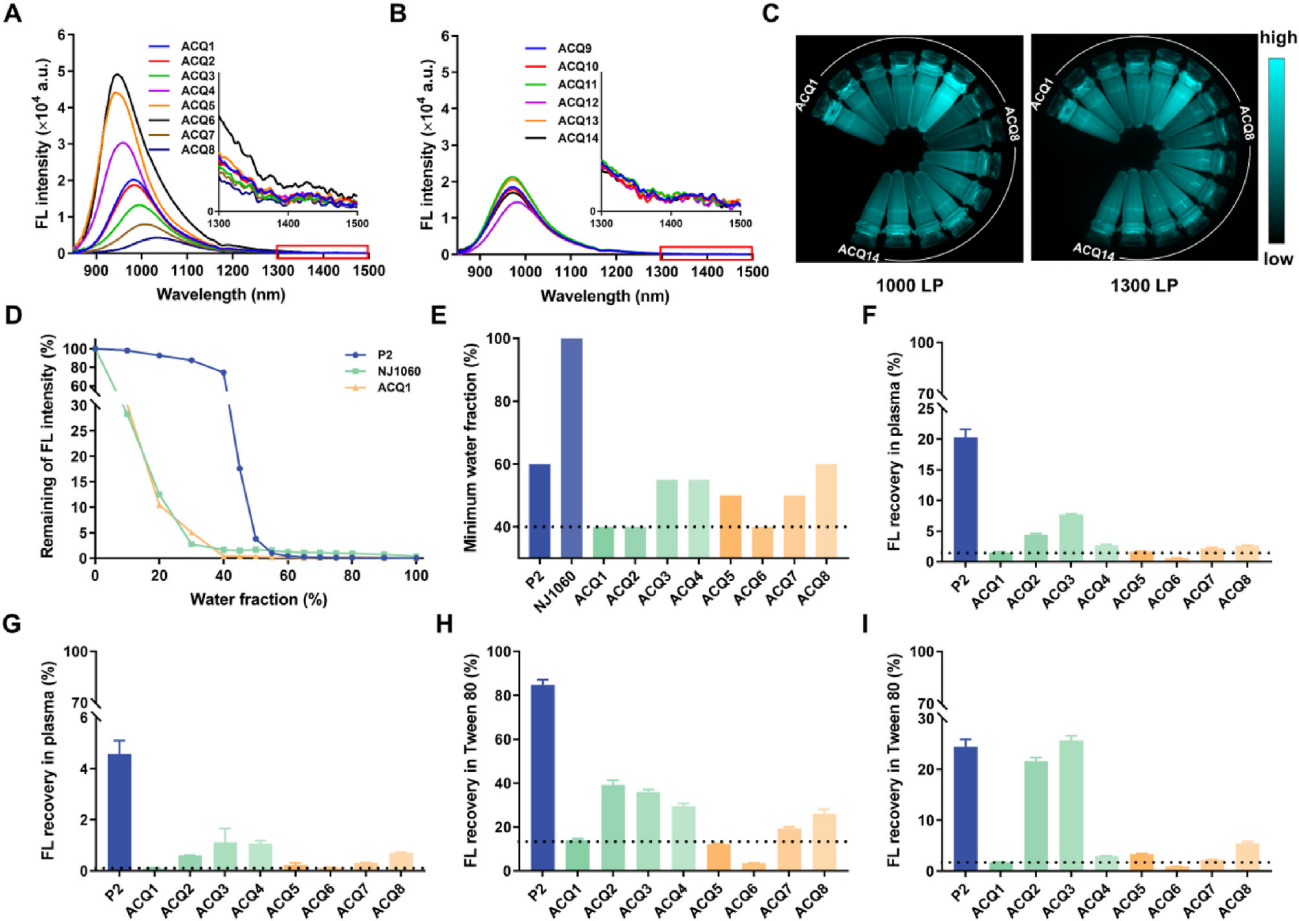
Spectrographic and ACQ properties of 3,5-julolidinyl aza-BODIPYs. (A,B) The emission spectra of ACQ1‒8 (A) and ACQ9‒14 (B) in DMSO. Inset: zoom-in emission spectra in the range of 1300–1500 nm. (C) Fluorescence image of ACQ1‒14 (5 μmol/L) in DMSO using 1000 and 1300 nm LP filters (*λ*_ex_ = 808 nm). (D) Water quenching fluorescence curves of P2, NJ1060 and ACQ1 in DMSO/water binary systems. (E) Water sensitivity fraction in DMSO/water binary systems after complete quenching of fluorescence. Fluorescence reillumination (%) of (F) dissolved and (G) prequenched probes in plasma after 24 h of incubation. (*n* = 3; mean ± SD). Fluorescence reillumination (%) of (H) dissolved and (I) prequenched probes in 1% Tween 80 after 24 h incubation. (*n* = 3; mean ± SD). Each dotted line in (E)‒(I) represents the level of ACQ1.

The spectrographic properties of 3,5-julolidinyl aza-BODIPYs in several solvents (PhMe, CHCl_3_, THF, CH_3_CN, and DMSO) with different polarities were then investigated utilizing NJ1060 as a reference (Table 1 and Supporting Information Table S1). Installing julolidine confers the aza-BODIPYs not only high light-harvesting ability (*ε* = 60,000–83,000 L/(mol·cm)) but also dramatically redshifted spectra with absorption centered at approximately 850 nm and emission within the 950–1300 nm region (Fig. 2A and B, Supporting Information Figs. S3 and S4) in comparison with P2 (Supporting Information Fig. S5), consistent with the decreasing energy gap between the highest occupied molecular orbital (HOMO) and the lowest unoccupied molecular orbital (LUMO) (Supporting Information Fig. S6). Although the fluorescence quantum yield decreased with bathochromic emission^32^, these probes displayed bright fluorescence with a quantum yield of 1.50%–5.23% in the region of 850–1500 nm and 0.38%–0.47% in the region of 1000–1500 nm. When the solvent polarity increased from nonpolar PhMe to polar DMSO, typical characteristics of the ICT effect such as a bathochromic spectrum (from 824–876 to 873–932 nm for absorption, from 866–922 to 941–1028 nm for emission), increasing Stokes shift (from 32‒46 nm to 52–96 nm), and a drastic reduction in fluorescence intensity, were observed (Table 1, Table S1, Figs. S3 and S4). The charge separation and ICT effect were also confirmed by the reduced delocalization of the LUMO in 3,5-julolidinyl, especially in nitrogen, in comparison with that of the HOMO (Fig. S6). Compared to 1,7-julolidinyl NJ1060, ACQ1 with vertically flipped peripheral substituents displayed enhanced light absorption capability at approximately 850 nm with an almost twofold increase in molar coefficient and brighter fluorescence in the 950–1300 nm region after excitation with an 808 nm laser, illustrating the superior potential of 3,5-julolidinyl aza-BODIPYs for bioimaging in the NIR-II region (Supporting Information Fig. S7). The introduction of substituents with different steric hindrance and electrical properties at the 1,7-position resulted in a dramatic change in the spectroscopic properties (ACQ2‒8 *vs.* ACQ1). Increasing the number of methoxy groups at the less sterically hindered *meta-*position had little effect on their spectroscopic properties, with only slight absorption/emission redshifts and a decrease in quantum yield (ACQ1 *vs.* ACQ2 *vs.* ACQ3), while installing a methoxy moiety at a more hindered *ortho*-position resulted in hypsochromic absorption/emission by 12–25 nm (ACQ4 *vs.* ACQ1) due to decreasing coplanarity and the weakening contribution of the 1,7-substituents. Additionally, this modification resulted in restricted substituent rotation, leading to a slight increase in fluorescence quantum yield (from 3.44% to 3.68%). Contrary to previous findings that electron-rich ring modification at the 1,7-position resulted in spectral redshift through the ICT effect from the electron-rich rings at the 1,7-position to the aza-BODIPY core in 3,5-(*p*-methoxyphenyl) aza-BODIPYs^35^^,^^40^^,^^41^^,^^46^^,^^47^, increasing the electron-donating ability of 1,7-substituents (ACQ5 and ACQ6) in 3,5-julolidinyl aza-BODIPY resulted in a decrease in the ICT effect with hypsochromic absorption/emission (*λ*_ab_ of 826–889 nm, *λ*_em_ of 866–946 nm, and 1–41 nm shorter than ACQ1), a decrease in the Stokes shift (4–40 nm shorter than ACQ1) and brighter emission (*Φ* of 4.68%–5.23%, *ε* × *Φ* of 3884–4079). Considering the electronic polarization and charge separation from the electron-rich 1,7-julolidinyl to the electron-deficient aza-BODIPY core (Fig. S6), this phenomenon was attributed to the electron-donating competition of 1,7-julolidinyl and the reduced electron-donating and charge separation of 3,5-julolidinyl. This assumption was also supported by the enhanced ICT effect with broad and less intense absorption (*λ*_ab_ = 852–911 nm, *ε* = 60,000 L/(mol·cm)) and long and faint emission (*λ*_em_ = 897–1003 nm, *Φ* = 2.45%) after changing the 1,7-substituents with low electron-donating phenyl groups (ACQ7 *vs.* ACQ1). Installing thiophen-2-yl at the 1,7-position resulted in the probe with the longest spectrum (*λ*_ab_/*λ*_em_ = 932/1028 nm in DMSO), which was ascribed to the low sterically hindered thiophene resulting in a decreasing twist angle and increasing conjugation, albeit with a slight decrease in the quantum yield (*Φ* = 1.50%). Notably, its brightness was still almost twice that of NJ1060, illustrating the superior photophysical properties of 3,5-julolidinyl aza-BODIPYs. As expected, alkyl side chains had little effect on absorption and emission, and analogs of ACQ1 with various chains (ACQ9‒14) retained the outstanding photophysical properties of ACQ1 such as intense absorption centered at approximately 834–896 nm (*ε* = 60,000–68,000 L/(mol·cm)) and bright emission centered at approximately 880–991 nm (*Φ* = 3.20%–3.89%). Meanwhile, fluorescence imaging indicated that there were not only obvious fluorescence signals in the NIR-II region (1000 LP filter) but also some fluorescence through the 1300 LP filter from the emission tail, which was the superior region for NIR-II imaging (Fig. 2C). Among them, ACQ6 displayed the brightest fluorescence under these two filters, while the fluorescence intensities of ACQ7 and ACQ8 were slightly weak. Overall, 3,5-julolidinyl aza-BODIPYs exhibited potential application in NIR-II imaging with intense absorption at approximately 850 nm and bright fluorescence in the NIR-II region.

**Table 1:** Spectrographic properties of 3,5-julolidinyl aza-BODIPYs.

Compd.	Ar	*λ*_ab_ (nm)^a^	*λ*_em_ (nm)^a^	Stokes shift (nm)^a^	*ε* (L/(mol·cm))^b^	*Φ*^c^ (850–1500 nm)	ε × *Φ*^b^	*Φ*^c^ (1000–1500 nm)
ACQ1	*p*-Methoxyphenyl	890	982	92	71000	3.44%	2442	0.43%
ACQ2	*m*,*p*-Dimethoxyphenyl	891	984	93	68000	3.09%	2101	0.43%
ACQ3	*m*,*m*,*p*-Trimethoxyphenyl	904	993	89	70000	2.34%	1638	0.41%
ACQ4	*o*,*p*-Dimethoxyphenyl	873	959	86	66000	3.68%	2429	0.38%
ACQ5	*p*-(Dimethylamino)phenyl	878	946	68	78000	5.23%	4079	0.45%
ACQ6	Julolidinyl	889	941	52	83000	4.68%	3884	0.42%
ACQ7	Phenyl	911	1003	92	60000	2.45%	1470	0.43%
ACQ8	Thiophen-2-yl	932	1028	96	62000	1.50%	930	0.40%
ACQ9	*p*-Ethoxyphenyl	889	977	88	60000	3.75%	2250	0.45%
ACQ10	*p*-Propoxyphenyl	889	979	90	60000	3.82%	2292	0.46%
ACQ11	*p*-Isopropoxyphenyl	889	977	88	64000	3.89%	2490	0.46%
ACQ12	*p*-(*tert*-Butoxy)phenyl	896	991	95	62000	3.20%	1984	0.45%
ACQ13	*p*-(Pentyloxy)phenyl	889	979	90	68000	3.73%	2536	0.44%
ACQ14	*p*-(2-Methoxyethoxy)phenyl	891	979	88	60000	3.73%	2238	0.47%
NJ1060	‒d	893	1054	161	37000	1.33%	492	0.35%

a In DMSO.

b In CHCl_3_.

c Measured in CHCl_3_ using IR26 (*Φ* = 0.05%, 1,2-dichloromethane) as a reference^55^.

d The chemical structure of NJ1060 is displayed in Fig. S2. ‒, not applicable.

### 3.3 Quenching ability and reillumination of the NIR-II probes

To ensure minimal interference from nonencapsulated or free probes, ACQ probes should quickly form aggregates and display immediate and absolute fluorescence quenching when released from nanocarriers and dispersed in a polar physiological environment^11^. Our previous investigations on aza-BODIPY-based NIR-I fluorophores demonstrated a typical ACQ effect in response to environmental changes to an aqueous medium with clear “on-to-off” fluorescence switching in contrast to conventional probes that do not show a typical ACQ effect^19^^,^^21^^,^^23^. Encouraged by the outstanding photophysical properties of 3,5-julolidinyl aza-BODIPYs containing 1,7-substituents with different steric hindrance and electrical properties, their water sensitivity was investigated in DMSO/water binary systems in comparison with that of the NIR-I ACQ probe P2 and NJ1060. Although different water-miscible solvents have been tested, only DMSO demonstrates good solubility for all fluorophores. Due to their high hydrophobicity, these probes displayed typical ACQ phenomena with decreasing fluorescence intensity in response to water fraction increase, though at different rates (Fig. 2D and Supporting Information Fig. S8). The fluorescence of NJ1060 and the NIR-II ACQ series decreased rapidly in response to the increase in the water fraction from 0% to 40%, in contrast to the slight decrease in fluorescence for P2. Among them, ACQ1 and NJ1060 demonstrated the rapidest quenching, with a dramatic decrease in fluorescence at a 10%–20% water fraction and less than 2% fluorescence remaining at a 40% water fraction. However, NJ1060 still displayed some fluorescence until the water fraction reached 100%, rendering it unsuitable as an ACQ probe. To our delight, the fluorescence of ACQ1 was completely quenched at less than 40% water fraction, implying that the incorporation of 3,5-julolidinyl into the aza-BODIPY structure confers superior water sensitivity, probably owing to reinforced hydrophobicity and planarity. Using the minimum water fraction required for complete fluorescence quenching as a measure, termed as water sensitivity fraction (WSF) thereafter^21^^,^^23^, the water sensitivity of all the ACQ probes was compared with that of NJ1060 and P2. A smaller WSF is always associated with greater water sensitivity or a greater ACQ effect^21^^,^^23^. As displayed in Fig. 2E, P2 and NJ1060 showed absolute quenching with WSFs of up to 60% and 100%, respectively, while most 3,5-julolidinyl aza-BODIPYs demonstrated a superb WSF at 40%–55%. ACQ1 with *p*-methoxyphenyl at the 1,7-position exhibited superior water sensitivity with 40% WSF. Although the addition of another methoxy group at the *meta*-position of the phenyl, where there is relatively little steric hindrance, had little effect on water sensitivity (ACQ2 *vs.* ACQ1), further increasing the number of methoxy moieties (ACQ3) or changing the position of the methoxy group to a more sterically hindered *ortho*-position (ACQ4) led to an increase in the WSF to 55%, which could be attributed to the increase in steric hindrance and consequent reduction in molecular planarity. Although 1,7-substitution with a *p*-(dimethylamino)phenyl moiety (ACQ5) resulted in a decrease in WSF (55%), a further increase in planarity by rigidification (ACQ6) led to a decrease in WSF to 40%, which was comparable to that of ACQ1. Substitution by phenyl or thienyl groups led to an increase in WSF (ACQ7&8 *vs.* ACQ1).

Aggregated and quenched probes can repartition into hydrophobic domains of biomacromolecules and lipid constructs and can be reilluminated to cause artifacts^11^. An ideal ACQ probe should possess superior quenching stability and minimal fluorescence reillumination^19^^,^^21^. Therefore, the quenching stability of 3,5-julolidinyl aza-BODIPYs and water-prequenched probes in plasma was assessed by monitoring their fluorescence recovery in plasma at different incubation times with P2 as a reference (Supporting Information Fig. S9A‒S9D). Regardless of whether the probes were prequenched, time-dependent fluorescence recovery was observed. Although P2 was immediately and absolutely quenched at the initial stage, there was still up to 20% and 5% reillumination after 24 h incubation for the dissolved and prequenched probes, respectively, which probably undermined the accuracy of nanocarrier tracking. However, despite fluorescence reillumination trend similar to that of P2, most of the 3,5-julolidinyl aza-BODIPYs showed promising quenching stability in plasma, with an over 5-fold decrease in fluorescence reillumination. Fluorescence reillumination at 24 h after incubation is displayed in Fig. 2F and G. In contrast to the high fluorescence reillumination of P2, when ACQ1 was incubated in plasma for 24 h, only 1.5% and 0.1% of the fluorescence was recovered for the dissolved and prequenched probes, respectively, illustrating outstanding quenching stability. Notably, the fluorescence reillumination of 3,5-julolidinyl aza-BODIPYs exhibited a 1,7-substituent effect similar to water sensitivity. Reducing the planarity by increasing the steric hindrance at the 1,7-position resulted in greater fluorescence recovery (ACQ2‒4 *vs.* ACQ1). Although ACQ2 had the same WSF as ACQ1 (Fig. 2E), its fluorescence reillumination increased to 4% and 0.6% for the dissolved and prequenched probes, respectively. ACQ5, with a *p*-(dimethylamino)phenyl group, displayed quenching stability comparable to that of ACQ1. Interestingly, the rigidification of nitrogen by two propylene molecules resulted in the most quenching-stable ACQ6 with ultralow fluorescence reillumination (0.5% for the dissolved probe and 0.1% for the prequenched probe). However, changing the 1,7-substituent to phenyl (ACQ7) or thiophen-2-yl (ACQ8) resulted in a decrease in quenching stability with a slight increase in fluorescence recovery to 2.1%–2.6% and 0.3%–0.7% for the dissolved and prequenched probes, respectively.

To confirm the effectiveness of 3,5-julolidinyl aza-BODIPYs as novel ACQ probes, their quenching stability in PBS containing 1% Tween 80, a surfactant with strong fluorophore extraction capacity^19^^,^^21^^,^^23^, was also explored (Supporting Information Fig. S10, Fig. 2H and I). Similarly, there was an increase in fluorescence recovery with time. The fluorescence recovery of P2 in the dissolved and prequenched states increased dramatically to 80% and 25%, respectively, after 24 h of incubation. In contrast, most of the 3,5-julolidinyl aza-BODIPYs displayed better quenching stability than did P2, with less than 30% and 5% fluorescence reillumination for both the dissolved and prequenched probes, respectively. The fluorescence reillumination of ACQ1 and ACQ6 was the lowest, with less than 15% and 2% fluorescence reillumination for the dissolved and prequenched probes, respectively, demonstrating robust quenching stability when challenged by strong solubilizers.

For optimization based on hydrophobicity, alkylation in 1,7-substituents (ACQ9‒13) increased the quenching stability in 1% Tween 80 (Supporting Information Fig. S11). However, the water sensitivity and fluorescence recovery in plasma, especially for prequenched probes, were only slightly improved, indicating that increasing hydrophobicity has little benefit for ACQ probe development. The decrease in the ACQ properties of ACQ14 containing 2-methoxyethoxy groups was ascribed to the unfavorable molecular planarity and aggregation tendency. Overall, 3,5-julolidinyl aza-BODIPYs displayed superior ACQ properties compared to P2. In summary, among all the probes tested, ACQ1 and ACQ6 demonstrated the most promising water sensitivity and quenching stability, and molecular planarity was more important for ACQ properties than hydrophobicity.

### 3.4 Geometric structures of 3,5-julolidinyl aza-BODIPYs

**Figure 3:**
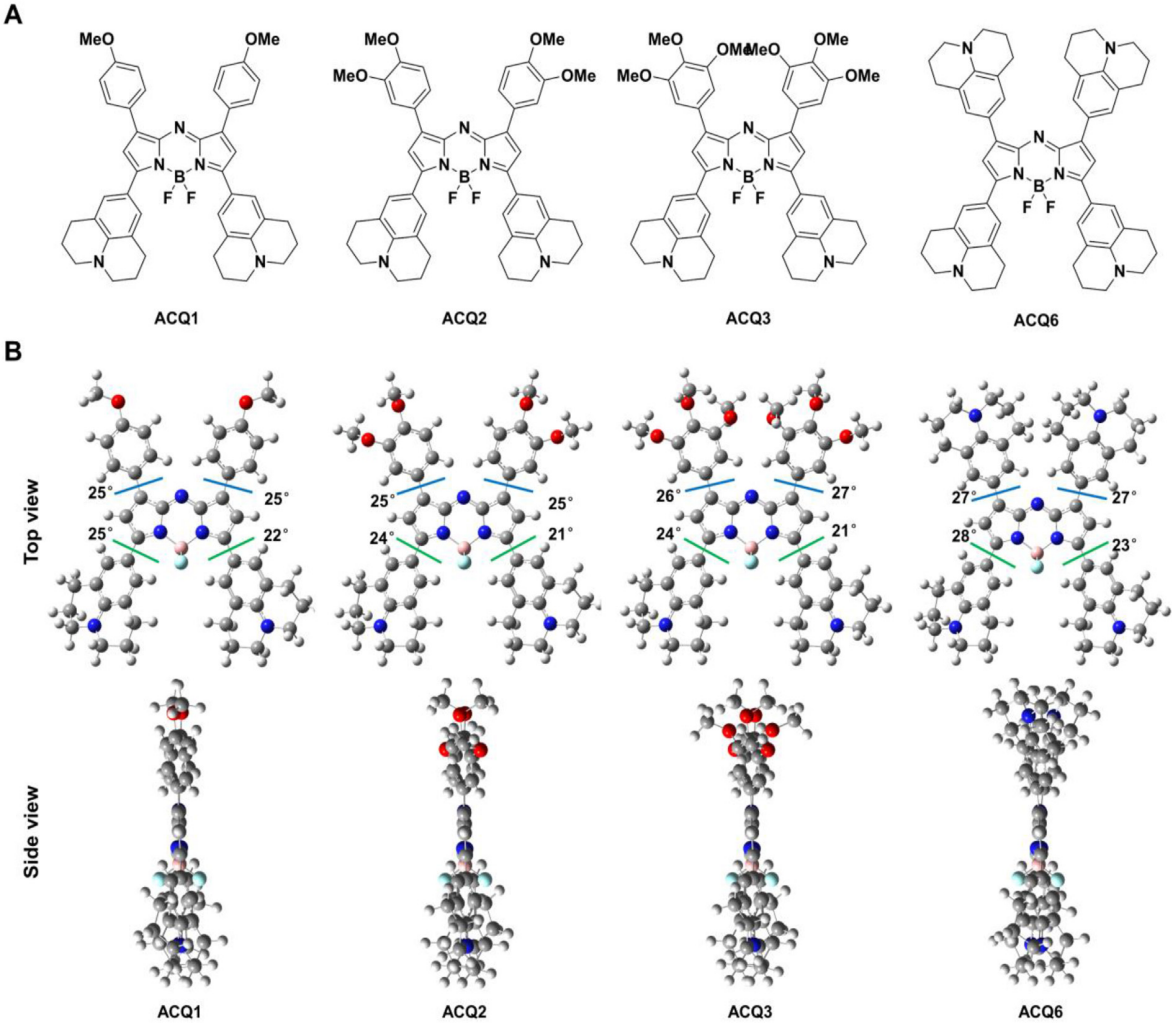
Theoretical calculations of ACQ1, ACQ2, ACQ3 and ACC6. (A) Chemical structures; (B) optimized ground-state (S_0_) geometries (top and side views) at the B3LYP/6-31+G (d, p) level of theory using the Gaussian 09 program.

To explain the effect of the 1,7-position modification on the ACQ properties, DFT calculations were performed on the optimal ACQ1 and ACQ6 with the inferior ACQ2 and ACQ3 as references utilizing a B3LYP/6-31+G (d, p) basis set. As shown in Fig. 3A and B, these probes showed a nearly coplanar configuration between the peripheral aromatic rings and the aza-BODIPY core with dihedral angles between 21° and 28° due to their high degree of conjugation. However, an increased number of methoxy groups in the 1,7-substituents resulted in a thicker top part, leading to reduced planarity and inferior ACQ properties (ACQ2/ACQ3 *vs.* ACQ1). For 1,7-julolidinyl ACQ6, the julolidinyl substituents did indeed increase the thickness of the top part, but this effect was overcompensated by the high aggregation tendency of julolidinyl, resulting in the most promising ACQ properties. This assumption can be supported by diminishing or disappearing signals from propylene (Supporting Information Fig. S12) and a higher melting point of julolidine (34 °C) than that of methoxybenzine (−37 °C).

### 3.5 NIR-II ACQ probe-labeled nanocarriers

**Figure 4:**
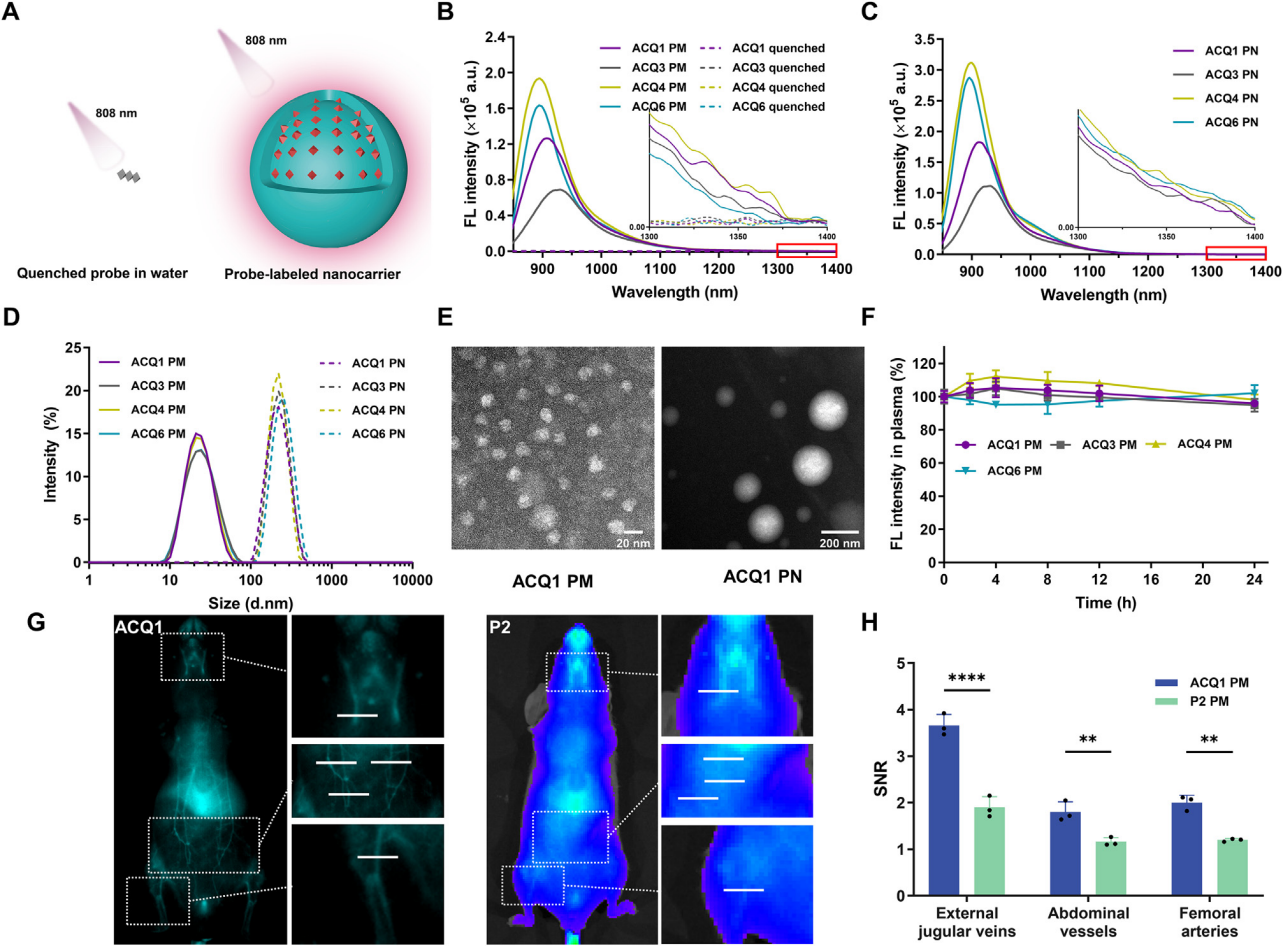
Characterization and fluorescence of 3,5-julolidinyl aza-BODIPY-labeled nanocarriers. (A) Schematic illustration of 3,5-julolidinyl aza-BODIPYs in water and nanocarriers. (B) Fluorescence spectrum of labeled PMs and quenched solution. Inset: zoom-in emission spectra in the range of 1300–1400 nm. (C) Fluorescent spectrum of labeled PNs. Inset: zoom-in emission spectra in the range of 1300–1400 nm. (D) Size distribution of the labeled nanocarriers. (E) TEM images of ACQ1-labeled nanocarriers. (F) Fluorescence stability of labeled PMs in plasma (*n* = 3; mean ± SD). (G) NIR-II (ACQ1-labeled PM, 808 nm excitation and 1300–1700 nm collection) and NIR-I (P2-labeled PM, 710 nm excitation and 750–770 nm collection) imaging of systemic vascular structures, including external jugular veins, femoral arteries, and skin surface vessels. (H) Corresponding vascular signal-to-noise ratio (SNR) analysis with intensity over the white lines in (G) (*n* = 3; mean ± SD; two-way ANOVA; ∗∗*P* < 0.01 and ∗∗∗∗*P* < 0.0001).

Inspired by their high brightness, promising water sensitivity, and outstanding quenching stability, optimized ACQ1 and ACQ6 were utilized to label model nanocarriers, highly dynamic mPEG_2k_-PDLLA_2k_ PMs with a core/shell structure and relatively stable PCL PNs with a matrical structure, by physical embedment with P2 as a reference. Moreover, nanocarriers labeled with inferior ACQ3 and ACQ4 were also prepared as counterevidence. To determine the loading capacity of probes in PMs and PNs, ACQ1 was selected as a model probe and concentration-related fluorescence intensity of labeled PMs and PNs was investigated. As shown in Supporting Information Fig. S13A and S13B, the fluorescence intensity of PMs and PNs in the region above 1000 nm increased with increasing weight fraction of ACQ1. A slight deviation from linearity was observed at weight fractions of 0.25% and 0.3% for PMs (0.535 μmol or 40 μg of ACQ1 in 160 mg of mPEG_2k_-PDLLA_2k_) and PNs (0.268 μmol or 20 μg of ACQ1 in 68 mg of PCL), respectively, which indicates the occurrence of concentration quenching. Although a higher probe loading may have a higher fluorescence intensity, the effect of concentration quenching would accelerate. Therefore, the current two weight fractions (0.25% and 0.3%) were selected in the formula to ensure loading efficiency and the final fluorescence intensity. As other probes have the same parent structure, similar modification moieties, and thereby similar physicochemical properties, the same loading levels are preset for other probes when labeling the nanocarriers. Ideal ACQ probes should be well dispersed and emit bright fluorescence in nanocarriers but should completely aggregate and be completely quenched in water (Fig. 4A). PMs labeled with ACQ probes exhibited intense absorption at 700–900 nm and bright emission centered at approximately 950 nm and tailed into the NIR-II window with quantum yields of 1.54%–2.01% in the region of 850–1400 nm and 0.21%–0.29% in the region of 1000–1400 nm (Supporting Information Table S2), and obvious signals even at a wavelength above 1300 nm, in contrast to broad and weak absorption and virtually no emission in water, demonstrating successful labeling (Fig. 4B and Supporting Information Fig. S13C‒S13F). Similarly, the PNs were also successfully labeled and demonstrated even brighter fluorescence than labeled PMs (Fig. 4C and Fig. S13C–S13F). Nevertheless, the quantum yields for PNs could not be correctly calculated because of significant light scattering of the PN dispersion that disenables measurement of absorption. A rough comparison between the integrated fluorescence intensity of PMs to that of PNs revealed higher quantum yield of PNs than that of PMs. Consistent with their brightness, the fluorescence of the brighter ACQ6-and ACQ4-labeled nanocarriers was higher than that of ACQ1, while the inferior ACQ3-labeled nanocarriers displayed slightly lower fluorescence. Although ACQ6 was brighter than ACQ1 in the region over 1300 nm in DMSO (Fig. 2A and C), ACQ6-labeled nanocarriers displayed weaker fluorescence in this region in PMs due to the lower polarity within the nanocarriers and the resulting blueshift in the spectrum. As shown in Fig. 4D, Supporting Information Tables S3 and S4, the hydrodynamic diameters of labeled PMs and PNs were monodispersed with approximate values of 21 and 210 nm, respectively; their zeta potentials were below ±10 and ± 3 mV, respectively. Transmission electron microscopy (TEM) revealed a spherical morphology and uniform size distribution of the labeled PMs and PNs (Fig. 4E). These probes were efficiently encapsulated in the nanocarriers with an EE of more than 90%, in contrast to the EE of 76.0%–81.6% for ACQ3. Moreover, the labeled PMs and PNs displayed superior fluorescence stability in PBS and plasma within 24 h (Fig. 4F and Fig. S13G‒S13I). Moreover, NIR-II imaging (ACQ1-labeled PMs) exhibited high spatial resolution with clear visualization of systemic vascular structures, whereas NIR-I imaging (P2-labeled PMs) revealed only fragments of fluorescent regions without detailed information, confirming the superiority of NIR-II imaging (Fig. 4G and H).

### 3.6 *In vivo* and *ex vivo* imaging of NIR-II ACQ probe-labeled nanocarriers

**Figure 5:**
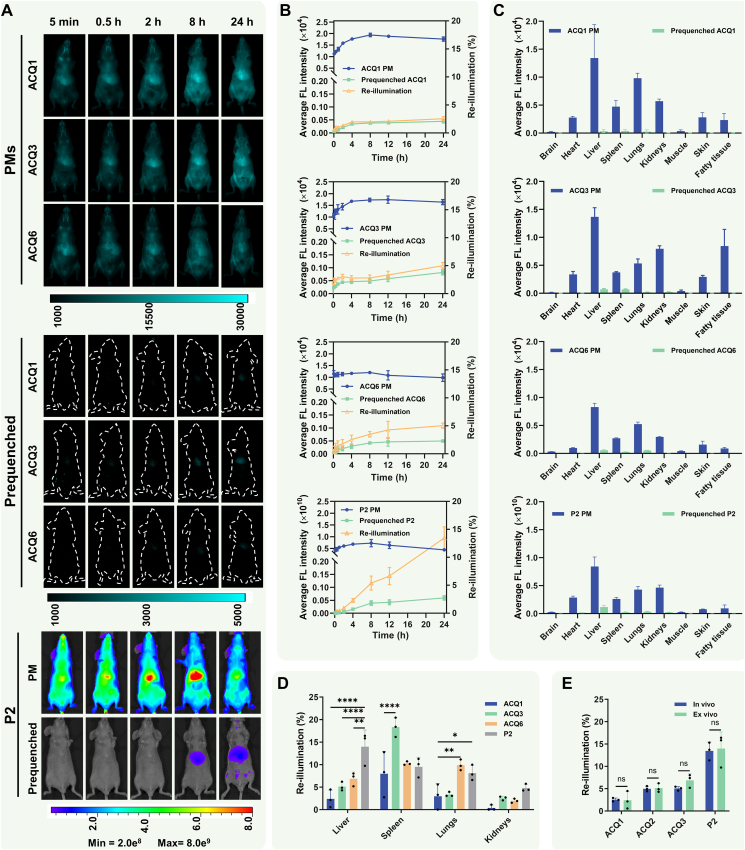
Fluorescence bioimaging of 3,5-julolidinyl aza-BODIPY-labeled PMs. (A) Fluorescence images of probe-labeled PMs and prequenched probes in mice after intravenous (i.v.) administration in the fasted state. *In vivo* NIR-II images using 808 nm excitation (43 mW/cm^2^) and 1300–1700 nm collection with 500 ms exposure time; NIR-I images using 710 excitation and 750–770 nm collection. (B) *In vivo* fluorescence intensity (left *Y*-axis) and reillumination (right *Y*-axis) in the hepatic regions after treatment with probe-labeled PMs and quenched probes (*n* = 3; mean ± SD). Hepatic reillumination was calculated as the ratio between *I*_PMs_ and *I*_prequenched_, wherein *I*_PMs_ and *I*_prequenched_ were the hepatic fluorescence intensity of probe-labeled PMs and prequenched probes, respectively. (C) *Ex vivo* fluorescence intensity for major organs and tissues after treatment with probe-labeled PMs and quenched probes (*n* = 3; mean ± SD). *Ex vivo* NIR-II images using 808 nm excitation (43 mW/cm^2^) and 1300–1700 nm collection with 300 ms exposure time; NIR-I images using 710 excitation and 750–770 nm collection. (D) *Ex vivo* reillumination proportion of probes in the liver, spleen, lungs, and kidneys (*n* = 3; mean ± SD; two-way ANOVA; ∗*P* < 0.05, ∗∗*P* < 0.01 and ∗∗∗∗*P* < 0.0001). (E) *In vivo* and *ex vivo* reillumination fraction of probes in the liver (*n* = 3; mean ± SD; two-way ANOVA; ns, not significant).

To demonstrate the superiority of NIR-II ACQ probes for real-time imaging of nanocarriers *in vivo*, the *in vivo* behavior of mPEG_2k_-PDLLA_2k_ PMs labeled with ACQ1 and ACQ6, with P2 and inferior ACQ3 as controls, was investigated after injection into mice through the tail vein (Fig. 5A and Supporting Information Figs. S14‒S18). Since longer wavelengths favor reduced scatter and autofluorescence^26^^,^^56^, ^57^, ^58^, ^59^, ^60^, fluorescence was monitored in the region above 1300 nm. Meanwhile, the *in vivo* reillumination of prequenched probes was also explored. To evaluate the interference caused by the reillumination signals of free probes, the fluorescence of labeled PMs and prequenched probes was measured in the hepatic regions where both are prone to accumulation, and the proportion of reillumination was calculated (Fig. 5B). The ACQ1-, ACQ3-, and ACQ6-labeled PMs displayed similar biodistribution within 24 h. The PMs were mainly distributed in vessels within 2 h after injection. Thereafter, the vascular signals gradually disappeared, while the hepatic retention of PMs increased and peaked at 8 h due to uptake by the mononuclear phagocyte system. In the case of P2, a similar biodistribution of PMs was observed, but only fragments of fluorescent regions were recorded, without revealing any detail. Contrary to conventional probes (DiR) with high reillumination^19^^,^^21^^,^^23^, no reillumination signals were observed at all (Figs. S14‒S16), and the estimated reillumination was less than 6% within 24 h for prequenched NIR-II probes due to their outstanding ACQ properties. After decreasing the maximum calibration bar, only a faint cyan color was observed at 24 h for ACQ1 with less than 2.5% fluorescence reillumination. However, reillumination signals of prequenched ACQ3 were observed at 2 h after administration, increased over time, and were up to 5% of the fluorescence signal in the corresponding PM group at 24 h. Although prequenched ACQ6 displayed low reillumination similar to that of ACQ1, its lower brightness in the PM group resulted in relatively higher interference from reillumination signals than that of ACQ1. The inferior *in vivo* ACQ property of ACQ6 was related to its low brightness after labeling in the PM (Fig. 4B) and slightly high fluorescence after PM disassembly and redispersion in a polar solvent (Fig. 2A and C) in the region above 1300 nm. The reillumination of prequenched P2 was evident with nonnegligible fake signals at 8 h after injection. The fake signals increased with time up to 14% of the PM fluorescence at 24 h, which inevitably interferes with the exploration of the *in vivo* fate of nanocarriers.

To investigate the biodistribution of PMs and interference of free probes *ex vivo*, the mice were sacrificed at 24 h, and various organs and tissues were dissected and imaged. As shown in Fig. 5C, all labeled PMs displayed similar biodistribution tendencies, with major distribution in the liver, spleen, lungs, and kidneys; moderate distribution in the heart, skin, and adipose tissue; and limited distribution in the brain and muscle, consistent with the *in vivo* results. After injection with prequenched NIR-II probes, almost no fluorescence signals were observed in organs and tissues, except for the liver, spleen, and lungs, where only faint fluorescence was detected. The interference of reillumination was minimal for ACQ1, while the reillumination percentage of ACQ3 and ACQ6 in the liver, spleen, and lungs increased due to high fluorescence recovery for ACQ3 and low fluorescence intensity in the corresponding PM group for ACQ6. Although there was similar reillumination of prequenched ACQ3/ACQ6 in the spleen or lungs compared to P2, the NIR-II probes displayed a lower reillumination percentage in the liver where reillumination is more likely to occur (Fig. 5D). The reillumination percentage of ACQ1 in all easily reilluminated organs was dramatically reduced compared to that of P2, indicating its potential application as an ACQ NIR-II probe. Moreover, there were almost the same hepatic reillumination proportions *in vivo* and *ex vivo*, indicating their consistency in the reillumination investigation (Fig. 5E).

## 4 Conclusions

To harness the high spatiotemporal resolution and deep penetration of NIR-II imaging, highly planar and electron-rich julolidine was installed at the 3,5-position (with larger substituent effects) of aza-BODIPY, and analogs with fine-tuning planarity and hydrophobicity were constructed to develop NIR-II ACQ probes. The newly developed probes displayed intense absorption centered at approximately 850 nm with a molar extinction coefficient of 60,000–83,000 L/(mol·cm), emitted bright fluorescence in the 950–1300 nm region with quantum yields of 1.50%–5.23%, and even exhibited obvious fluorescence signals in the region above 1300 nm. Most of the 3,5-julolidinyl probes were more sensitive to water than NJ1060 and NIR-I ACQ probe P2. The reillumination of the 3,5-julolidinyl probes was significantly reduced in plasma and 1% Tween compared to P2. ACQ1 and ACQ6 displayed greater water sensitivity and quenching stability with absolute fluorescence quenching at 40% water and less than 2.0% fluorescence recovery in plasma after 24 h incubation. Molecular planarity was more important than hydrophobicity for the ACQ properties. Theoretical calculations confirmed the importance of molecular planarity. Additionally, the *in vivo* biodistribution of the biodegradable PMs illustrated minimal artifacts from reilluminated ACQ1 in the hepatic region, with a value less than 2.5%, which was significantly reduced compared to almost 15% observed for P2. *Ex vivo* imaging assessments also verified low artifacts from ACQ1.

## Author contributions

Wei Wu, and Haisheng He conceived and designed this project. Chang Liu performed the chemical and photophysical experiments and wrote the draft manuscript. Yifan Cai performed the *in vitro* and *in vivo* experiments. Zichen Zhang, Yi Lu and Quangang Zhu assisted in both *in vitro* and *in vivo* experiment. Wei Wu, Weili Zhao, Zhongjian Chen and Haisheng He supervised this study. Zhongjian Chen and Weili Zhao provided facilities and resources. Wei Wu obtained financial supports. Wei Wu and Haisheng He edited the manuscript. All authors have given approval to the final version of the manuscript.

## Conflicts of interest

The authors declare no competing financial interests.

## Appendix A Supporting information

The following is the Supporting Information to this article.
