# Supplementary material for: Julolidinyl aza-BODIPYs as NIR-II fluorophores for the bioimaging of nanocarriers
Source: Acta Pharm Sin B. 2024 Apr 10;14(7):3155–68. doi: 10.1016/j.apsb.2024.04.002 (PMC11252509; doi:10.1016/j.apsb.2024.04.002)
Supplement: Multimedia component 1 [file mmc1.pdf]

Supporting Information for

ORIGINAL ARTICLE

Julolidinyl aza-BODIPYs as NIR-II fluorophores for the bioimaging of nanocarriers

Chang Liu[a,b,†], Yifan Cai[b,†], Zichen Zhang[b], Yi Lu[a], Quangang Zhu[a], Haisheng He[b,]*, Zhongjian Chen[a,]*, Weili Zhao[a,c,]*, Wei Wu[a,b,]*

[a]*Shanghai Skin Disease Hospital, Tongji University School of Medicine, Shanghai 200443, China*

[b]*Key Laboratory of Smart Drug Delivery of MOE, School of Pharmacy, Fudan University, Shanghai 201203, China*

[c]*Department of MediChinal Chemistry, School of Pharmacy, Fudan University, Shanghai 201203, China*

Received 16 January 2024; received in revised form 5 March 2024; accepted 28 March 2024

*Corresponding authors.

E-mail addresses: he_haisheng@fudan.edu.cn (Haisheng He), aajian818@163.com (Zhongjian Chen), zhaoweili@fudan.edu.cn (Weili Zhao), wuwei@shmu.edu.cn (Wei Wu).

[†]These authors made equal contributions to this work.

1. Chemistry and experimental instruments

^{1}H NMR and ^{13}C NMR spectra (Figs. S19-S60) of newly synthesized compounds are attached to the end of the Supporting Information.

1.1. Synthesis of ACQ1

9-acetyljulolidine: To a mixture of *N*,*N*-dimethylacetamide (0.81 mL, 8.8 mmol) in dry 1,2-dichloroethane (40 mL) was added phosphorus oxychloride (0.68 mL, 7.3 mmol) under nitrogen at 0 °C. After stirring at room temperature for 30 min, julolidine (1000 mg, 5.8 mmol) in 1,2-dichloroethane (10 mL) was added. The reaction was heated to 80 °C, and the reaction progress was monitored by thin-layer chromatography (TLC). After full conversion, the reaction was quenched with sodium carbonate (aq) and extracted with dichloromethane. The organic phase was dried over

anhydrous sodium sulfate and concentrated under vacuum. The crude material was purified by silica gel flash column chromatography to harvest 9-acetyljulolidine as a light yellow solid (200 mg, 16%). ^{1}H NMR (600 MHz, $CDCl_3$) δ 7.43 (s, 2H), 3.32–3.19 (m, 4H), 2.75 (t, J = 6.3 Hz, 4H), 2.45 (s, 3H), 2.00–1.91 (m, 4H). ^{13}C NMR (151 MHz, $CDCl_3$) δ 196.50, 146.85, 128.22, 124.29, 119.93, 50.07, 27.89, 25.95, 21.61 (Fig. S19).

I-1: To a mixture of 9-acetyljulolidine (989 mg, 4.6 mmol) and *p*-methoxybenzaldehyde (694 mg, 5.1 mmol) in ethanol (20 mL) was added potassium hydroxide (aq, 5 mL, 50%). The reaction was stirred at room temperature, and the reaction progress was monitored by TLC. After full conversion, the reaction was quenched with ice, followed by filtration and recrystallization in aqueous ethanol to obtain I-1 in 71% as a yellow solid (1100 mg). ^{1}H NMR (600 MHz, $CDCl_3$) δ 7.73 (d, J = 15.5 Hz, 1H), 7.59 (d, J = 8.7 Hz, 2H), 7.57 (s, 2H), 7.45 (d, J = 15.5 Hz, 1H), 6.92 (d, J = 8.7 Hz, 2H), 3.85 (s, 3H), 3.31–3.24 (m, 4H), 2.80 (t, J = 6.3 Hz, 4H), 2.03–1.93 (m, 4H). ^{13}C NMR (151 MHz, $CDCl_3$) δ 187.69, 161.15, 146.85, 141.80, 129.95, 128.61, 128.42, 125.21, 120.24, 120.17, 114.38, 55.52, 50.13, 27.95, 21.64 (Fig. S20).

II-1: To a mixture of I-1 (932 mg, 2.8 mmol) in nitromethane (10 mL) and methanol (50 mL) was added 1,8-diazabicyclo[5.4.0]undecane-7-ene (2.1 mL), and the reaction was refluxed. After 7 h, the reaction was quenched with ice water and extracted with dichloromethane. The organic phase was dried over anhydrous sodium sulfate and concentrated under vacuum. The crude material was purified by silica gel flash column chromatography to obtain II-1 in 92% as a light yellow solid (1010 mg). ^{1}H NMR (600 MHz, $CDCl_3$) δ 7.38 (s, 2H), 7.20 (d, J = 8.6 Hz, 2H), 6.84 (d, J = 8.6 Hz, 2H), 4.85–4.76 (m, 1H), 4.65–4.58 (m, 1H), 4.18–4.09 (m, 1H), 3.37–3.10 (m, 6H), 2.78–2.68 (m, 4H), 2.00–1.86 (m, 4H). ^{13}C NMR (151 MHz, $CDCl_3$) δ 194.68, 159.06, 147.20, 131.87, 128.66, 127.96, 123.30, 120.02, 114.44, 80.17, 55.39, 50.07, 40.86, 39.26, 27.89, 21.51 (Fig. S21).

ACQ1: To a mixture of II-1 (591 mg, 1.5 mmol) in *n*-butanol (20 mL) was added ammonium acetate (3126 mg, 40.6 mmol), and the reaction was refluxed. After 12 h, the reaction was cooled and filtered to obtain a precursor of aza-BODIPY. Boron trifluoride etherate (1.2 mL) and *N*,*N*-diisopropylethylamine (1.2 mL) were added to the solution of the precursor in dry 1,2-dichloroethane (100 mL) at room temperature.

The mixture was heated to 80 °C, and the reaction progress was monitored by TLC. After full conversion, the mixture was concentrated under vacuum and purified by silica gel flash column chromatography to obtain ACQ1 in 29% as a black solid (163 mg). ^{1}H NMR (600 MHz, DMSO-d_6) δ 8.14 (d, J = 8.8 Hz, 4H), 7.81 (s, 4H), 7.45 (s, 2H), 7.08 (d, J = 8.9 Hz, 4H), 3.85 (s, 6H), 3.33 (d, J = 7.9 Hz, 8H), 2.75 (t, J = 6.2 Hz, 8H), 1.96–1.90 (m, 8H). ^{13}C NMR (151 MHz, DMSO-d_6) δ 159.77, 153.45, 145.21, 143.59, 137.69, 129.96, 129.10, 125.31, 120.54, 116.82, 116.65, 114.08, 55.31, 49.45, 27.26, 21.07 (Fig. S22). ESI-HRMS (m/z): $[M]^+$ Calcd. for $[C_{46}H_{44}BF_2N_5O_2]^+$: 747.3551, found: 747.3550.

1.2. Synthesis of ACQ2

I-2: The title compound was prepared according to the same method of I-1 from 9-acetyljulolidine (989 mg, 4.6 mmol) and *m,p*-dimethoxybenzaldehyde (847 mg, 5.1 mmol) in 78% as a yellow solid (1300 mg). ^{1}H NMR (600 MHz, $CDCl_3$) δ 7.71 (d, J = 15.5 Hz, 1H), 7.57 (s, 2H), 7.42 (d, J = 15.5 Hz, 1H), 7.23 (dd, J = 8.3, 1.6 Hz, 1H), 7.16 (d, J = 1.5 Hz, 1H), 6.90 (t, J = 8.5 Hz, 1H), 3.97 (d, J = 11.1 Hz, 3H), 3.92 (d, J = 9.8 Hz, 3H), 3.28 (dd, J = 13.6, 7.9 Hz, 4H), 2.84–2.77 (m, 4H), 2.02–1.94 (m, 4H). ^{13}C NMR (151 MHz, $CDCl_3$) δ 187.67, 150.87, 149.27, 146.88, 142.17, 128.88, 128.43, 125.16, 122.54, 120.50, 120.19, 111.25, 110.40, 56.16, 56.12, 50.13, 27.96, 21.63 (Fig. S23).

II-2: The title compound was prepared according to the same method of II-1 from I-1 (1016 mg, 2.8 mmol) in 83% as a yellow solid (990 mg). ^{1}H NMR (600 MHz, $CDCl_3$) δ 7.38 (s, 2H), 6.83–6.79 (m, 2H), 6.78 (s, 1H), 4.81 (dt, J = 15.0, 7.5 Hz, 1H), 4.63 (dd, J = 12.4, 8.7 Hz, 1H), 4.16–4.08 (m, 1H), 3.87 (d, J = 5.8 Hz, 3H), 3.84 (s, 3H), 3.31–3.16 (m, 6H), 2.73 (t, J = 6.3 Hz, 4H), 1.98–1.90 (m, 4H). ^{13}C NMR (151 MHz, $CDCl_3$) δ 194.69, 149.25, 148.53, 147.22, 132.42, 127.97, 123.29, 120.02, 119.33, 111.59, 111.22, 80.08, 56.10, 56.02, 50.07, 40.87, 39.72, 27.89, 21.50 (Fig. S24).

ACQ2: The title compound was prepared according to the same method of ACQ1 from II-2 (636 mg, 1.5 mmol) in 23% as a black solid (138 mg). ^{1}H NMR (600 MHz, DMSO-d_6) δ 7.81 (s, 4H), 7.76 (dd, J = 8.4, 1.7 Hz, 2H), 7.62 (d, J = 1.6 Hz, 2H), 7.45 (s, 2H), 7.04 (d, J = 8.5 Hz, 2H), 3.84 (s, 6H), 3.75 (s, 6H), 3.36–3.32 (m, 8H), 2.76 (t, J = 6.2 Hz, 8H), 1.98–1.89 (m, 8H). ^{13}C NMR (151 MHz, DMSO) δ 153.30, 149.54, 148.72, 145.19, 143.66, 138.22, 129.11, 125.68, 121.81, 120.52,

117.18, 116.84, 112.25, 111.70, 55.62, 55.59, 49.45, 27.28, 21.07 (Fig. S25). ESI-HRMS (m/z): $[M]^+$ Calcd. for $[C_{48}H_{48}BF_2N_5O_4]^+$: 807.3762, found: 807.3765.

1.3. Synthesis of ACQ3

I-3: The title compound was prepared according to the same method of I-1 from 9-acetyljulolidine (989 mg, 4.6 mmol) and *m,m,p*-trimethoxybenzaldehyde (1000 mg, 5.1 mmol) in 66% as a yellow solid (1200 mg). ^{1}H NMR (600 MHz, $CDCl_3$) δ 7.67 (d, J = 15.5 Hz, 1H), 7.56 (s, 2H), 7.42 (d, J = 15.5 Hz, 1H), 6.86 (s, 2H), 3.93 (s, 6H), 3.89 (s, 3H), 3.33–3.25 (m, 4H), 2.80 (t, J = 6.3 Hz, 4H), 2.00–1.95 (m, 4H). ^{13}C NMR (151 MHz, $CDCl_3$) δ 187.49, 153.55, 147.00, 142.21, 139.91, 131.43, 128.49, 124.95, 121.87, 120.21, 105.56, 61.13, 56.40, 50.14, 27.96, 21.60 (Fig. S26).

II-3: The title compound was prepared according to the same method of II-1 from I-3 (1100 mg, 2.8 mmol) in 72% as a yellow solid (920 mg). ^{1}H NMR (600 MHz, $CDCl_3$) δ 7.38 (s, 2H), 6.47 (s, 2H), 4.83 (dd, J = 12.6, 6.1 Hz, 1H), 4.65 (dd, J = 12.6, 8.8 Hz, 1H), 4.16–4.09 (m, 1H), 3.84 (s, 6H), 3.81 (s, 3H), 3.33–3.14 (m, 6H), 2.77–2.69 (m, 4H), 1.98–1.88 (m, 4H). ^{13}C NMR (151 MHz, $CDCl_3$) δ 194.56, 153.58, 147.26, 135.61, 127.97, 123.23, 120.03, 104.69, 79.77, 60.94, 56.34, 50.07, 40.82, 40.32, 27.89, 21.49 (Fig. S27).

ACQ3: The title compound was prepared according to the same method of ACQ1 from II-3 (681 mg, 1.5 mmol) in 25% as a black solid (162 mg). ^{1}H NMR (600 MHz, pyridine-d_5) δ 8.32 (s, 4H), 7.80 (s, 2H), 7.67 (s, 4H), 4.02 (s, 6H), 3.80 (d, J = 6.7 Hz, 12H), 3.07–3.01 (m, 8H), 2.76 (t, J = 6.2 Hz, 8H), 1.80–1.72 (m, 8H). ^{13}C NMR (151 MHz, pyridine-d_5) δ 155.82, 154.41, 146.31, 145.85, 140.62, 140.00, 130.72, 130.14, 121.85, 119.29, 119.12, 107.86, 61.21, 56.52, 50.46, 28.46, 22.16 (Fig. S28). ESI-HRMS (m/z): $[M]^+$ Calcd. for $[C_{50}H_{52}BF_2N_5O_6]^+$: 867.3973, found: 867.3976.

1.4. Synthesis of ACQ4

I-4: The title compound was prepared according to the same method of I-1 from 9-acetyljulolidine (989 mg, 4.6 mmol) and *o,p*-dimethoxybenzaldehyde (847 mg, 5.1 mmol) in 75% as a yellow solid (1250 mg). ^{1}H NMR (600 MHz, $CDCl_3$) δ 8.00 (d, J = 15.7 Hz, 1H), 7.58 (d, J = 8.5 Hz, 1H), 7.57–7.51 (m, 3H), 6.52 (d, J = 8.6 Hz, 1H), 6.47 (s, 1H), 3.89 (s, 3H), 3.85 (s, 3H), 3.30–3.22 (m, 4H), 2.79 (t, J = 6.1 Hz, 4H), 2.01–1.93 (m, 4H). ^{13}C NMR (151 MHz, $CDCl_3$) δ 188.40, 162.47, 160.14, 146.65,

137.51, 130.26, 128.42, 125.61, 120.95, 120.12, 118.11, 105.36, 98.57, 55.68, 55.60, 50.13, 27.97, 21.70 (Fig. S29).

II-4: The title compound was prepared according to the same method of II-1 from I-4 (1016 mg, 2.8 mmol) in 88% as a yellow solid (1050 mg). ^{1}H NMR (600 MHz, $CDCl_3$) δ 7.41 (s, 2H), 7.10 (d, J = 8.3 Hz, 1H), 6.44 (s, 1H), 6.41 (d, J = 8.4 Hz, 1H), 4.81 (d, J = 7.0 Hz, 2H), 4.30 (p, J = 7.0 Hz, 1H), 3.84 (s, 3H), 3.77 (s, 3H), 3.35–3.23 (m, 6H), 2.74 (t, J = 6.1 Hz, 4H), 1.98–1.91 (m, 4H). ^{13}C NMR (151 MHz, $CDCl_3$) δ 195.60, 160.35, 158.41, 147.05, 129.98, 127.99, 123.64, 119.98, 119.92, 104.41, 99.25, 78.38, 55.53, 55.47, 50.08, 39.08, 36.00, 27.91, 21.57 (Fig. S30).

ACQ4: The title compound was prepared according to the same method of ACQ1 from II-4 (636 mg, 1.5 mmol) in 34% as a black solid (205 mg). ^{1}H NMR (600 MHz, pyridine-d_5) δ 8.60 (d, J = 8.6 Hz, 2H), 8.20 (s, 4H), 7.77 (s, 2H), 6.87 (d, J = 8.6 Hz, 2H), 6.77 (s, 2H), 3.81 (d, J = 22.6 Hz, 6H), 3.79 (s, 6H), 3.00 (s, 8H), 2.74 (t, J = 5.7 Hz, 8H), 1.74 (d, J = 5.2 Hz, 8H). ^{13}C NMR (151 MHz, pyridine-d_5) δ 162.32, 160.53, 156.02, 146.48, 145.91, 137.11, 135.03, 130.39, 121.67, 119.67, 116.17, 106.14, 99.44, 56.10, 55.83, 50.40, 28.47, 22.21 (Fig. S31). ESI-HRMS (m/z): $[M]^+$ Calcd. for $[C_{48}H_{48}BF_2N_5O_4]^+$: 807.3762, found: 807.3762.

1.5. Synthesis of ACQ5

I-5: The title compound was prepared according to the same method of I-1 from 9-acetyljulolidine (989 mg, 4.6 mmol) and *p*-(dimethylamino)benzaldehyde (760 mg, 5.1 mmol) in 74% as a yellow solid (1180 mg). ^{1}H NMR (600 MHz, $CDCl_3$) δ 7.73 (d, J = 15.4 Hz, 1H), 7.61–7.51 (m, 4H), 7.38 (d, J = 15.4 Hz, 1H), 6.70 (d, J = 8.8 Hz, 2H), 3.32–3.21 (m, 4H), 3.03 (s, 6H), 2.80 (t, J = 6.3 Hz, 4H), 2.01–1.92 (m, 4H). ^{13}C NMR (151 MHz, $CDCl_3$) δ 187.99, 151.67, 146.57, 142.90, 130.04, 128.28, 125.71, 123.75, 120.13, 117.52, 112.03, 50.13, 40.35, 27.96, 21.71 (Fig. S32).

II-5: The title compound was prepared according to the same method of II-1 from I-5 (969 mg, 2.8 mmol) in 69% as a yellow solid (788 mg). ^{1}H NMR (600 MHz, $CDCl_3$) δ 7.39 (s, 2H), 7.14 (d, J = 8.7 Hz, 2H), 6.67 (d, J = 8.7 Hz, 2H), 4.80 (dt, J = 14.5, 7.3 Hz, 1H), 4.64–4.56 (m, 1H), 4.12–4.03 (m, 1H), 3.30–3.16 (m, 6H), 2.91 (s, 6H), 2.73 (t, J = 6.3 Hz, 4H), 1.94 (dd, J = 11.9, 6.1 Hz, 4H). ^{13}C NMR (151 MHz, $CDCl_3$) δ 195.08, 150.08, 147.13, 128.22, 127.99, 127.42, 123.43, 120.01, 112.97, 80.29, 50.08, 41.05, 40.67, 39.23, 27.89, 21.54 (Fig. S33).

ACQ5: The title compound was prepared according to the same method of ACQ1 from II-5 (611mg, 1.5 mmol) in 19% as a black solid (110 mg). ^{1}H NMR (600 MHz, pyridine-d_5) δ 8.48 (d, J = 8.1 Hz, 4H), 8.20 (s, 4H), 7.48 (s, 2H), 6.96 (d, J = 8.1 Hz, 4H), 3.01 (s, 8H), 2.88 (s, 12H), 2.78 (s, 8H), 1.76 (s, 8H). ^{13}C NMR (151 MHz, pyridine-d_5) δ 156.15, 151.55, 145.83, 145.77, 141.25, 131.32, 130.29, 122.70, 121.56, 119.78, 115.94, 113.00, 50.40, 40.37, 28.51, 22.28 (Fig. S34). ESI-HRMS (m/z): $[M]^+$ Calcd. for $[C_{48}H_{50}BF_2N_7]^+$: 773.4183, found: 773.4188.

1.6. Synthesis of ACQ6

I-6: The title compound was prepared according to the same method of I-1 from 9-acetyljulolidine (989 mg, 4.6 mmol) and 9-formyljulolidine (1025 mg, 5.1 mmol) in 71% as a yellow solid (1300 mg). ^{1}H NMR (600 MHz, $CDCl_3$) δ 7.65 (d, J = 15.3 Hz, 1H), 7.56 (s, 2H), 7.30 (d, J = 15.3 Hz, 1H), 7.11 (s, 2H), 3.31–3.16 (m, 7H), 2.84–2.72 (m, 7H), 2.03–1.91 (m, 7H). ^{13}C NMR (151 MHz, $CDCl_3$) δ 187.99, 146.43, 144.71, 143.47, 128.21, 127.82, 125.96, 122.68, 121.14, 120.10, 116.37, 50.13, 50.11, 27.97, 27.86, 21.86, 21.75 (Fig. S35).

II-6: The title compound was prepared according to the same method of II-1 from I-6 (1113 mg, 2.8 mmol) in 78% as a yellow solid (998 mg). ^{1}H NMR (600 MHz, $CDCl_3$) δ 7.40 (s, 2H), 6.65 (s, 2H), 4.77 (dd, J = 12.3, 6.1 Hz, 1H), 4.56 (dd, J = 12.3, 8.6 Hz, 1H), 3.98–3.90 (m, 1H), 3.30–3.24 (m, 4H), 3.24–3.20 (m, 1H), 3.13 (dd, J = 16.7, 8.9 Hz, 1H), 3.11–3.07 (m, 4H), 2.72 (dt, J = 13.4, 6.4 Hz, 8H), 1.97–1.88 (m, 8H). ^{13}C NMR (151 MHz, $CDCl_3$) δ 195.32, 147.07, 142.44, 128.00, 126.72, 125.96, 123.50, 121.84, 119.99, 80.21, 50.07, 41.25, 39.25, 27.89, 27.80, 22.15, 21.55 (Fig. S36).

ACQ6: The title compound was prepared according to the same method of ACQ1 from II-6 (689 mg, 1.5 mmol) in 15% as a black solid (100 mg). ^{1}H NMR (600 MHz, pyridine-d_5) δ 8.21 (s, 4H), 7.96 (s, 4H), 7.44 (s, 2H), 3.08 (s, 8H), 3.00 (s, 8H), 2.76 (d, J = 25.2 Hz, 16H), 1.85 (s, 8H), 1.76 (s, 8H). ^{13}C NMR (151 MHz, pyridine-d_5) δ 155.79, 145.83, 145.55, 144.29, 141.78, 130.21, 129.24, 122.17, 121.88, 121.47, 120.06, 115.46, 50.51, 50.37, 28.73, 28.53, 22.60, 22.33 (Fig. S37). ESI-HRMS (m/z): $[M]^+$ Calcd. for $[C_{56}H_{58}BF_2N_7]^+$: 877.4809, found: 877.4818.

1.7. Synthesis of ACQ7

I-7: The title compound was prepared according to the same method of I-1 from 9-acetyljulolidine (989 mg, 4.6 mmol) and benzaldehyde (541 mg, 5.1 mmol) in 68% as

a yellow solid (950 mg). ^{1}H NMR (600 MHz, $CDCl_3$) δ 7.75 (d, J = 15.6 Hz, 1H), 7.64 (d, J = 7.3 Hz, 2H), 7.56 (d, J = 14.0 Hz, 3H), 7.43–7.35 (m, 3H), 3.34–3.22 (m, 4H), 2.80 (t, J = 6.3 Hz, 4H), 2.02–1.92 (m, 4H). ^{13}C NMR (151 MHz, $CDCl_3$) δ 187.57, 147.01, 141.98, 135.87, 129.84, 128.93, 128.53, 128.31, 124.96, 122.54, 120.20, 50.14, 27.95, 21.61 (Fig. S38).

II-7: The title compound was prepared according to the same method of II-1 from I-7 (848 mg, 2.8 mmol) in 77% as a yellow solid (780 mg). ^{1}H NMR (600 MHz, $CDCl_3$) δ 7.40 (d, J = 12.3 Hz, 2H), 7.32 (t, J = 7.5 Hz, 2H), 7.28 (d, J = 7.0 Hz, 2H), 7.26–7.22 (m, 1H), 4.85 (dd, J = 12.5, 6.0 Hz, 1H), 4.74–4.62 (m, 1H), 4.23–4.14 (m, 1H), 3.39–3.19 (m, 6H), 2.74 (dd, J = 15.5, 9.2 Hz, 4H), 2.01–1.89 (m, 4H). ^{13}C NMR (151 MHz, $CDCl_3$) δ 194.52, 147.22, 139.98, 129.07, 127.95, 127.74, 127.63, 123.24, 120.03, 79.87, 50.08, 40.74, 39.89, 27.89, 21.51 (Fig. S39).

ACQ7: The title compound was prepared according to the same method of ACQ1 from II-7 (546 mg, 1.5 mmol) in 21% as a black solid (110 mg). ^{1}H NMR (600 MHz, pyridine-d_5) δ 8.37 (d, J = 7.7 Hz, 4H), 8.24 (s, 4H), 7.62 (s, 2H), 7.55 (t, J = 7.7 Hz, 4H), 7.45 (t, J = 7.3 Hz, 2H), 3.08–2.98 (m, 8H), 2.79 (t, J = 6.2 Hz, 8H), 1.80–1.70 (m, 8H). ^{13}C NMR (151 MHz, pyridine-d_5) δ 156.03, 146.37, 145.90, 140.04, 134.40, 130.67, 130.00, 129.33, 129.25, 121.86, 119.28, 119.07, 50.47, 28.47, 22.14 (Fig. S40). ESI-HRMS (m/z): $[M]^+$ Calcd. for $[C_{44}H_{40}BF_2N_5]^+$: 687.3339, found: 687.3345.

1.8. Synthesis of ACQ8

I-8: The title compound was prepared according to the same method of I-1 from 9-acetyljulolidine (989 mg, 4.6 mmol) and 2-thenaldehyde (571 mg, 5.1 mmol) in 84% as a yellow solid (1200 mg). ^{1}H NMR (600 MHz, $CDCl_3$) δ 7.88 (d, J = 15.2 Hz, 1H), 7.55 (s, 2H), 7.39–7.33 (m, 2H), 7.30 (d, J = 3.5 Hz, 1H), 7.09–7.04 (m, 1H), 3.27 (dd, J = 13.4, 7.7 Hz, 4H), 2.83–2.74 (m, 4H), 2.03–1.91 (m, 4H). ^{13}C NMR (151 MHz, $CDCl_3$) δ 186.96, 147.01, 141.37, 134.57, 130.93, 128.43, 128.24, 127.62, 124.85, 121.46, 120.20, 50.14, 27.94, 21.60 (Fig. S41).

II-8: The title compound was prepared according to the same method of II-1 from I-8 (865 mg, 2.8 mmol) in 74% as a yellow solid (770 mg). ^{1}H NMR (600 MHz, $CDCl_3$) δ 7.41 (s, 2H), 7.19 (d, J = 5.0 Hz, 1H), 6.95 (d, J = 3.2 Hz, 1H), 6.93 (dd, J = 5.0, 3.6 Hz, 1H), 4.86 (dd, J = 12.6, 5.7 Hz, 1H), 4.66 (dd, J = 12.6, 8.2 Hz, 1H), 4.56–4.47 (m, 1H), 3.34 (ddd, J = 24.9, 16.9, 6.8 Hz, 2H), 3.27 (dd, J = 9.9, 4.2 Hz,

4H), 2.74 (t, *J* = 6.3 Hz, 4H), 1.99–1.90 (m, 4H). ^{13}C NMR (151 MHz, $CDCl_3$) *δ* 194.00, 147.32, 142.89, 127.99, 127.17, 125.50, 124.59, 123.10, 120.06, 80.31, 50.09, 41.44, 35.37, 27.89, 21.50 (Fig. S42).

ACQ8: The title compound was prepared according to the same method of ACQ1 from II-8 (555 mg, 1.5 mmol) in 10% as a black solid (50 mg). ^{1}H NMR (600 MHz, pyridine-d_5) *δ* 8.23 (s, 2H), 8.17 (s, 4H), 7.66 (s, 2H), 7.27 (s, 4H), 3.03 (s, 8H), 2.75 (d, *J* = 5.4 Hz, 8H), 1.74 (s, 8H). ^{13}C NMR (151 MHz, pyridine-d_5) *δ* 162.34, 156.16, 146.36, 145.26, 134.69, 130.61, 129.40, 129.10, 128.82, 121.81, 119.00, 117.24, 50.46, 28.43, 22.12 (Fig. S43). ESI-HRMS (m/z): $[M]^+$ Calcd. for $[C_{40}H_{36}BF_2N_5S_2]^+$: 699.2468, found: 699.2472.

1.9. Synthesis of ACQ9

I-9: The title compound was prepared according to the same method of I-1 from 9-acetyljulolidine (989 mg, 4.6 mmol) and *p*-ethoxybenzaldehyde (765 mg, 5.1 mmol) in 81% as a yellow solid (1300 mg). ^{1}H NMR (600 MHz, $CDCl_3$) *δ* 7.72 (d, *J* = 15.5 Hz, 1H), 7.58 (d, *J* = 8.7 Hz, 2H), 7.57 (s, 2H), 7.47–7.42 (m, 1H), 6.91 (d, *J* = 8.7 Hz, 2H), 4.12–3.99 (m, 2H), 3.32–3.24 (m, 4H), 2.80 (t, *J* = 6.3 Hz, 4H), 2.03–1.92 (m, 4H), 1.46–1.38 (m, 3H). ^{13}C NMR (151 MHz, $CDCl_3$) *δ* 187.71, 160.57, 146.83, 141.89, 129.96, 128.41, 125.24, 120.17, 120.09, 114.87, 63.72, 50.13, 27.95, 21.65, 14.91 (Fig. S44).

II-9: The title compound was prepared according to the same method of II-1 from I-9 (972 mg, 2.8 mmol) in 84% as a yellow solid (960 mg). ^{1}H NMR (600 MHz, $CDCl_3$) *δ* 7.38 (s, 2H), 7.18 (d, *J* = 8.6 Hz, 2H), 6.83 (t, *J* = 5.8 Hz, 2H), 4.81 (dd, *J* = 12.4, 6.0 Hz, 1H), 4.61 (dd, *J* = 12.3, 8.7 Hz, 1H), 4.17–4.06 (m, 1H), 3.99 (q, *J* = 7.0 Hz, 2H), 3.30–3.15 (m, 6H), 2.73 (t, *J* = 6.3 Hz, 4H), 2.00–1.89 (m, 4H), 1.39 (t, *J* = 7.0 Hz, 3H). ^{13}C NMR (151 MHz, $CDCl_3$) *δ* 194.72, 158.44, 147.19, 131.70, 128.63, 127.96, 123.32, 120.02, 114.97, 80.18, 63.55, 50.08, 40.87, 39.26, 27.89, 21.51, 14.98 (Fig. S45).

ACQ9: The title compound was prepared according to the same method of ACQ1 from II-9 (612 mg, 1.5 mmol) in 28% as a black solid (163 mg). ESI-HRMS (m/z): $[M]^+$ Calcd. for $[C_{48}H_{48}BF_2N_5O_2]^+$: 775.3864, found: 775.3859.

1.10. Synthesis of ACQ10

I-10: The title compound was prepared according to the same method of I-1 from 9-acetyljulolidine (989 mg, 4.6 mmol) and *p*-propoxybenzaldehyde (836 mg, 5.1 mmol)

in 75% as a yellow solid (1240 mg). ^{1}H NMR (600 MHz, $CDCl_3$) δ 7.72 (d, J = 15.5 Hz, 1H), 7.58 (d, J = 8.7 Hz, 2H), 7.57 (s, 2H), 7.44 (dd, J = 15.5, 7.5 Hz, 1H), 6.91 (d, J = 8.7 Hz, 2H), 3.96 (t, J = 6.6 Hz, 2H), 3.31–3.24 (m, 4H), 2.80 (t, J = 6.3 Hz, 4H), 2.02–1.95 (m, 4H), 1.87–1.79 (m, 2H), 1.05 (t, J = 7.4 Hz, 3H). ^{13}C NMR (151 MHz, $CDCl_3$) δ 187.73, 160.78, 146.83, 141.92, 129.95, 128.41, 128.38, 125.25, 120.17, 120.05, 114.90, 69.75, 50.13, 27.95, 22.67, 21.65, 10.65 (Fig. S46).

II-10: The title compound was prepared according to the same method of II-1 from I-10 (1011 mg, 2.8 mmol) in 74% as a yellow solid (870 mg). ^{1}H NMR (600 MHz, $CDCl_3$) δ 7.38 (d, J = 5.2 Hz, 2H), 7.18 (d, J = 8.6 Hz, 2H), 6.84 (t, J = 5.8 Hz, 2H), 4.81 (dd, J = 12.4, 6.0 Hz, 1H), 4.61 (dd, J = 12.4, 8.7 Hz, 1H), 4.15–4.09 (m, 1H), 3.92–3.84 (m, 2H), 3.31–3.15 (m, 6H), 2.73 (t, J = 6.3 Hz, 4H), 1.98–1.90 (m, 4H), 1.82–1.74 (m, 2H), 1.02 (t, J = 7.4 Hz, 3H). ^{13}C NMR (151 MHz, $CDCl_3$) δ 194.72, 158.64, 147.18, 131.64, 128.60, 127.96, 123.32, 120.02, 114.99, 80.17, 69.61, 50.07, 40.88, 39.26, 27.88, 22.71, 21.51, 10.67 (Fig. S47).

ACQ10: The title compound was prepared according to the same method of ACQ1 from II-10 (633 mg, 1.5 mmol) in 20% as a black solid (120 mg). ^{1}H NMR (600 MHz, pyridine-d_5) δ 8.39 (d, J = 8.3 Hz, 4H), 8.21 (s, 4H), 7.52 (s, 2H), 7.21 (d, J = 8.4 Hz, 4H), 3.87 (t, J = 6.4 Hz, 4H), 3.00 (t, J = 5.4 Hz, 8H), 2.76 (t, J = 6.0 Hz, 8H), 1.76–1.66 (m, 12H), 0.92 (t, J = 7.4 Hz, 6H). ^{13}C NMR (151 MHz, pyridine-d_5) δ 160.73, 156.15, 146.15, 145.77, 140.25, 131.45, 130.55, 127.01, 121.74, 119.30, 117.89, 115.51, 70.11, 50.44, 28.50, 23.31, 22.20, 11.08 (Fig. S48). ESI-HRMS (m/z): $[M]^+$ Calcd. for $[C_{50}H_{52}BF_2N_5O_2]^+$: 803.4177, found: 803.4172.

1.11. Synthesis of ACQ11

I-11: The title compound was prepared according to the same method of I-1 from 9-acetyljulolidine (989 mg, 4.6 mmol) and *p*-isopropoxybenzaldehyde (836 mg, 5.1 mmol) in 73% as a yellow solid (1210 mg). ^{1}H NMR (600 MHz, $CDCl_3$) δ 7.72 (d, J = 15.5 Hz, 1H), 7.57 (d, J = 9.9 Hz, 4H), 7.47–7.41 (m, 1H), 6.89 (d, J = 8.7 Hz, 2H), 4.60 (hept, J = 6.1 Hz, 1H), 3.27 (dd, J = 13.3, 7.5 Hz, 4H), 2.80 (t, J = 6.3 Hz, 4H), 2.03–1.92 (m, 4H), 1.36 (d, J = 6.1 Hz, 6H). ^{13}C NMR (151 MHz, $CDCl_3$) δ 187.74, 159.59, 146.82, 141.92, 129.99, 128.41, 128.26, 125.26, 120.17, 120.03, 116.00, 70.10, 50.13, 27.95, 22.15, 21.65 (Fig. S49).

II-11: The title compound was prepared according to the same method of II-1 from I-11 (1011 mg, 2.8 mmol) in 78% as a yellow solid (920 mg). ^{1}H NMR (600

MHz, $CDCl_3$) δ 7.38 (s, 2H), 7.17 (d, J = 8.6 Hz, 2H), 6.82 (d, J = 8.6 Hz, 2H), 4.81 (dd, J = 12.4, 6.1 Hz, 1H), 4.61 (dd, J = 12.4, 8.7 Hz, 1H), 4.49 (hept, J = 6.1 Hz, 1H), 4.16–4.08 (m, 1H), 3.31–3.15 (m, 6H), 2.73 (t, J = 6.3 Hz, 4H), 2.00–1.91 (m, 4H), 1.31 (d, J = 6.1 Hz, 6H). ^{13}C NMR (151 MHz, $CDCl_3$) δ 194.75, 157.41, 147.18, 131.61, 128.65, 127.96, 123.33, 120.02, 116.21, 80.14, 69.97, 50.07, 40.90, 39.23, 27.89, 22.21, 21.51 (Fig. S50).

ACQ11: The title compound was prepared according to the same method of ACQ1 from II-11 (633 mg, 1.5 mmol) in 27% as a black solid (162 mg). ^{1}H NMR (600 MHz, pyridine-d_5) δ 8.41 (d, J = 8.1 Hz, 4H), 8.24 (s, 4H), 7.56 (s, 2H), 7.23 (d, J = 8.8 Hz, 4H), 4.62 (dt, J = 12.0, 6.1 Hz, 2H), 3.03 (t, J = 5.4 Hz, 8H), 2.79 (t, J = 6.1 Hz, 8H), 1.82–1.72 (m, 8H), 1.29 (d, J = 5.9 Hz, 12H). ^{13}C NMR (151 MHz, pyridine-d_5) δ 158.20, 154.84, 144.82, 144.45, 138.95, 130.23, 129.21, 125.52, 120.41, 117.98, 116.52, 115.19, 68.99, 49.11, 27.17, 21.15, 20.87 (Fig. S51). ESI-HRMS (m/z): $[M]^+$ Calcd. for $[C_{50}H_{52}BF_2N_5O_2]^+$: 803.4177, found: 803.4182.

1.12. Synthesis of ACQ12

I-12: The title compound was prepared according to the same method of I-1 from 9-acetyljulolidine (989 mg, 4.6 mmol) and *p*-(*tert*-butoxy)benzaldehyde (908 mg, 5.1 mmol) in 59% as a yellow solid (1020 mg). ^{1}H NMR (600 MHz, $CDCl_3$) δ 7.73 (d, J = 15.5 Hz, 1H), 7.56 (d, J = 9.5 Hz, 4H), 7.50–7.44 (m, 1H), 7.01 (d, J = 8.0 Hz, 2H), 3.28 (t, J = 5.5 Hz, 4H), 2.80 (t, J = 6.1 Hz, 4H), 1.98 (dd, J = 11.4, 5.7 Hz, 4H), 1.39 (s, 9H). ^{13}C NMR (151 MHz, $CDCl_3$) δ 187.65, 157.41, 146.90, 141.72, 130.77, 129.19, 128.46, 125.16, 123.99, 121.13, 120.19, 79.33, 50.14, 29.06, 27.96, 21.64 (Fig. S52).

II-12: The title compound was prepared according to the same method of II-1 from I-12 (1050 mg, 2.8 mmol) in 59% as a yellow solid (720 mg). ^{1}H NMR (600 MHz, $CDCl_3$) δ 7.39 (s, 2H), 7.16 (d, J = 7.6 Hz, 2H), 6.92 (d, J = 7.6 Hz, 2H), 4.82 (dd, J = 12.4, 6.0 Hz, 1H), 4.66–4.57 (m, 1H), 4.15 (p, J = 7.0 Hz, 1H), 3.32–3.17 (m, 6H), 2.73 (t, J = 6.0 Hz, 4H), 2.01–1.89 (m, 4H), 1.32 (s, 9H). ^{13}C NMR (151 MHz, $CDCl_3$) δ 194.71, 154.95, 147.20, 134.57, 128.08, 127.96, 124.46, 123.34, 120.03, 80.04, 78.67, 50.08, 40.81, 39.31, 28.99, 27.89, 21.52 (Fig. S53).

ACQ12: The title compound was prepared according to the same method of ACQ1 from II-12 (654 mg, 1.5 mmol) in 22% as a black solid (137 mg). ^{1}H NMR (600 MHz, pyridine-d_5) δ 8.37 (d, J = 7.8 Hz, 4H), 8.24 (s, 4H), 7.58–7.56 (m, 2H),

7.34 (d, J = 7.8 Hz, 4H), 3.04 (s, 8H), 2.79 (t, J = 5.5 Hz, 8H), 1.77 (d, J = 5.2 Hz, 8H), 1.40 (s, 18H). ^{13}C NMR (151 MHz, pyridine-d_5) δ 157.34, 156.14, 146.25, 145.84, 140.03, 130.91, 130.60, 129.25, 121.80, 119.20, 118.41, 79.33, 50.45, 29.34, 28.48, 22.17 (Fig. S54). ESI-HRMS (m/z): $[M]^+$ Calcd. for $[C_{52}H_{56}BF_2N_5O_2]^+$: 831.4490, found: 831.4488.

1.13. Synthesis of ACQ13

I-13: The title compound was prepared according to the same method of I-1 from 9-acetyljulolidine (989 mg, 4.6 mmol) and *p*-amoxybenzaldehyde (979 mg, 5.1 mmol) in 67% as a yellow solid (1200 mg). ^{1}H NMR (600 MHz, $CDCl_3$) δ 7.72 (d, J = 15.5 Hz, 1H), 7.58 (d, J = 8.7 Hz, 2H), 7.57 (s, 2H), 7.46–7.42 (m, 1H), 6.91 (d, J = 8.7 Hz, 2H), 3.99 (t, J = 6.6 Hz, 2H), 3.27 (dd, J = 12.2, 6.5 Hz, 4H), 2.80 (t, J = 6.3 Hz, 4H), 2.01–1.94 (m, 4H), 1.84–1.77 (m, 2H), 1.49–1.36 (m, 4H), 0.94 (t, J = 7.2 Hz, 3H). ^{13}C NMR (151 MHz, $CDCl_3$) δ 198.64, 187.74, 160.80, 146.83, 141.94, 129.95, 128.41, 128.36, 125.26, 120.17, 120.05, 114.90, 68.26, 50.13, 29.04, 28.32, 27.96, 22.60, 21.65, 14.16 (Fig. S55).

II-13: The title compound was prepared according to the same method of II-1 from I-13 (1089 mg, 2.8 mmol) in 64% as a yellow solid (810 mg). ^{1}H NMR (600 MHz, $CDCl_3$) δ 7.38 (s, 2H), 7.18 (d, J = 8.0 Hz, 2H), 6.83 (d, J = 8.0 Hz, 2H), 4.81 (dd, J = 12.3, 5.9 Hz, 1H), 4.65–4.57 (m, 1H), 4.16–4.09 (m, 1H), 3.91 (t, J = 6.4 Hz, 2H), 3.30–3.16 (m, 6H), 2.73 (t, J = 6.0 Hz, 4H), 1.99–1.90 (m, 4H), 1.80–1.72 (m, 2H), 1.45–1.33 (m, 4H), 0.92 (t, J = 6.9 Hz, 3H). ^{13}C NMR (151 MHz, $CDCl_3$) δ 194.73, 158.66, 147.19, 131.63, 128.60, 127.97, 123.34, 120.02, 114.99, 80.18, 68.10, 50.08, 40.89, 39.27, 29.10, 28.35, 27.89, 22.59, 21.52, 14.16 (Fig. S56).

ACQ13: The title compound was prepared according to the same method of ACQ1 from II-13 (675 mg, 1.5 mmol) in 21% as a black solid (137 mg). ^{1}H NMR (600 MHz, pyridine-d_5) δ 8.44 (d, J = 8.0 Hz, 4H), 8.25 (s, 4H), 7.57 (s, 2H), 7.28 (d, J = 8.1 Hz, 4H), 3.99 (t, J = 6.4 Hz, 4H), 3.04 (s, 8H), 2.80 (s, 8H), 1.75 (dt, J = 14.4, 6.1 Hz, 12H), 1.38 (dt, J = 12.6, 6.3 Hz, 4H), 1.28 (dd, J = 14.2, 7.0 Hz, 4H), 0.88 (t, J = 7.2 Hz, 6H). ^{13}C NMR (151 MHz, pyridine-d_5) δ 160.78, 156.16, 146.16, 145.78, 140.26, 131.48, 130.55, 127.02, 121.75, 119.31, 117.89, 115.53, 68.67, 50.44, 29.68, 28.86, 28.50, 23.13, 22.20, 14.61 (Fig. S57). ESI-HRMS (m/z): $[M]^+$ Calcd. for $[C_{54}H_{60}BF_2N_5O_2]^+$: 859.4803, found: 859.4796.

1.14. Synthesis of ACQ14

I-14: The title compound was prepared according to the same method of I-1 from 9-acetyljulolidine (989 mg, 4.6 mmol) and *p*-(2-methoxyethoxy)benzaldehyde (918 mg, 5.11 mmol) in 76% as a yellow solid (1320 mg). ^{1}H NMR (600 MHz, $CDCl_3$) δ 7.72 (d, *J* = 15.4 Hz, 1H), 7.62–7.52 (m, 4H), 7.45 (d, *J* = 15.7 Hz, 1H), 6.95 (d, *J* = 7.7 Hz, 2H), 4.16 (s, 2H), 3.77 (s, 2H), 3.46 (s, 3H), 3.28 (s, 4H), 2.76 (d, *J* = 42.0 Hz, 4H), 1.97 (s, 4H). ^{13}C NMR (151 MHz, $CDCl_3$) δ 187.69, 160.35, 146.86, 141.78, 129.92, 128.82, 128.43, 125.21, 120.33, 120.18, 115.02, 71.08, 67.49, 59.41, 50.13, 27.95, 21.64 (Fig. S58).

II-14: The title compound was prepared according to the same method of II-1 from I-14 (1055 mg, 2.8 mmol) in 87% as a yellow solid (1060 mg). ^{1}H NMR (600 MHz, $CDCl_3$) δ 7.38 (s, 2H), 7.18 (d, *J* = 7.7 Hz, 2H), 6.87 (d, *J* = 7.7 Hz, 2H), 4.81 (dd, *J* = 12.3, 5.9 Hz, 1H), 4.63–4.56 (m, 1H), 4.16–4.09 (m, 1H), 4.08 (d, *J* = 3.6 Hz, 2H), 3.73 (s, 2H), 3.44 (s, 3H), 3.28–3.18 (m, 6H), 2.73 (t, *J* = 6.1 Hz, 4H), 1.98–1.89 (m, 4H). ^{13}C NMR (151 MHz, $CDCl_3$) δ 194.68, 158.26, 147.19, 132.11, 128.64, 127.95, 123.30, 120.01, 115.12, 80.14, 71.13, 67.37, 59.33, 50.06, 40.81, 39.25, 27.88, 21.50 (Fig. S59).

ACQ14: The title compound was prepared according to the same method of ACQ1 from II-14 (657 mg, 1.5 mmol) in 34% as a black solid (210 mg). ^{1}H NMR (600 MHz, pyridine-d_5) δ 8.39 (d, *J* = 6.4 Hz, 4H), 8.24 (s, 4H), 7.54 (s, 2H), 7.26 (d, *J* = 6.7 Hz, 4H), 4.21 (s, 4H), 3.74 (s, 4H), 3.36 (d, *J* = 3.7 Hz, 6H), 3.04 (s, 8H), 2.80 (d, *J* = 4.4 Hz, 8H), 1.77 (d, *J* = 4.3 Hz, 8H). ^{13}C NMR (151 MHz, pyridine-d_5) δ 160.48, 156.13, 146.17, 145.75, 140.10, 131.44, 130.55, 127.24, 121.75, 119.28, 117.95, 115.55, 71.66, 68.20, 59.20, 50.44, 28.49, 22.19 (Fig. S60). ESI-HRMS (m/z): $[M]^+$ Calcd. for $[C_{50}H_{52}BF_2N_5O_4]^+$: 835.4075, found: 835.4071.

2. Supplementary figures and tables

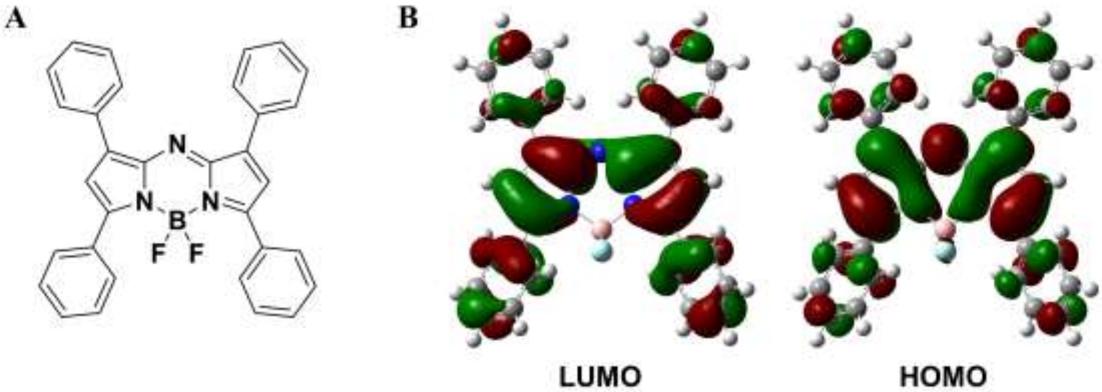

Figure S1 Chemical structure (A) and frontier molecular orbitals (B) of tetraphenyl aza-BODIPY at the B3LYP/6-31+G (d, p) level of theory by using the Gaussian 09 program.

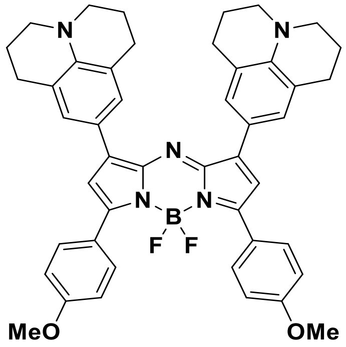

NJ1060

Figure S2 The chemical structure of NJ1060.

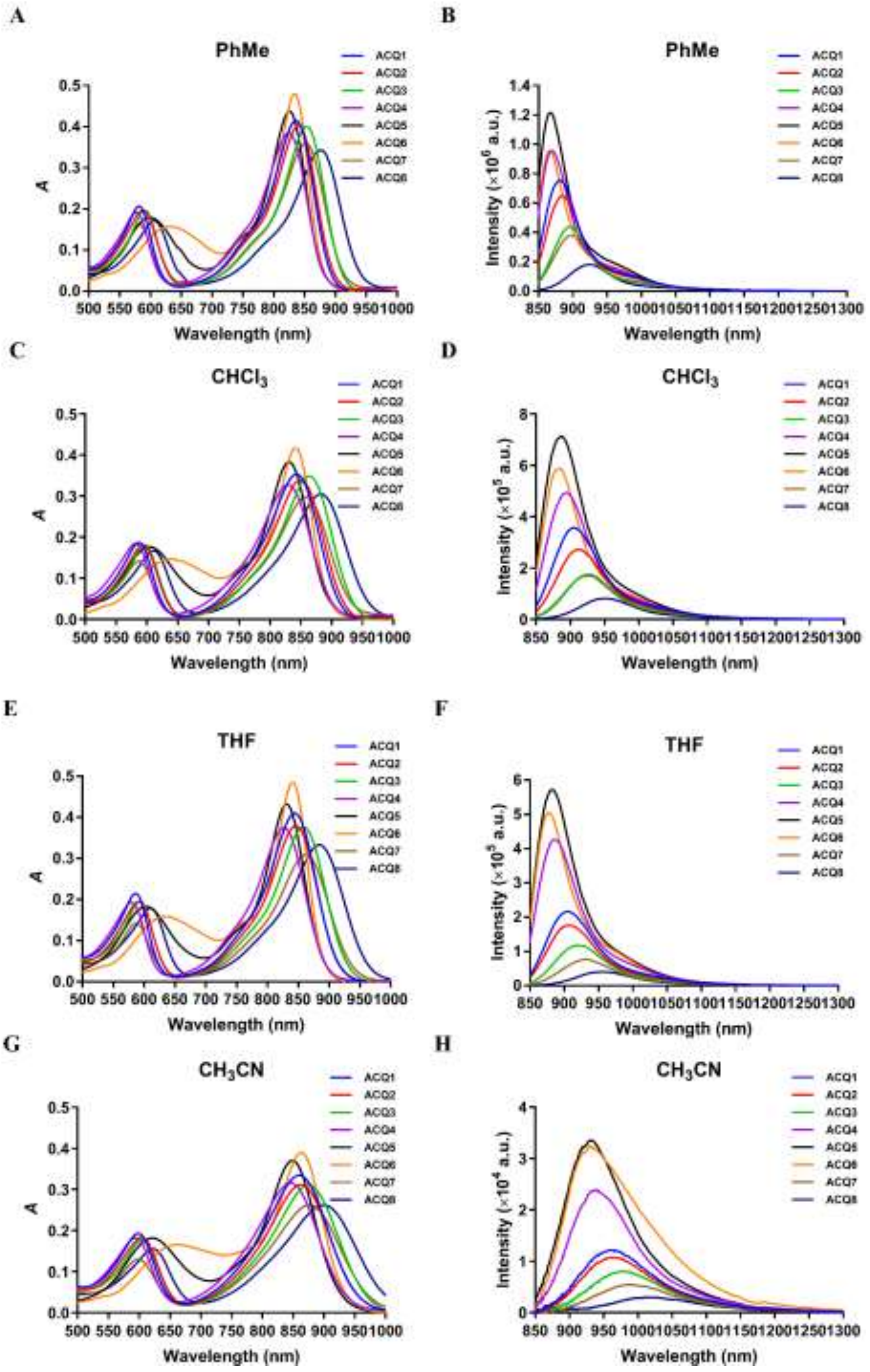

Figure S3 Absorption spectra of 3,5-julolidineyl aza-BODIPYs (5 μmol/L) containing 1,7-substituents with different steric hindrance and electrical property in PhMe (A), $CHCl_3$ (C), THF (E), and CH_3CN (G) system, respectively; Fluorescence spectra of 3,5-julolidineyl aza-BODIPYs (5 μmol/L) containing 1,7-substituents with different steric hindrance and electrical property under excitation of 808 nm laser in PhMe (B), $CHCl_3$ (D), THF (F) and CH_3CN (H) system, respectively.

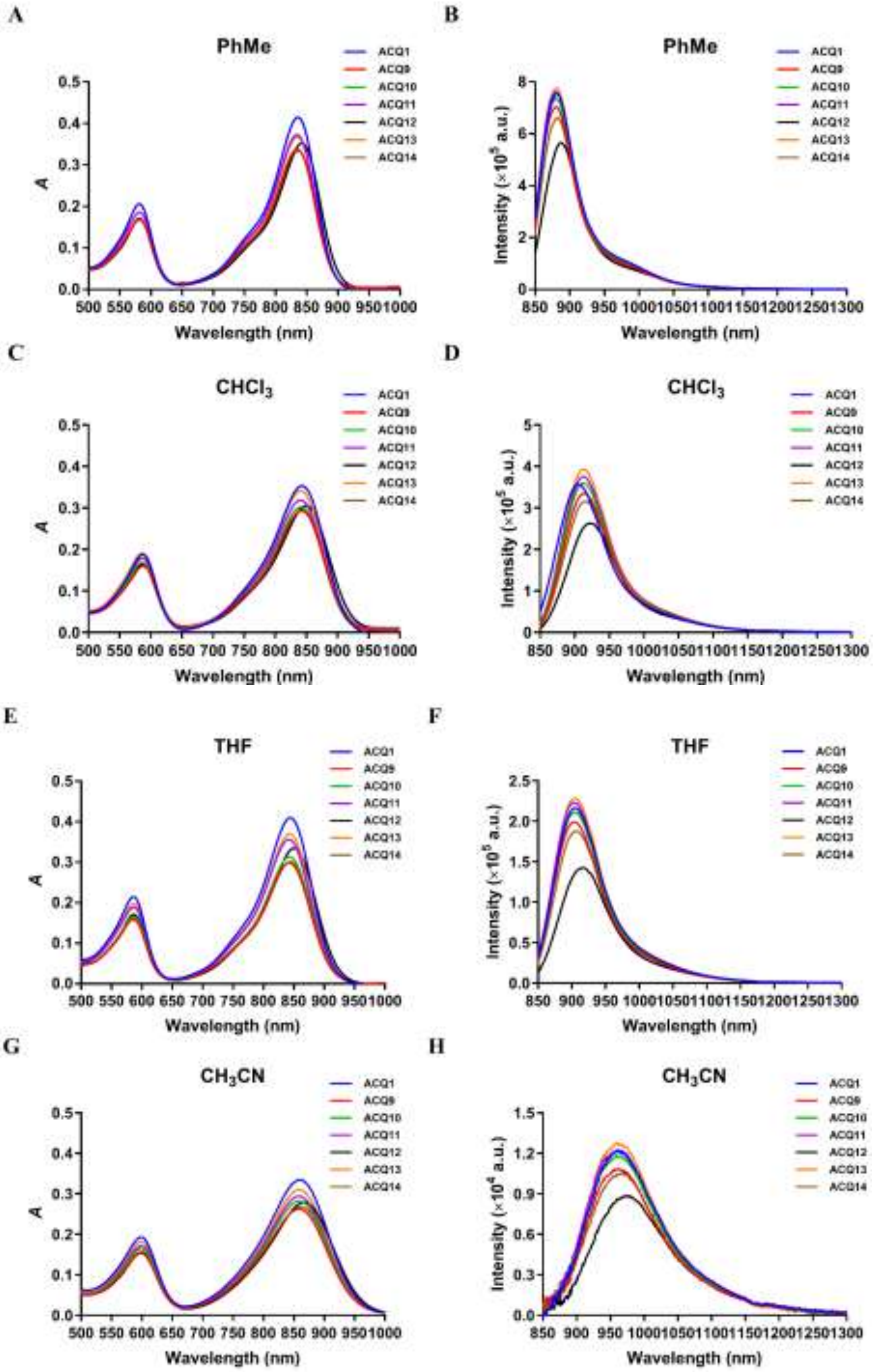

Figure S4 Absorption spectra of 3,5-julolidineyl aza-BODIPYs (5 µmol/L) containing different side chains in 1,7-substituents in PhMe (A), $CHCl_3$ (C), THF (E), and CH_3CN (G) system, respectively; Fluorescence spectra of 3,5-julolidineyl aza-BODIPYs (5 µmol/L) containing different side chains in 1,7-substituents under excitation of 808 nm laser in PhMe (B), $CHCl_3$ (D), THF (F) and CH_3CN (H) system, respectively.

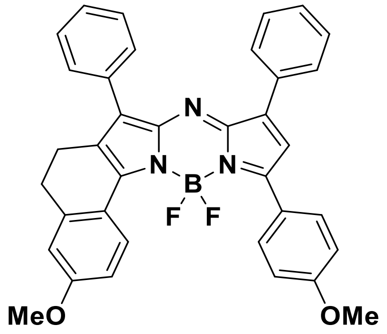

P2

λ_{ab} **= 708 nm**

λ_{em} **= 732 nm**

ε **= 96200 M^{-1} cm^{-1}**

Φ **= 0.38**

Figure S5 The chemical structure and photophysical properties ($CHCl_3$) of P2[1].

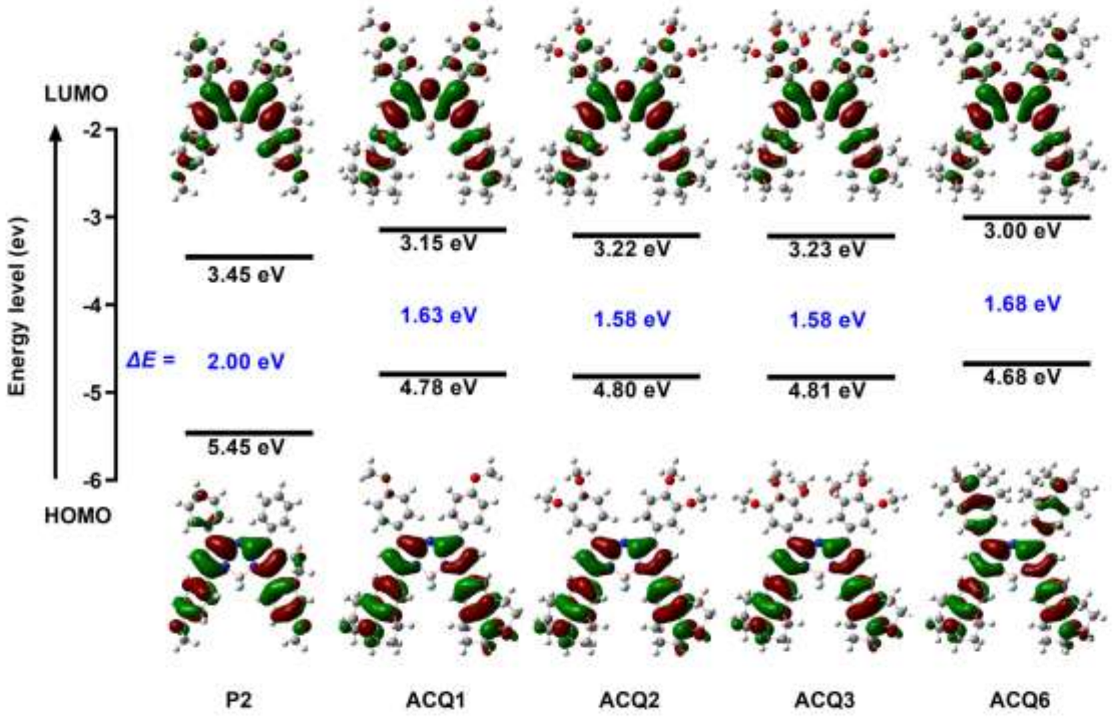

Figure S6 Frontier molecular orbitals of **P2** and selected 3,5-julolidineyl aza-BODIPYs at the B3LYP/6-31+G (d, p) level of theory by using the Gaussian 09 program.

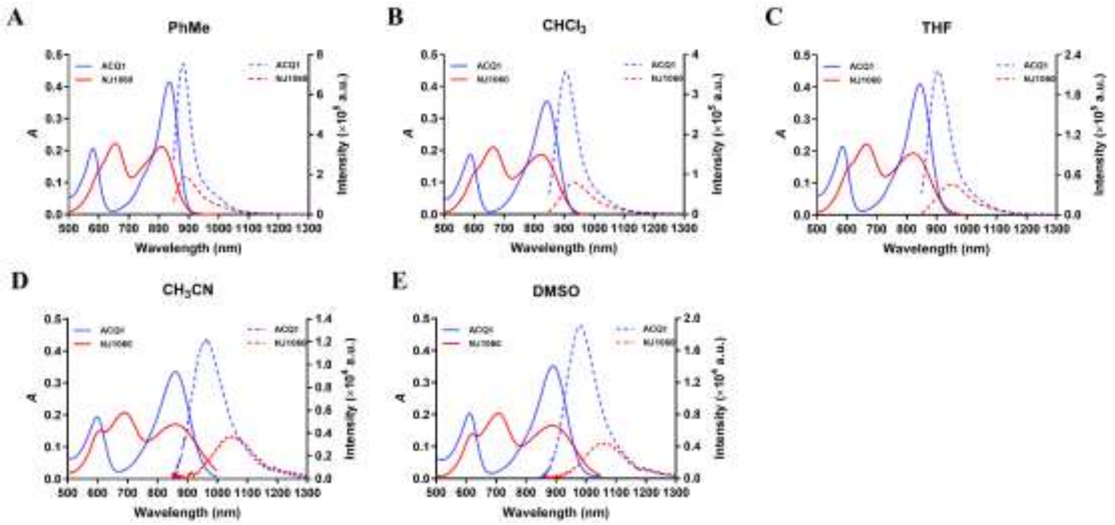

Figure S7 Absorption (solid line) and fluorescence (dotted line) spectra of **ACQ1** (5 μmol/L) and **NJ1060** (5 μmol/L) in PhMe (A), $CHCl_3$ (B), THF (C), CH_3CN (D), and DMSO (E). The emission spectra were excited by an 808 nm laser with the same power.

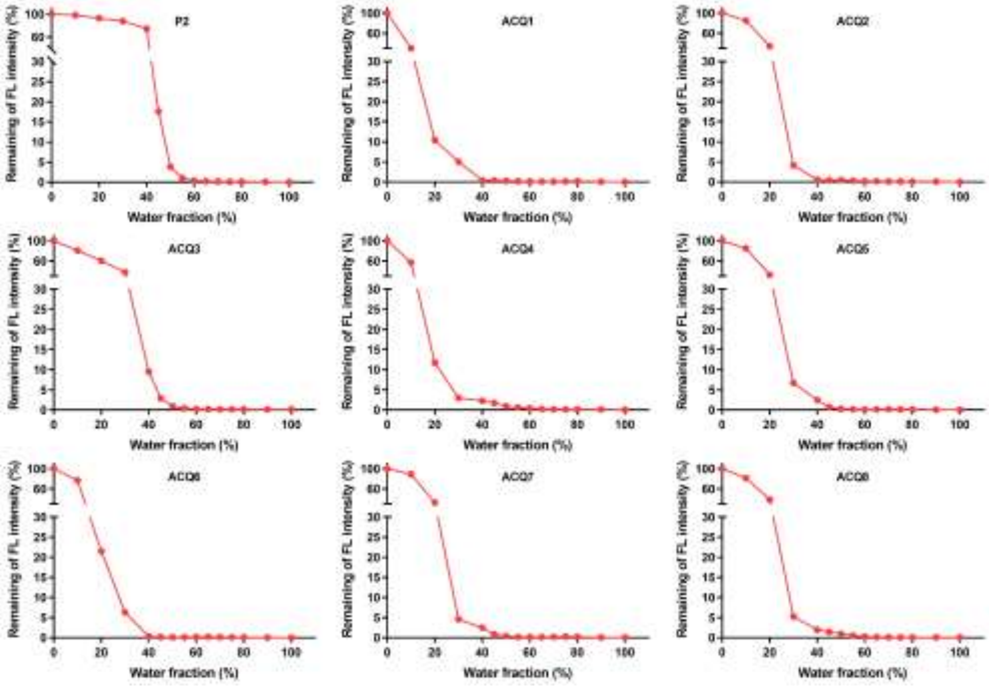

Figure S8 Water quenching fluorescence curves of **P2** and 3,5-julolidineyl aza-BODIPYs in DMSO/water binary systems.

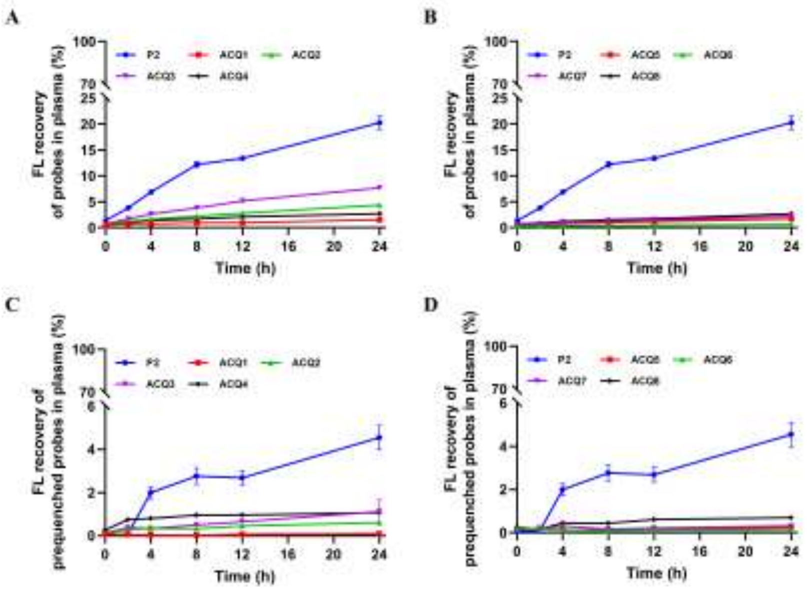

Figure S9 Fluorescence re-illumination (%) of 3,5-julolidineyl aza-BODIPYs containing 1,7-substituents with different steric hindrance and electrical properties (A–B) and prequenched 3,5-julolidineyl aza-BODIPYs containing 1,7-substituents with different steric hindrance and electrical property (C–D) in plasma after different incubation time with P2 as reference (*n* = 3; mean ± SD).

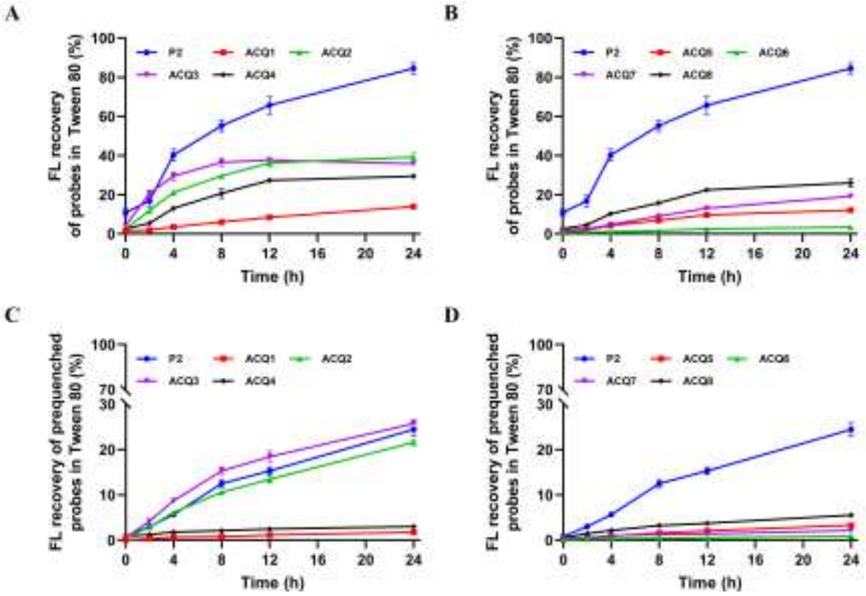

Figure S10 Fluorescence re-illumination (%) of 3,5-julolidineyl aza-BODIPYs containing 1,7-substituents with different steric hindrance and electrical properties (A–B) and prequenched 3,5-julolidineyl aza-BODIPYs containing 1,7-substituents with different steric hindrance and electrical property (C–D) in 1% Tween 80 after different incubation time with P2 as reference (*n* = 3; mean ± SD).

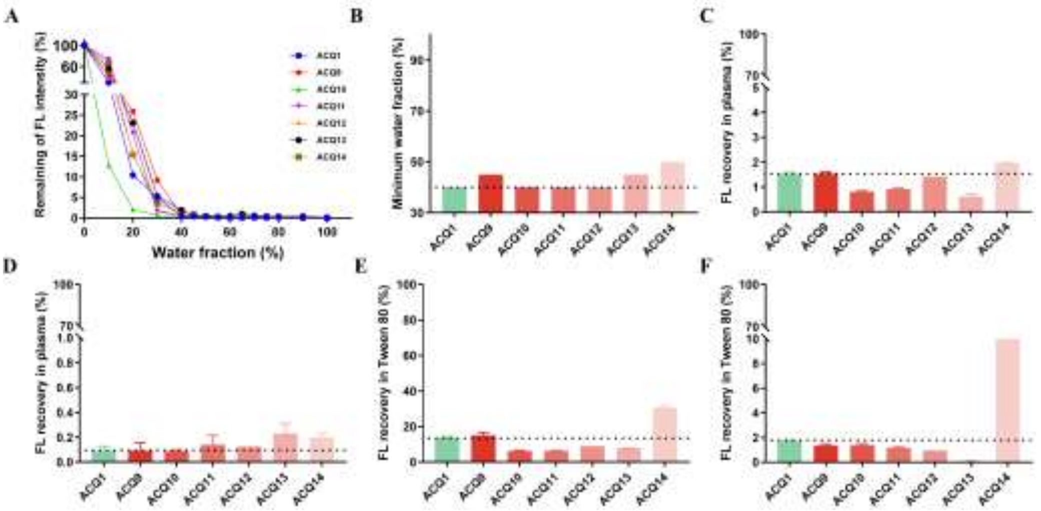

Figure S11 ACQ properties of 3,5-julolidinyl aza-BODIPYs with different side chains. Water quenching fluorescence curves (A) and minimum water fraction (B) in DMSO/water binary systems; Fluorescence re-illumination (%) of probes (C) and prequenched probes (D) in plasma after 24-hour incubation (n = 3; mean ± SD); Fluorescence re-illumination (%) of probes (E) and prequenched probes (F) in 1% Tween 80 after 24-hour incubation (n = 3; mean ± SD). Each dotted line in (B)–(F) represents the level of ACQ1.

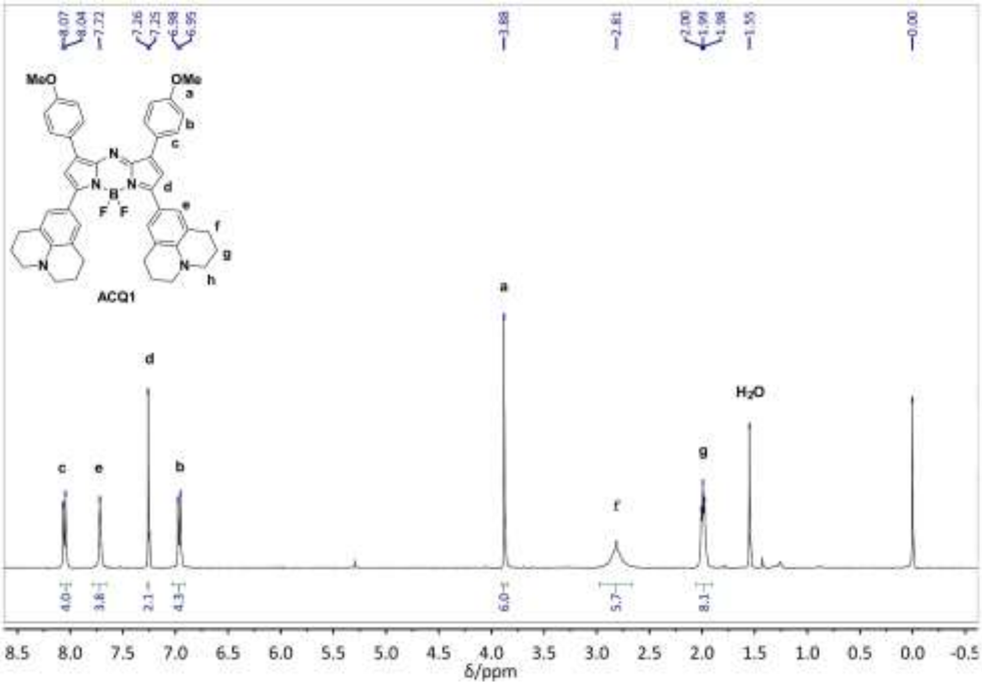

Figure S12 The ^{1}H NMR spectrum of ACQ1 in $CDCl_3$. The signal peak of f in julolidinyl moiety was low and wide with decreasing integral. Moreover, the peak for h disappeared in the ^{1}H NMR spectrum.

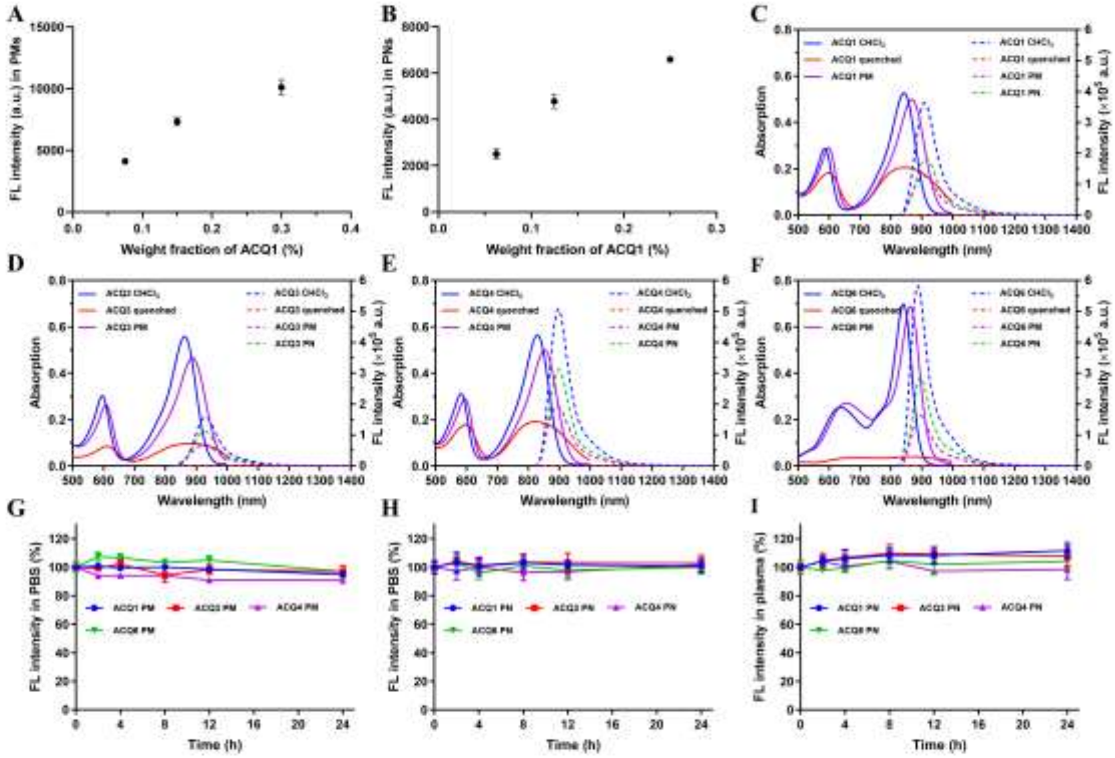

Figure S13 Fluorescence intensity of PMs (A) and PNs (B) as a function of weight fraction of ACQ1 in the above-1000 nm region after 10-fold dilution. The absorption (solid line)/emission (dotted line) spectra of probes in $CHCl_3$, PMs, PNs, and aqueous dispersion (quenched state) at the same concentration (C: ACQ1; D: ACQ3; E: ACQ4; F: ACQ6). Fluorescence stability of labeled PMs in PBS (G) and labeled PNs in PBS (H) and plasma (I) (n = 3; mean ± SD).

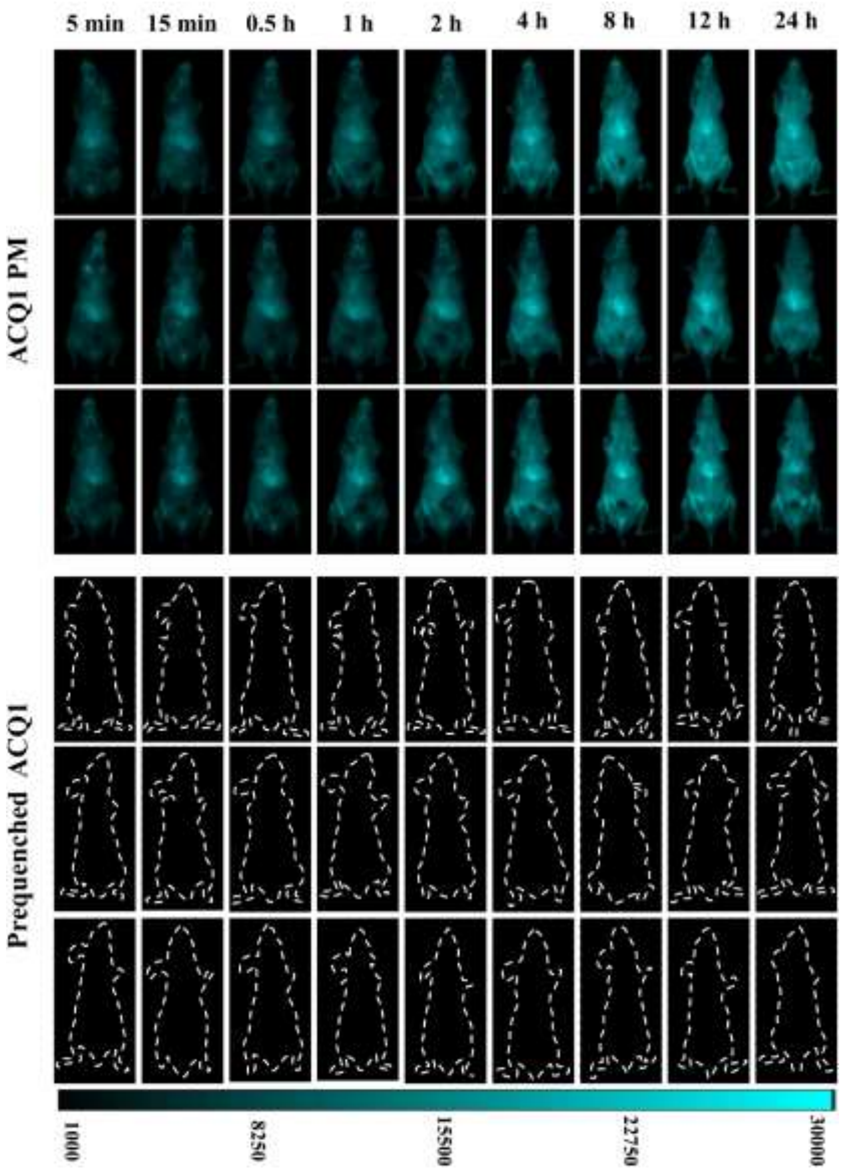

Figure S14 Live images of ACQ1-labeled PMs and quenched dye dispersions after *i.v.* injection in mice under fasted state (n = 3).

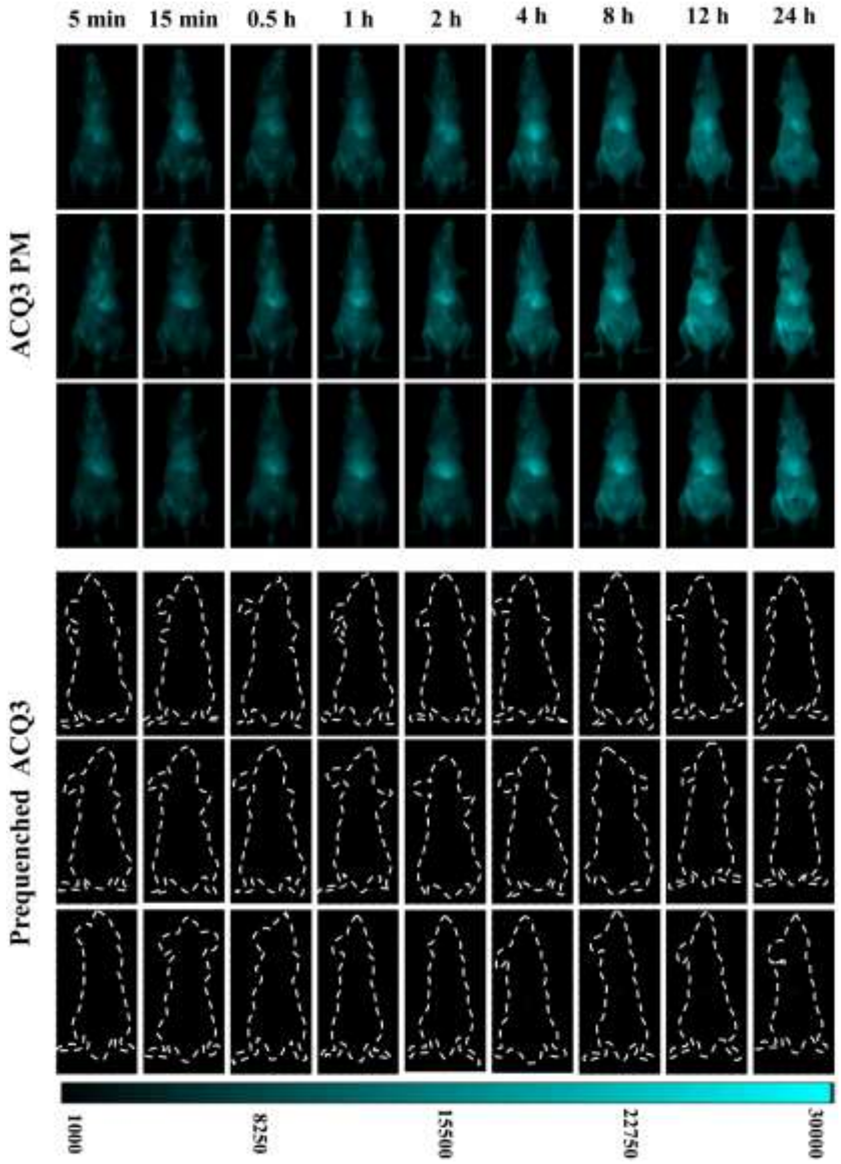

Figure S15 Live images of ACQ3-labeled PMs and quenched dye dispersions after *i.v.* injection in mice under fasted state (n = 3).

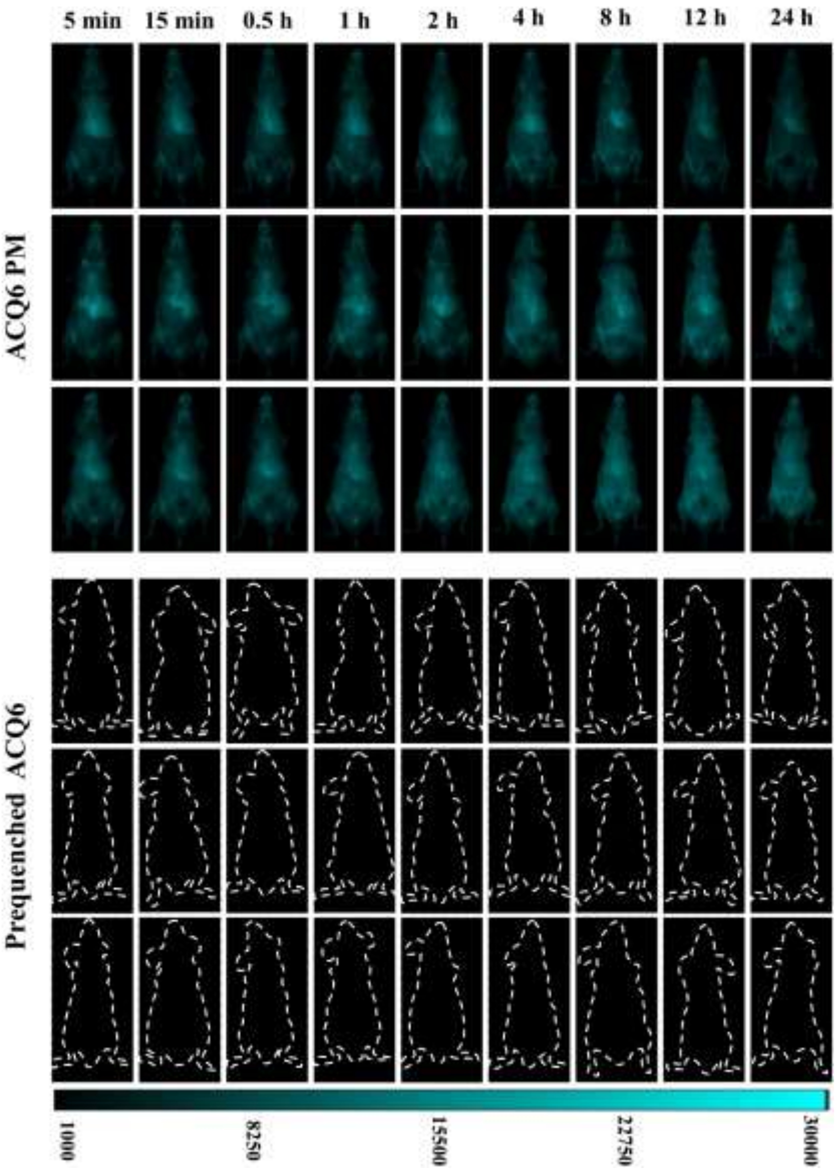

Figure S16 Live images of ACQ6-labeled PMs and quenched dye dispersions after *i.v.* injection in mice under fasted state (n = 3).

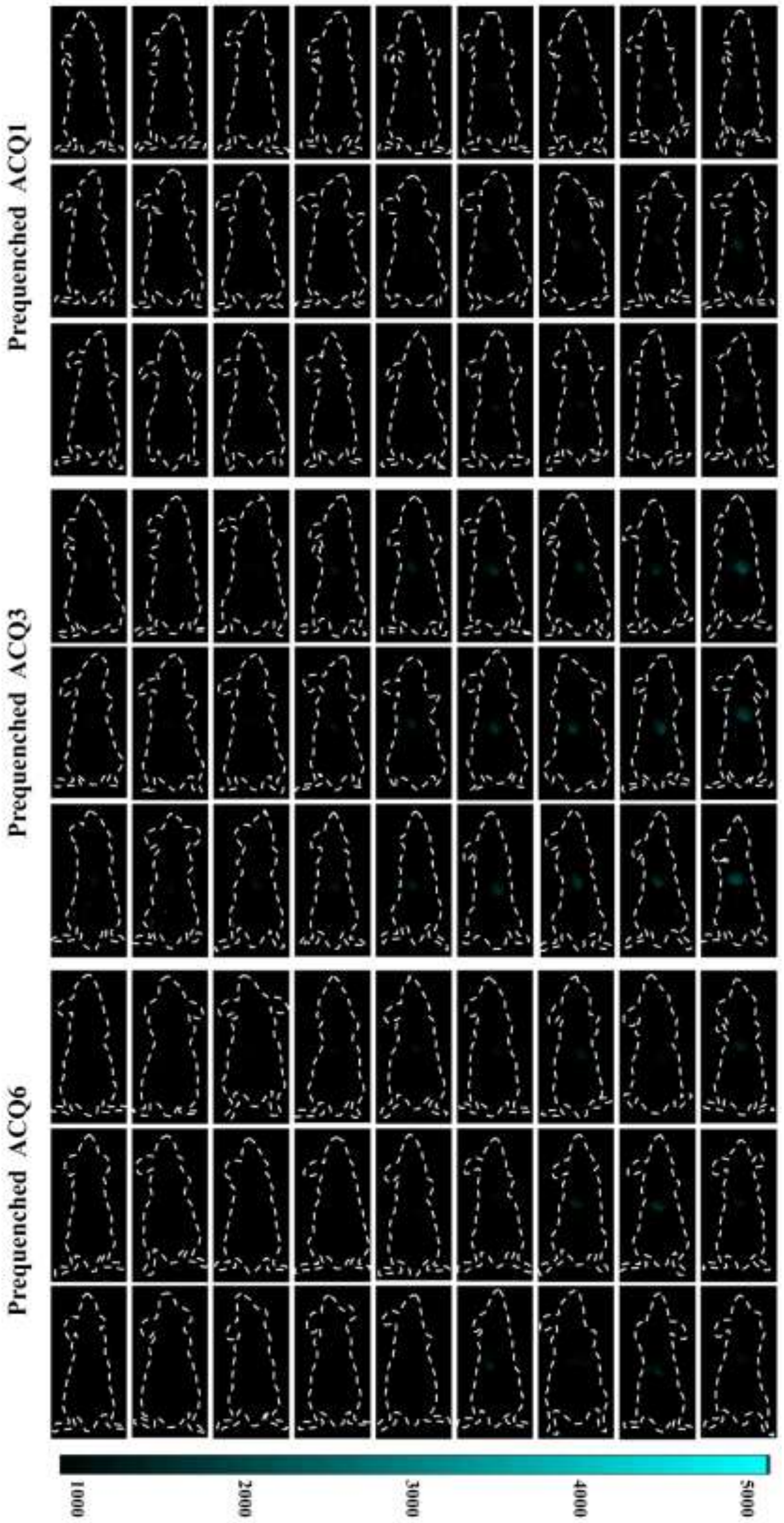

Figure S17 Live images of quenched dye dispersions (ACQ1, ACQ3, and ACQ6) in a more subtle calibration bar after *i.v.* injection in mice under fasted state (n = 3).

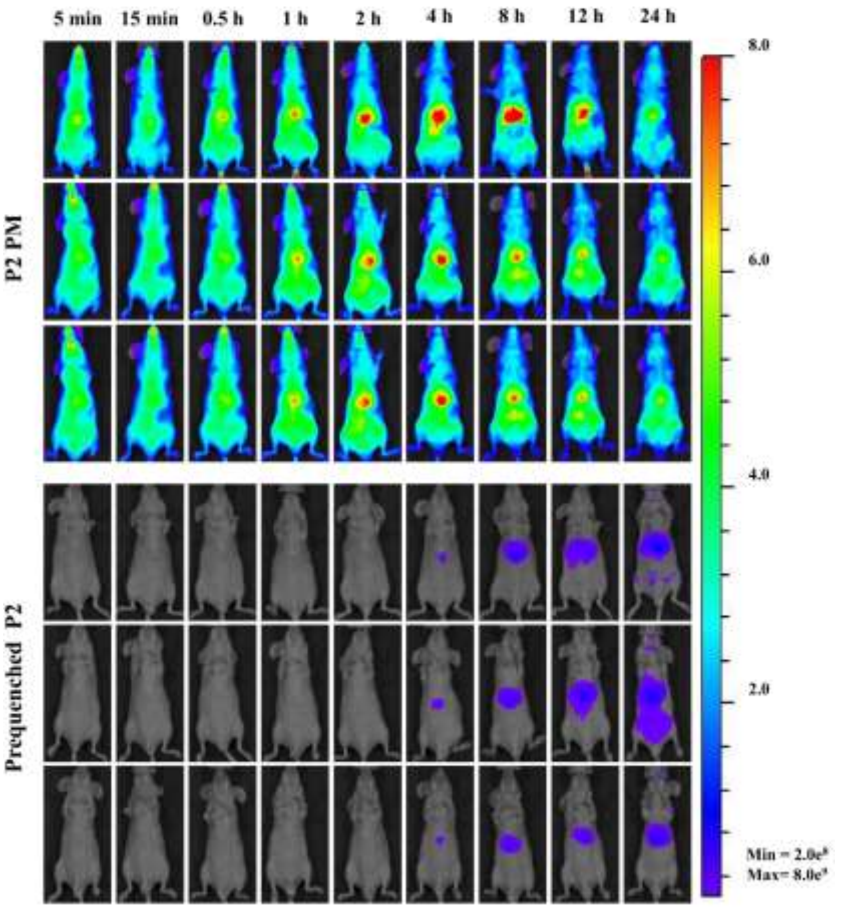

Figure S18 Live images of P2-labeled PMs and quenched dye dispersions after *i.v.* injection in mice under fasted state (*n* = 3).

Table S1 Photophysical parameters of 3,5-julolidineyl aza-BODIPYs in PhMe, $CHCl_3$, THF, and CH_3CN system respectively.

Compound	λ_{ab} (nm)				λ_{em} (nm)				Stokes shift (nm)			
	PhMe	$CHCl_3$	THF	CH_3CN	PhMe	$CHCl_3$	THF	CH_3CN	PhMe	$CHCl_3$	THF	CH_3CN
ACQ1	836	843	845	860	882	905	905	962	46	62	60	102
ACQ2	840	850	846	863	885	912	906	962	45	62	60	99
ACQ3	853	864	859	875	894	929	919	978	41	65	60	103
ACQ4	824	830	826	846	870	894	886	937	46	64	60	91
ACQ5	826	831	831	849	868	887	882	930	42	56	51	81
ACQ6	834	841	841	863	866	882	877	930	32	41	36	67
ACQ7	852	858	862	877	897	929	932	992	45	71	70	115
ACQ8	876	882	884	900	922	952	957	1012	46	70	73	112
ACQ9	835	842	842	858	880	903	902	962	45	61	60	104
ACQ10	835	841	843	859	880	903	905	959	45	62	62	100
ACQ11	834	841	842	859	880	903	902	961	46	62	60	102
ACQ12	843	849	851	867	887	914	917	975	44	65	66	108
ACQ13	835	841	843	859	880	902	904	961	45	61	61	102
ACQ14	837	845	844	862	882	907	907	969	45	62	63	107
NJ1060	811	827	823	864	891	934	946	1050	80	107	123	186

Table S2 Photophysical parameters of labeled PMs and PNs

Probe	PMs				PNs
	λ_{ab} (nm)	λ_{em} (nm)	Φ (850–1400 nm)	Φ (1000–1400 nm)	λ_{em} (nm)
ACQ1	868	908	1.67%	0.23%	912
ACQ3	888	930	1.54%	0.29%	930
ACQ4	852	894	2.01%	0.22%	900
ACQ6	863	894	1.78%	0.21%	896

Table S3 Particle size, PDI, and zeta potential of fluorescently labeled PMs

Probe	Size (nm)	PDI	Zeta potential (mV)	EE (%)
ACQ1	22.52±0.5002	0.157±0.005	-3.41±0.580	98.7
ACQ3	21.90±0.5030	0.156±0.015	-5.17±0.350	81.6
ACQ4	22.13±0.0902	0.159±0.009	-9.99±1.18	98.1
ACQ6	22.38±0.7318	0.166±0.013	-6.84±2.28	99.5

Table S4 Particle size, PDI, and zeta potential of fluorescently labeled PNs

Probe	Size (nm)	PDI	Zeta potential (mV)	EE (%)
ACQ1	202.7±4.451	0.104±0.030	-0.328±0.200	94.2
ACQ3	218.4±1.222	0.058±0.032	-1.17±0.0451	76.0
ACQ4	214.9±2.95	0.053±0.020	-1.15±0.0173	98.9
ACQ6	233.5±5.500	0.044±0.036	-2.36±0.0231	97.6

3. Copies of spectra of selected compounds

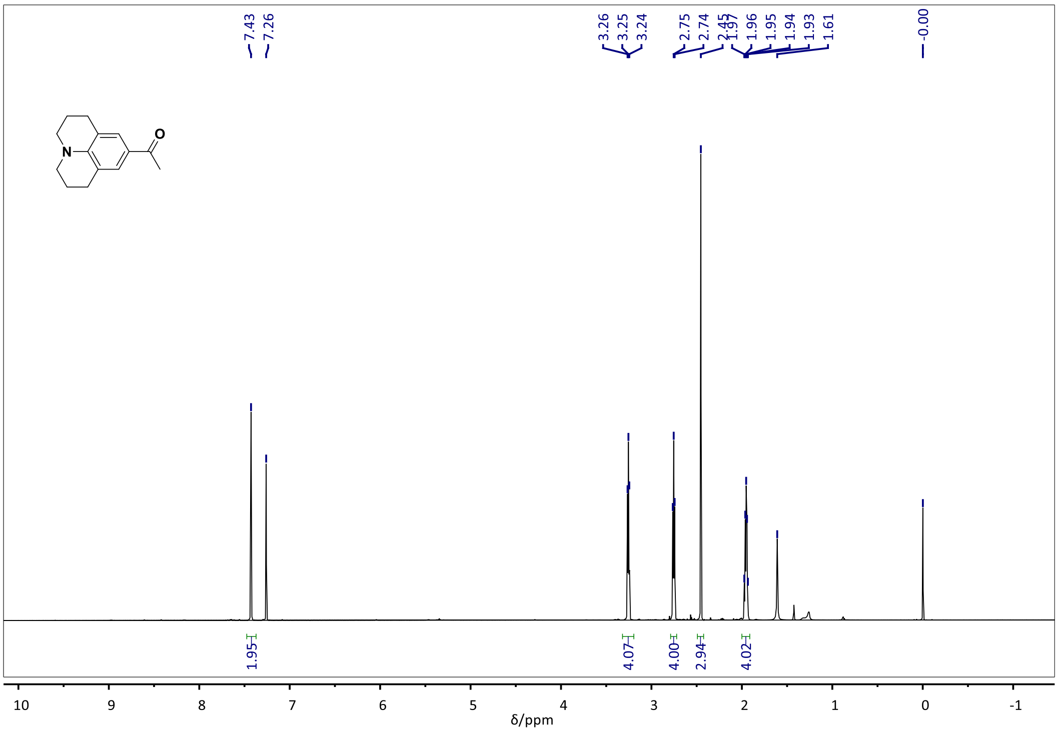

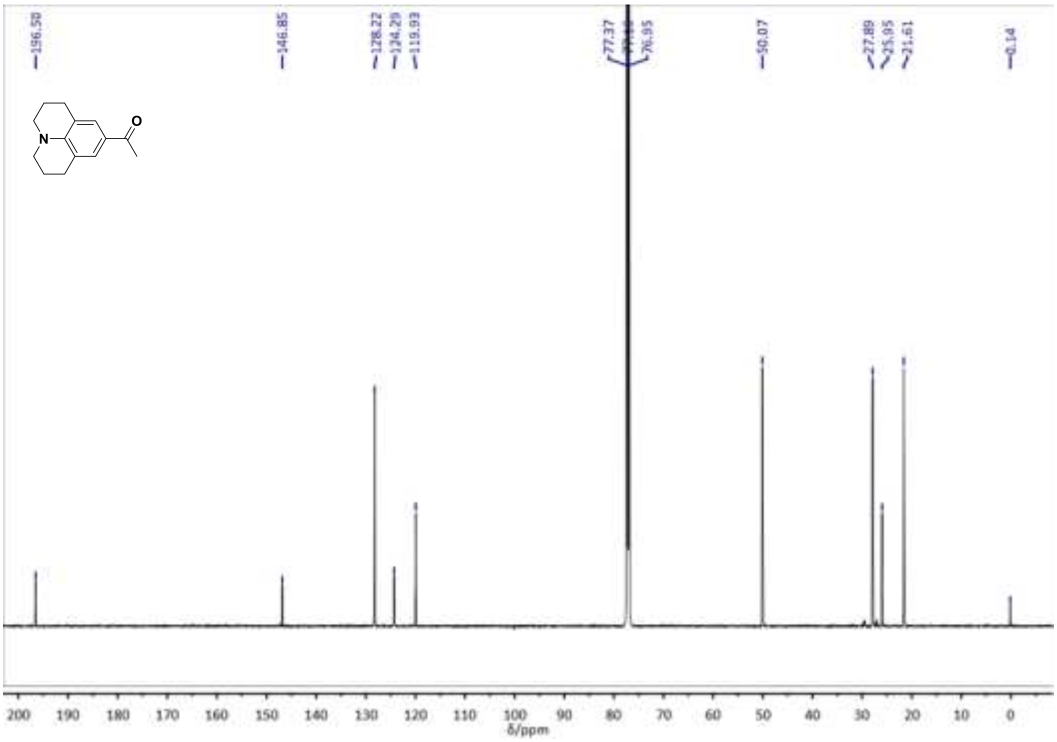

Figure S19 ^{1}H NMR (600 MHz, $CDCl_3$) and ^{13}C NMR (151 MHz, $CDCl_3$) spectra of 9-acetyljulolidine.

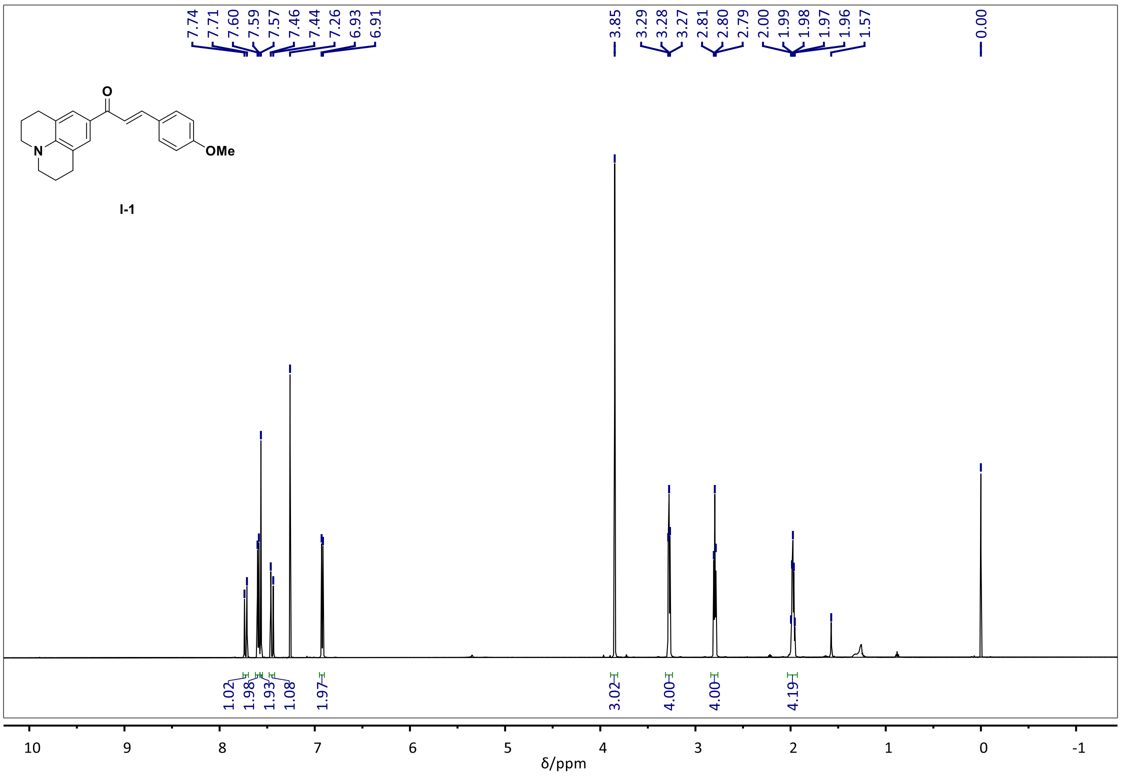

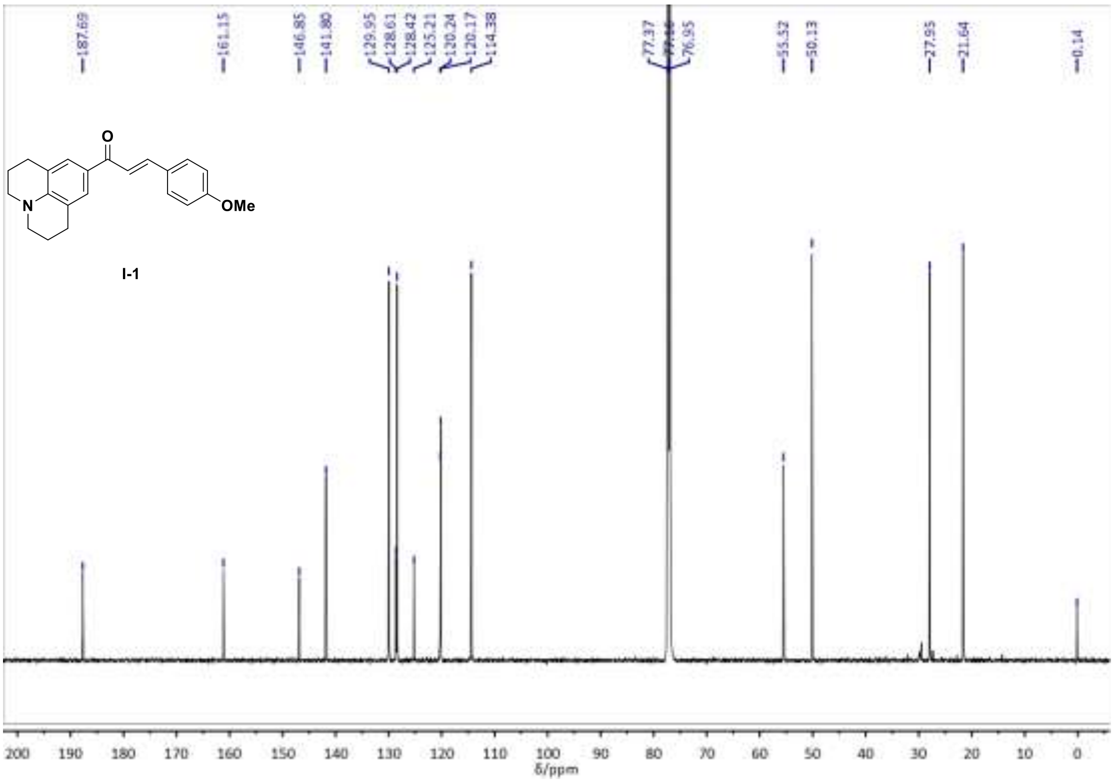

Figure S20 ^{1}H NMR (600 MHz, $CDCl_3$) and ^{13}C NMR (151 MHz, $CDCl_3$) spectra of I-1.

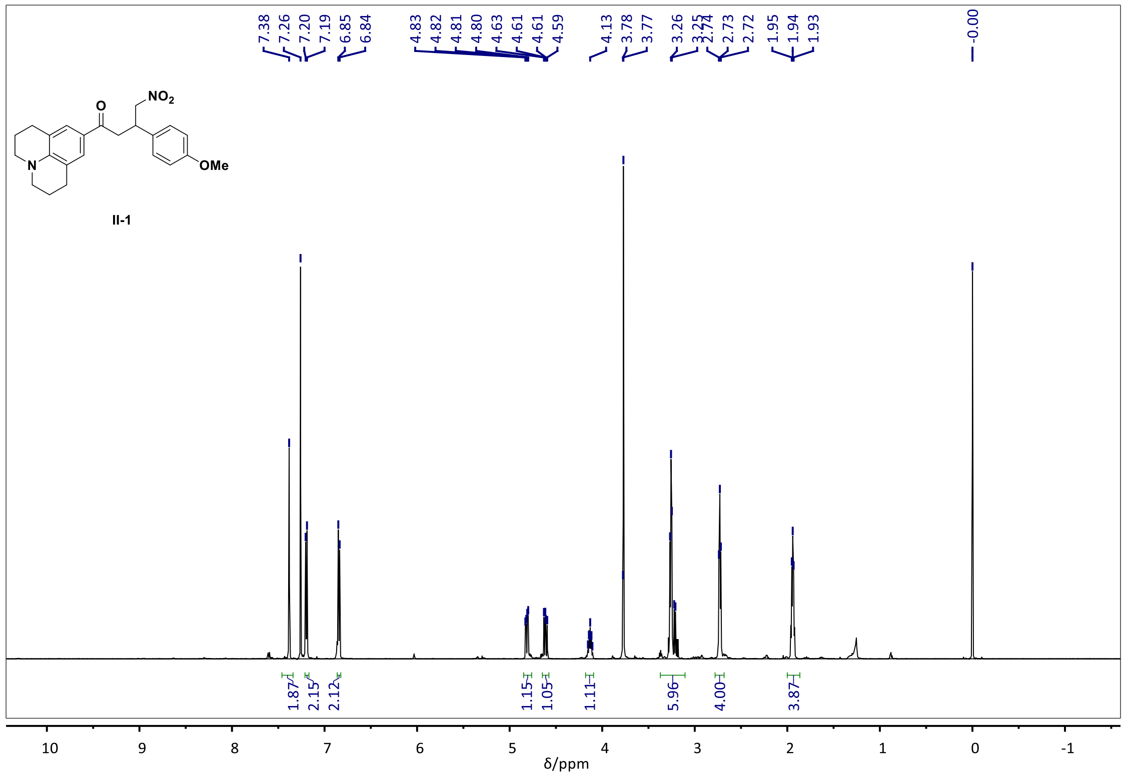

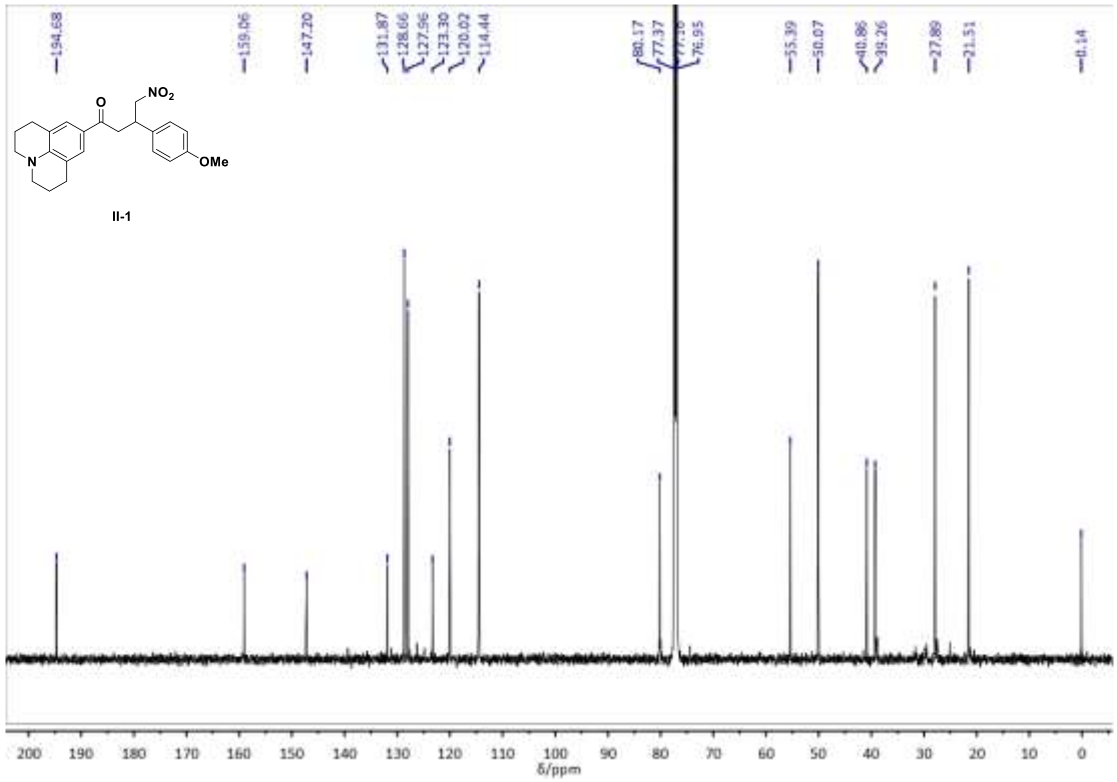

Figure S21 ^{1}H NMR (600 MHz, $CDCl_3$) and ^{13}C NMR (151 MHz, $CDCl_3$) spectra of II-1.

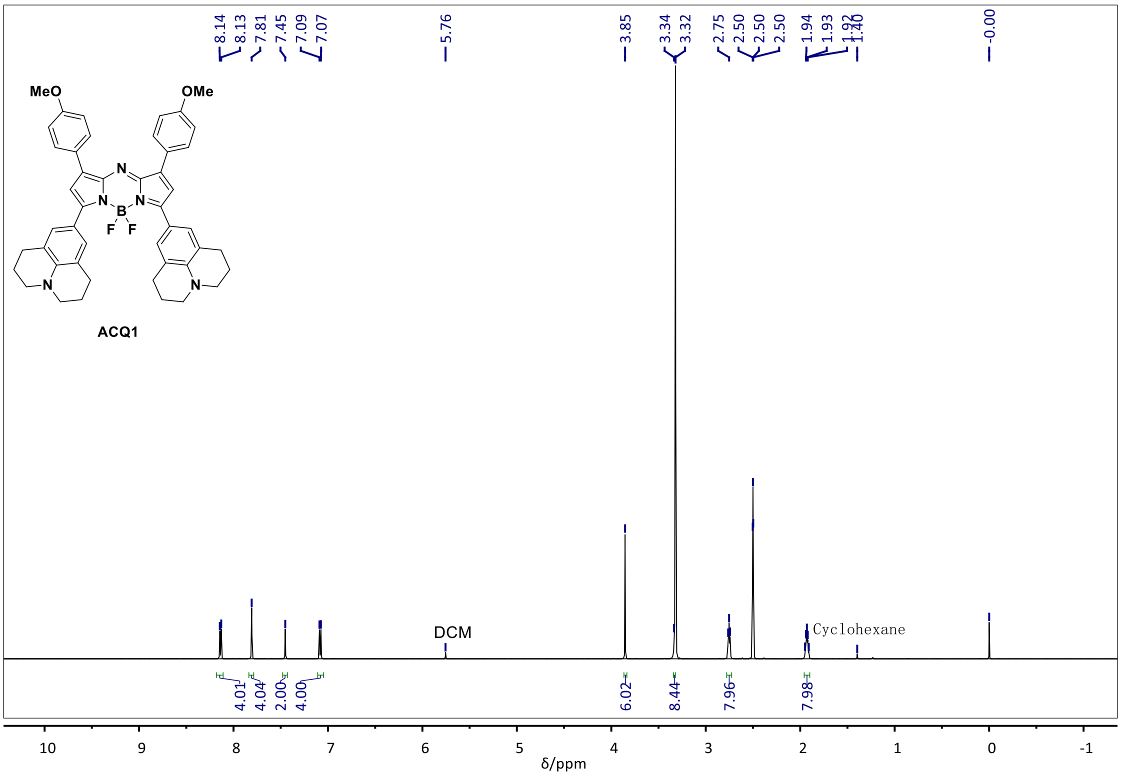

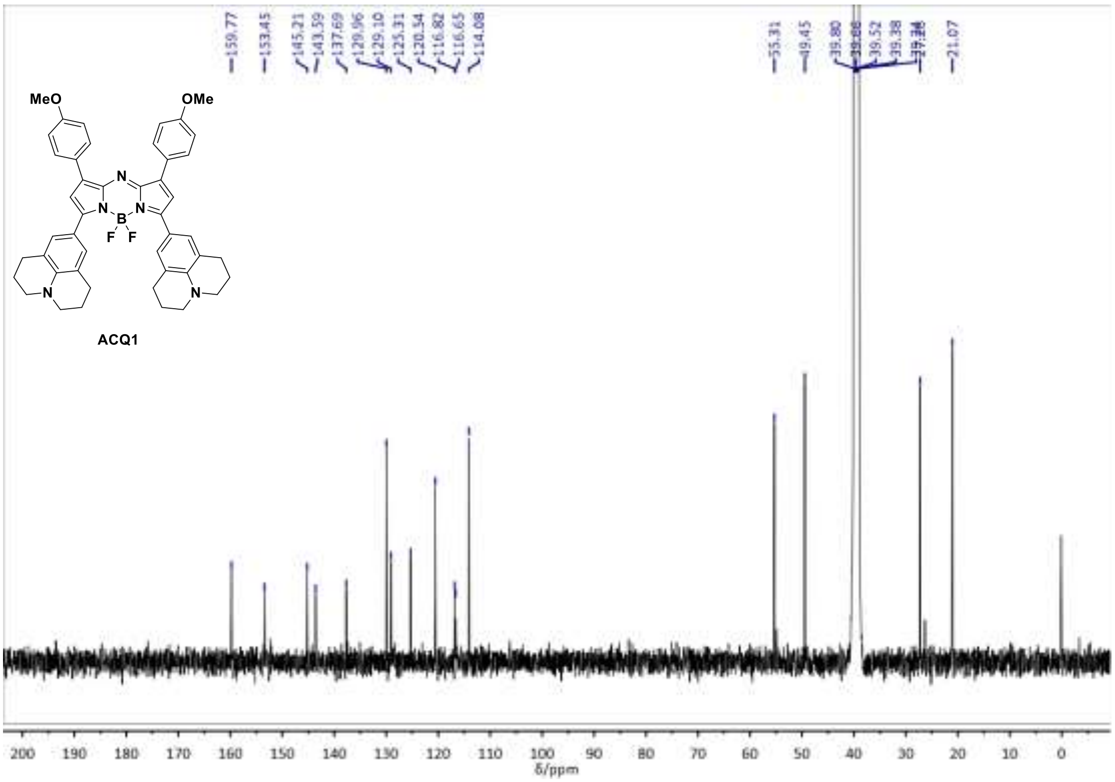

Figure S22 ^{1}H NMR (600 MHz, DMSO-d_6) and ^{13}C NMR (151 MHz, DMSO-d_6) spectra of ACQ1.

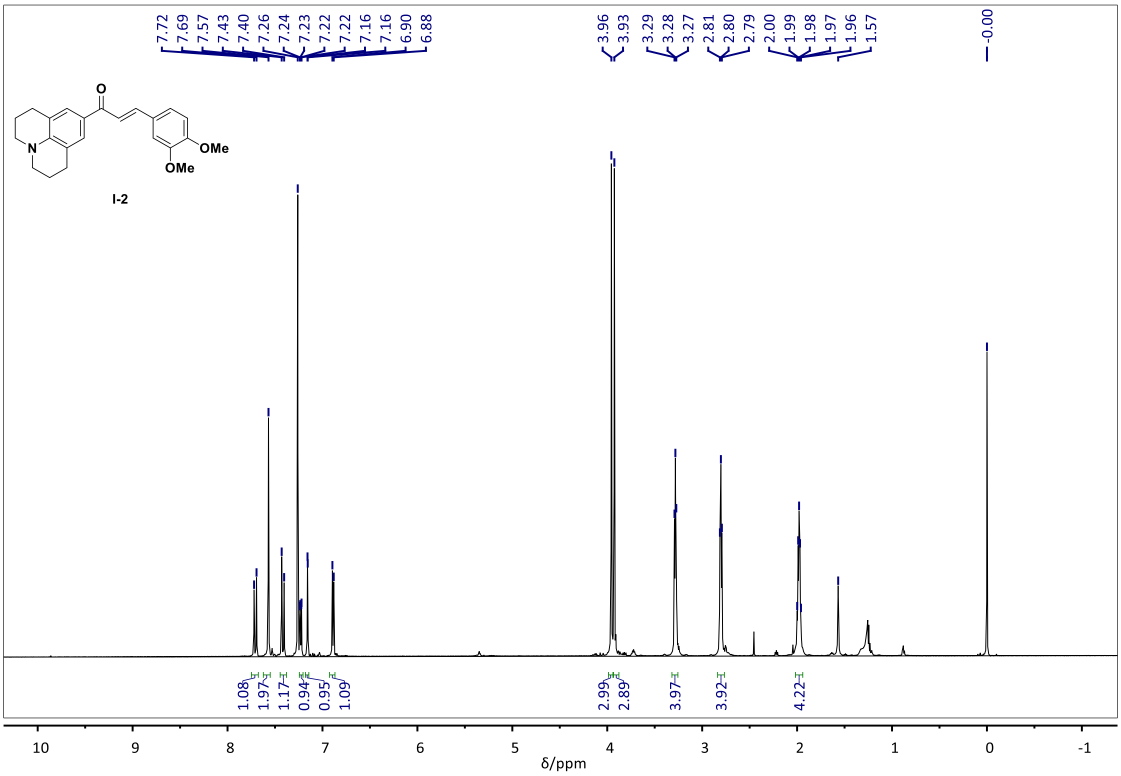

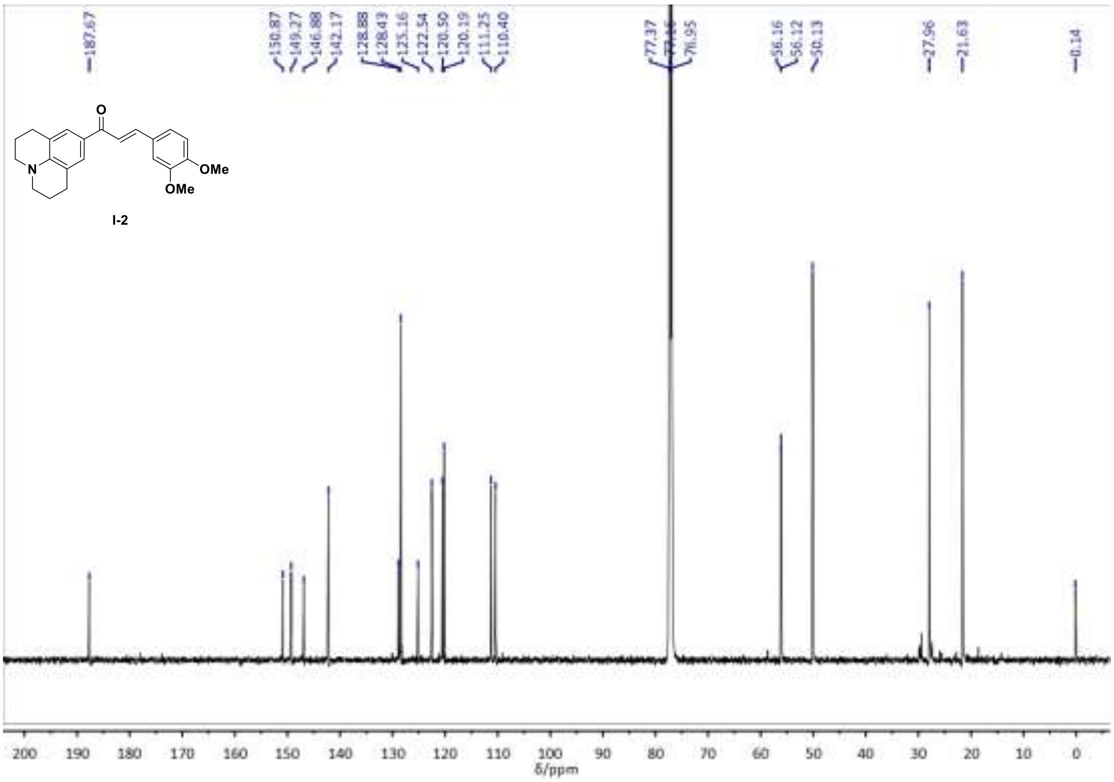

Figure S23 ^{1}H NMR (600 MHz, $CDCl_3$) and ^{13}C NMR (151 MHz, $CDCl_3$) spectra of I-2.

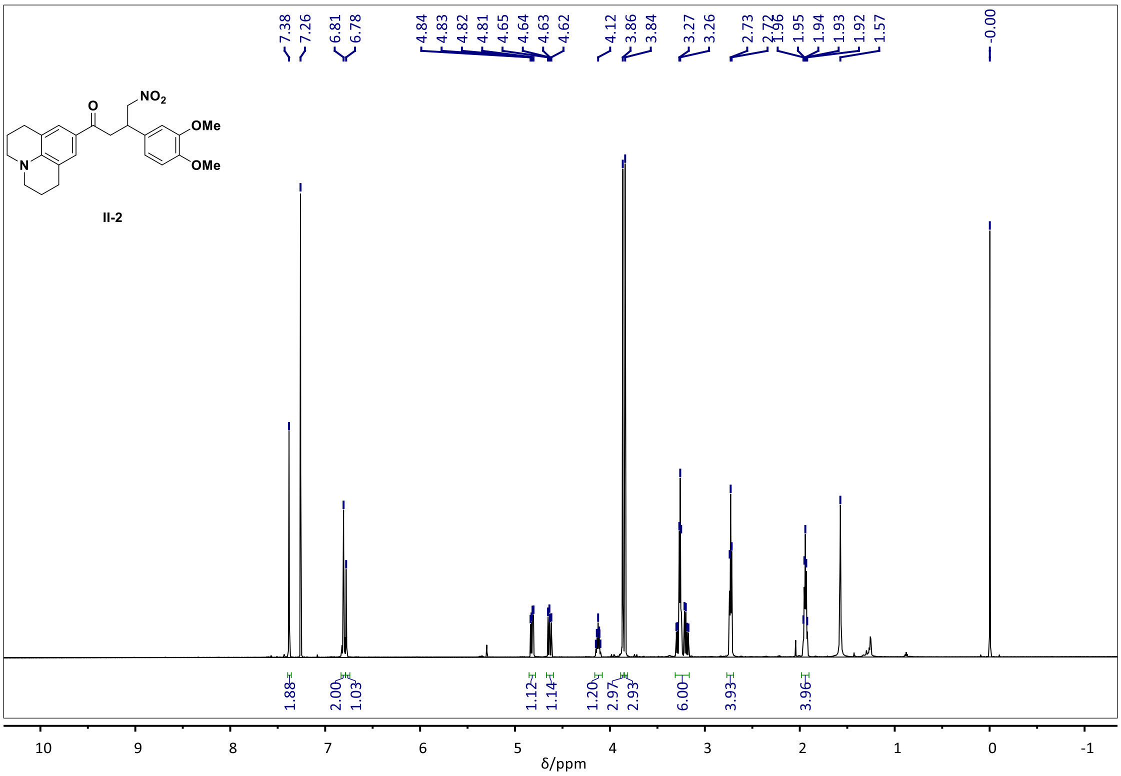

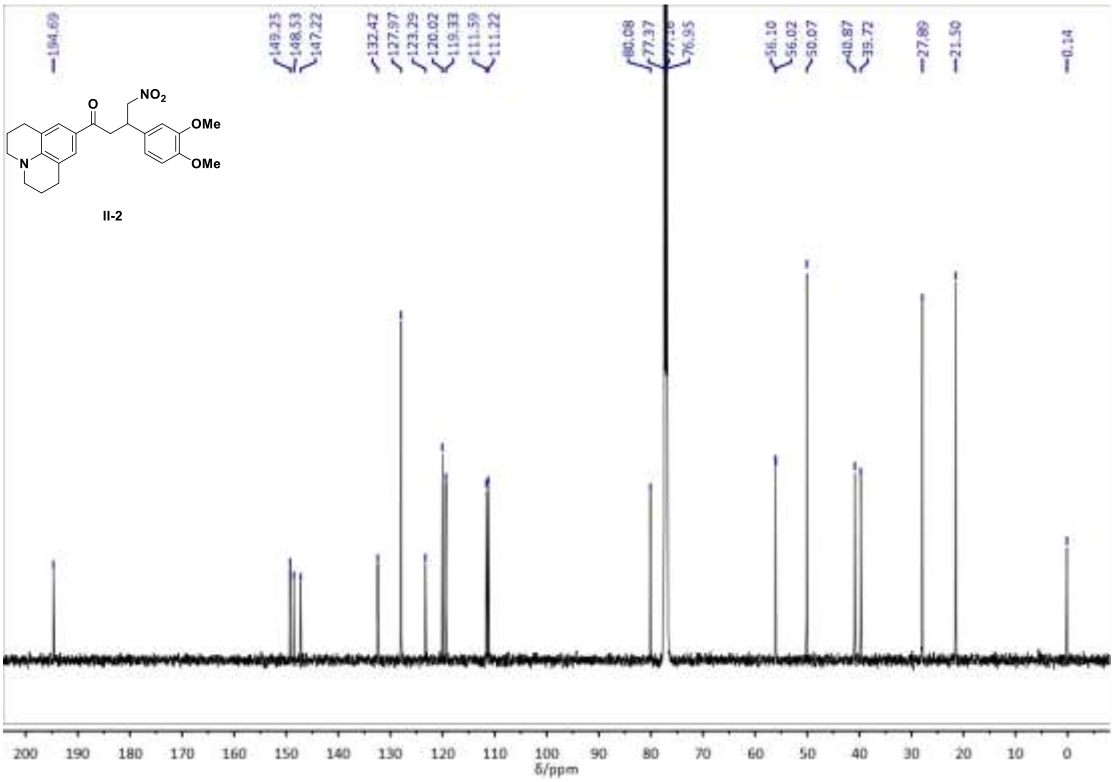

Figure S24 ^{1}H NMR (600 MHz, $CDCl_3$) and ^{13}C NMR (151 MHz, $CDCl_3$) spectra of II-2.

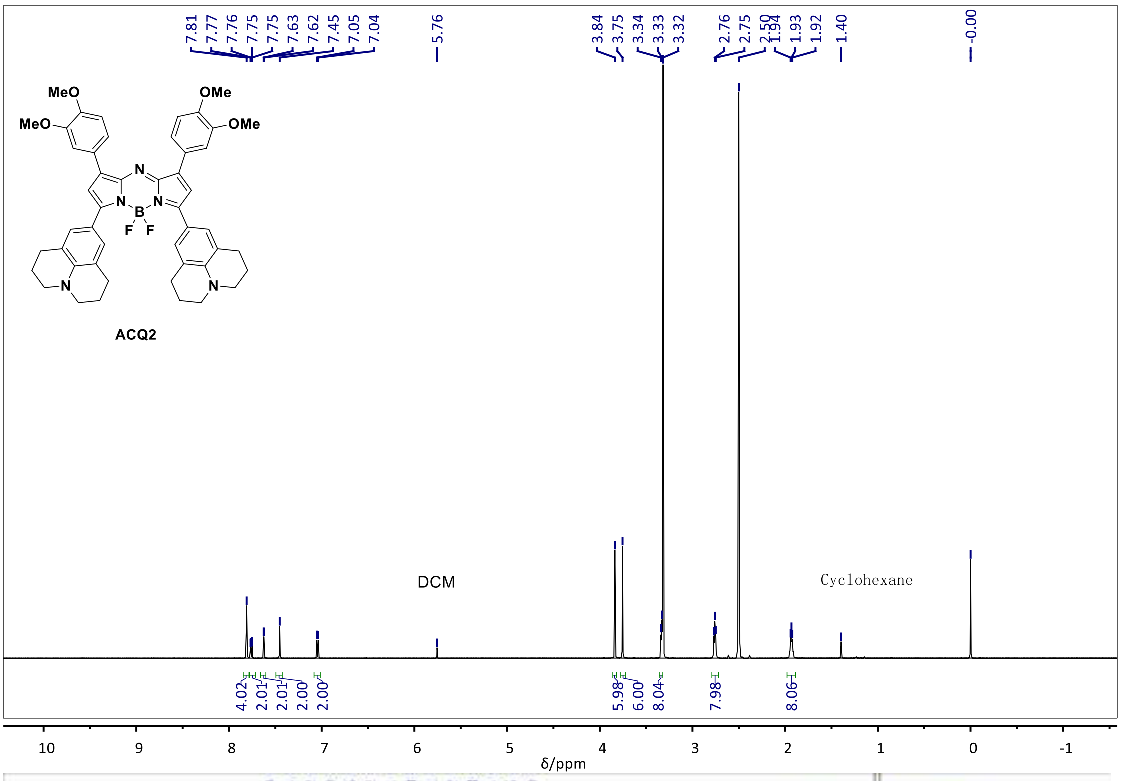

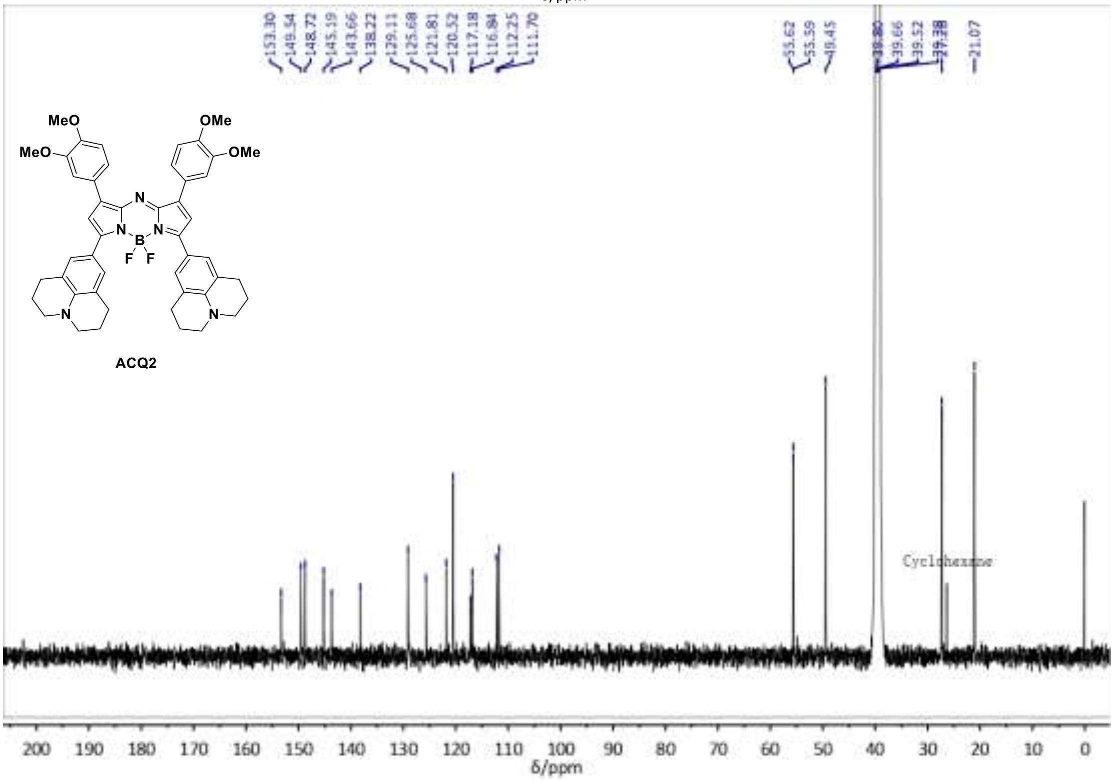

Figure S25 ^{1}H NMR (600 MHz, DMSO-d_6) and ^{13}C NMR (151 MHz, DMSO-d_6) spectra of ACQ2.

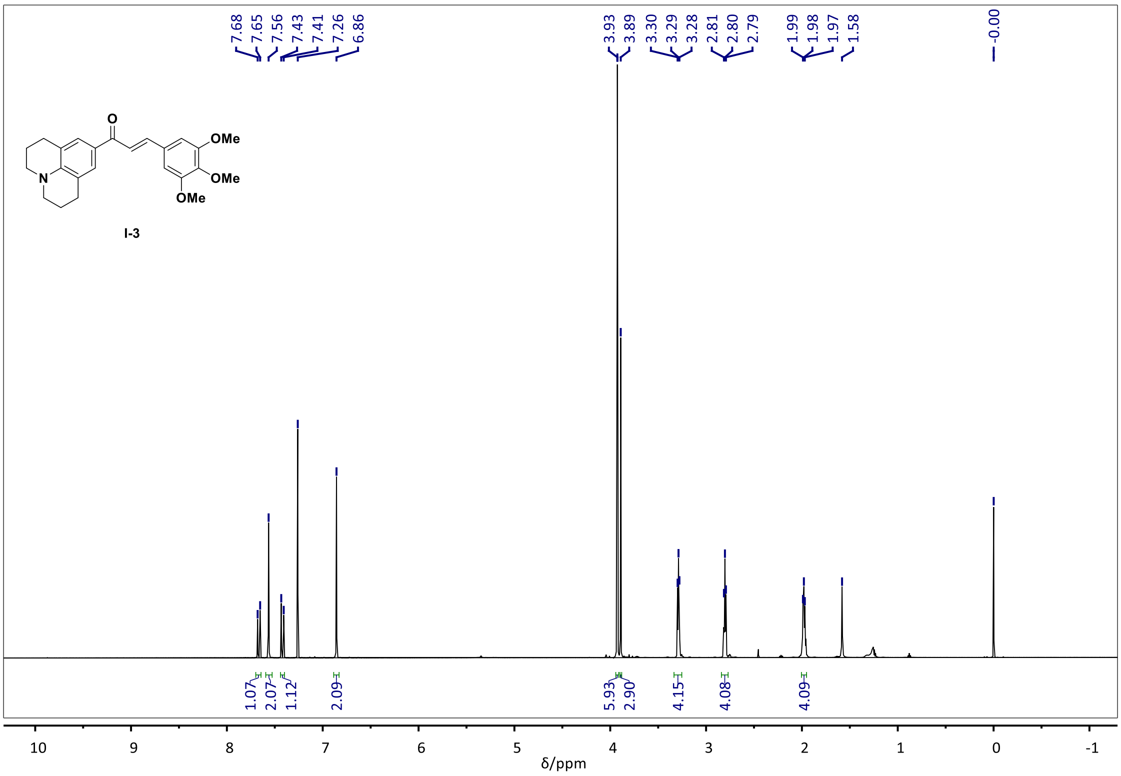

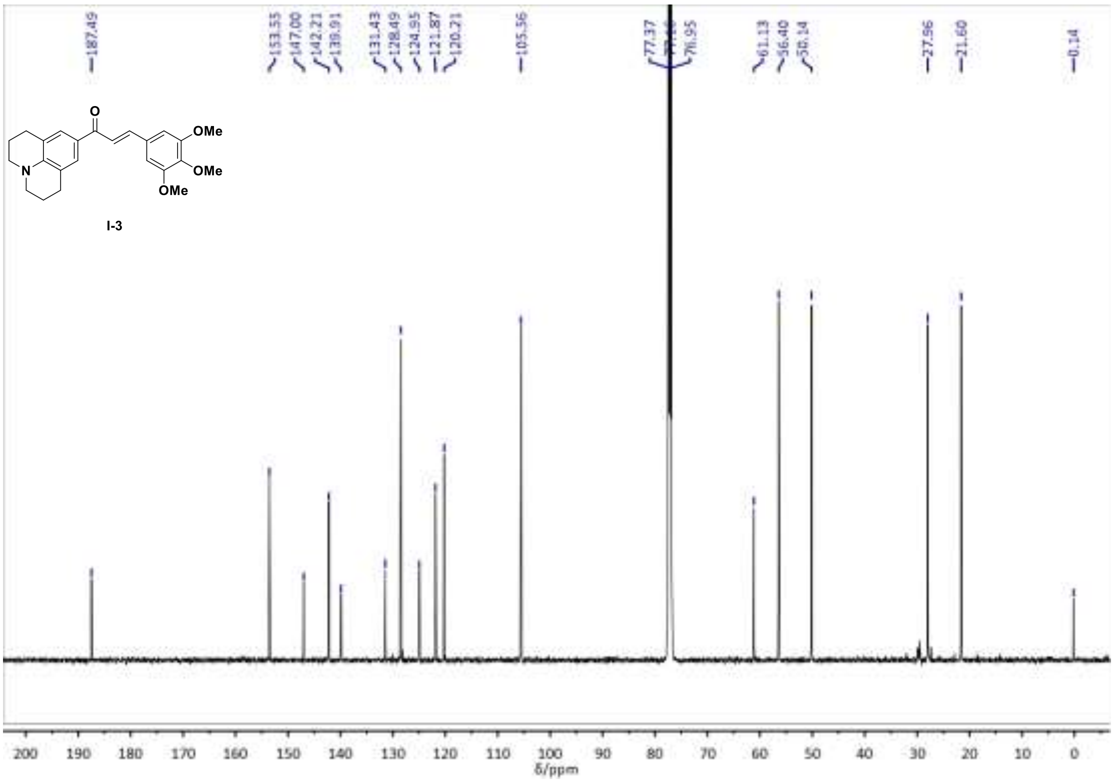

Figure S26 ^{1}H NMR (600 MHz, $CDCl_3$) and ^{13}C NMR (151 MHz, $CDCl_3$) spectra of I-3.

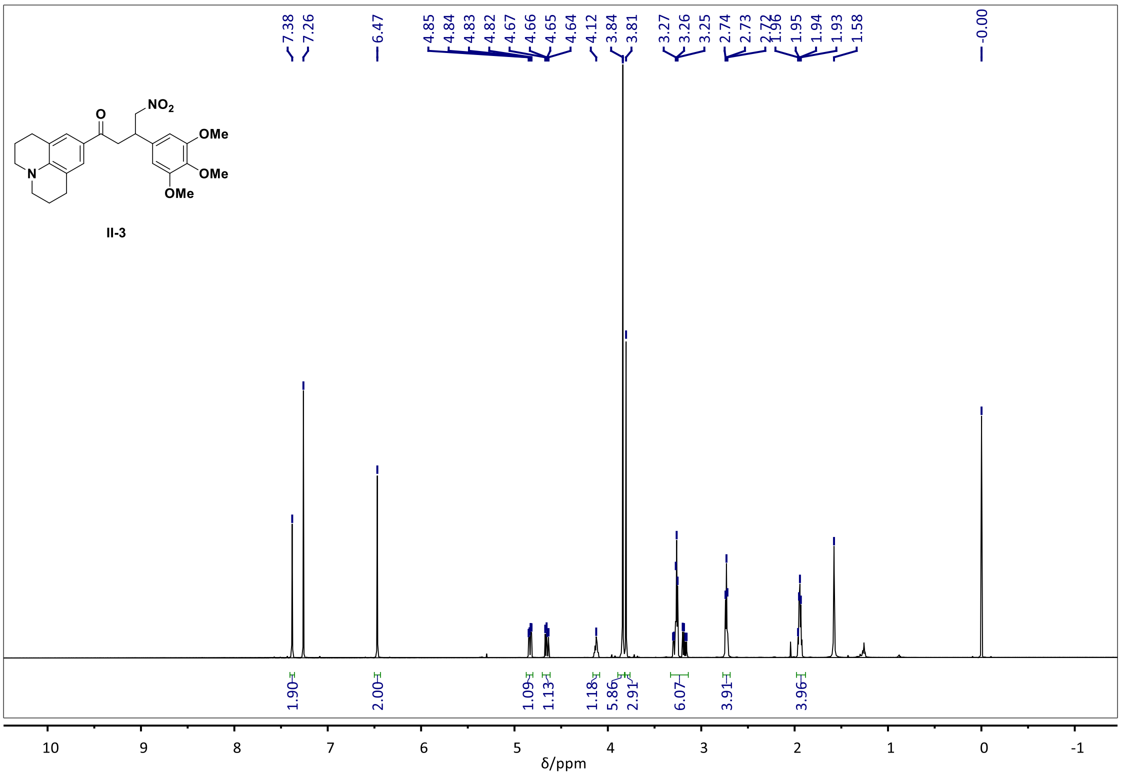

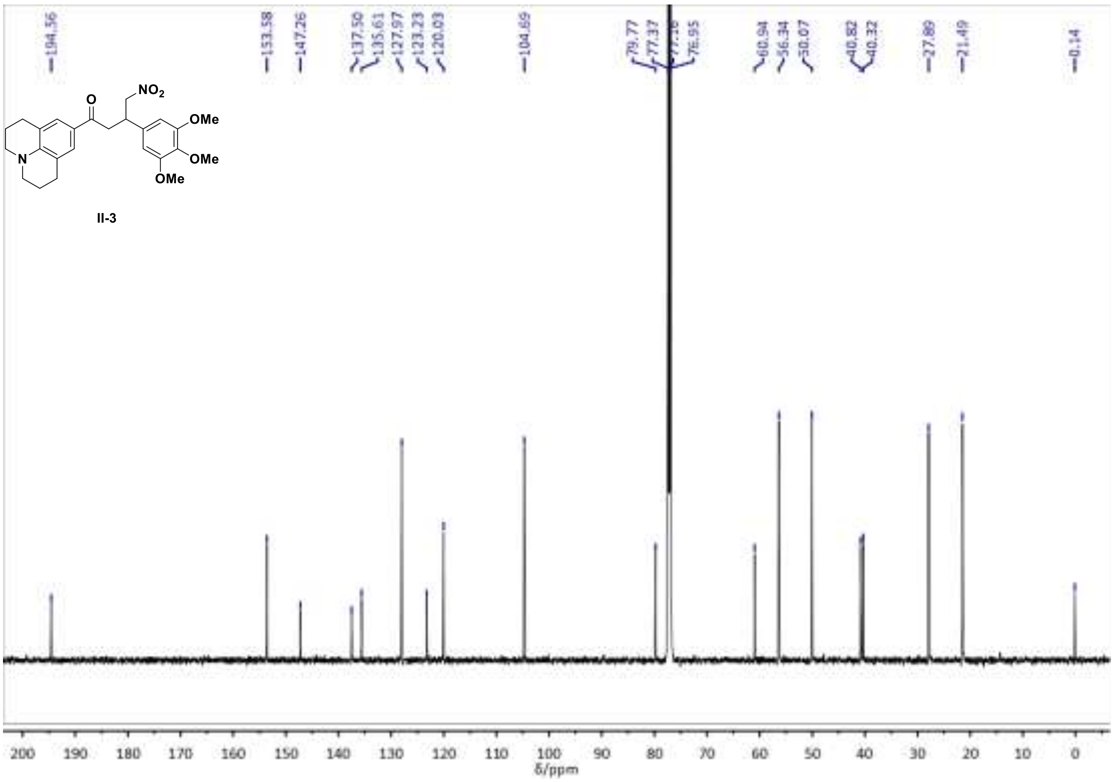

Figure S27 ^{1}H NMR (600 MHz, $CDCl_3$) and ^{13}C NMR (151 MHz, $CDCl_3$) spectra of II-3.

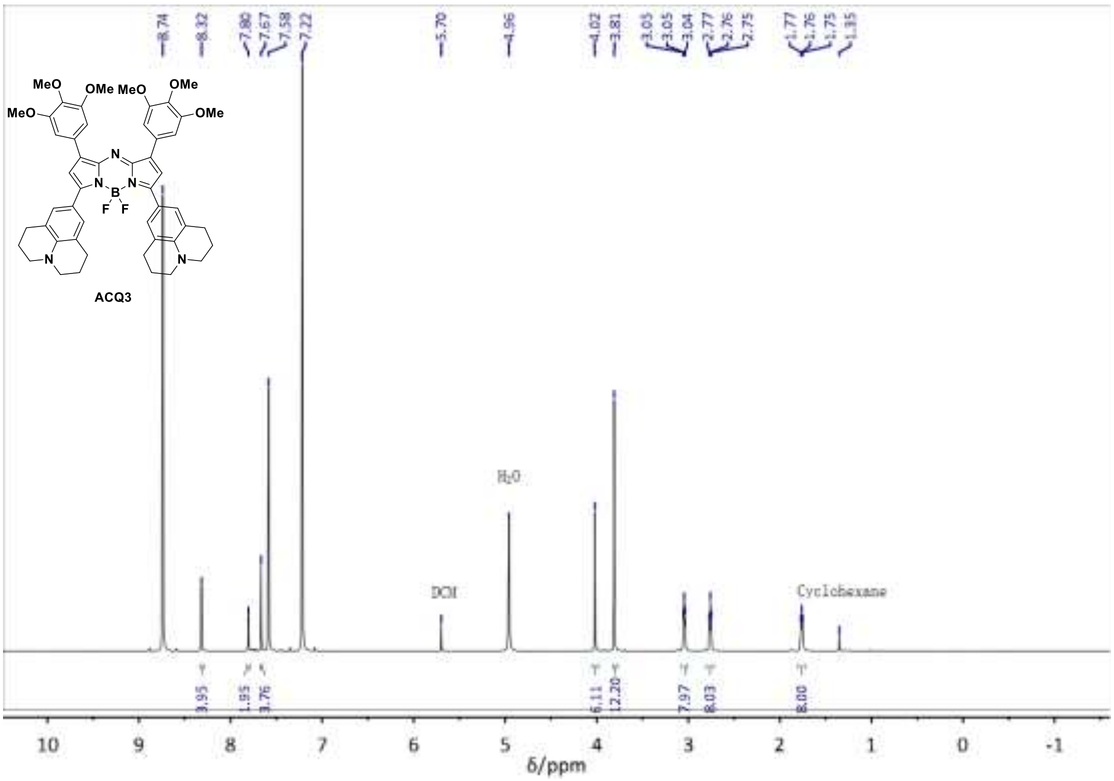

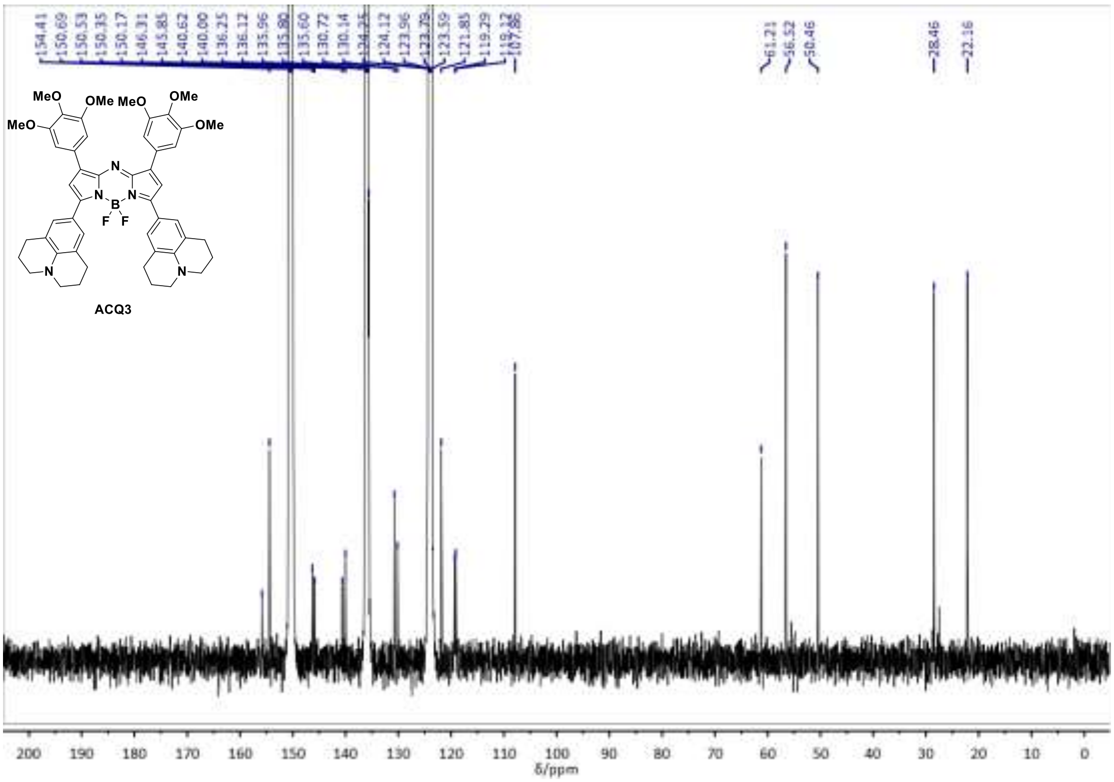

Figure S28 ^{1}H NMR (600 MHz, pyridine-d_5) and ^{13}C NMR (151 MHz, pyridine-d_5) spectra of ACQ3.

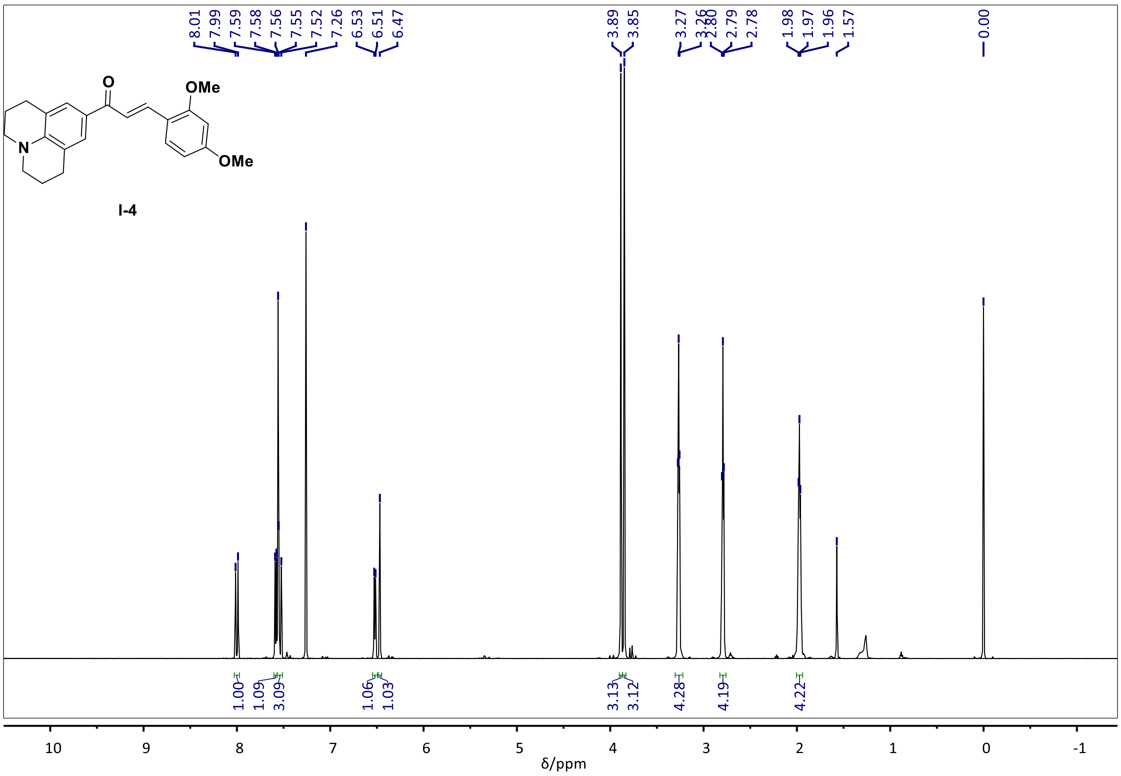

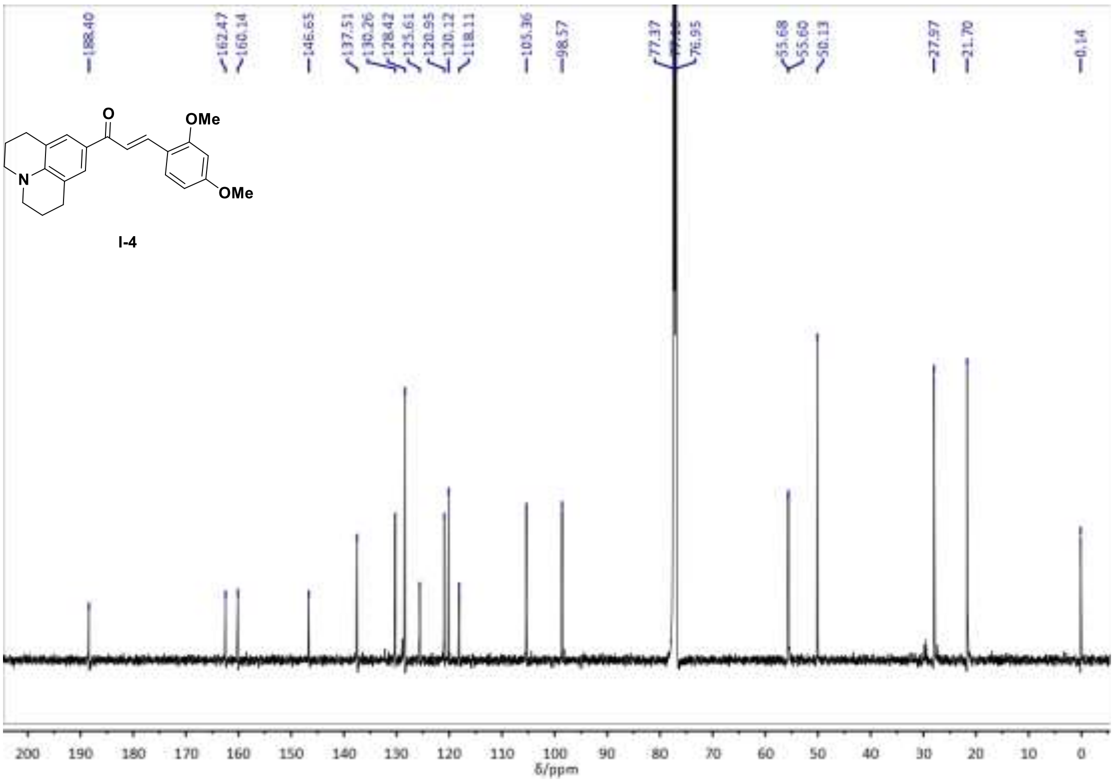

Figure S29 ^{1}H NMR (600 MHz, $CDCl_3$) and ^{13}C NMR (151 MHz, $CDCl_3$) spectra of I-4.

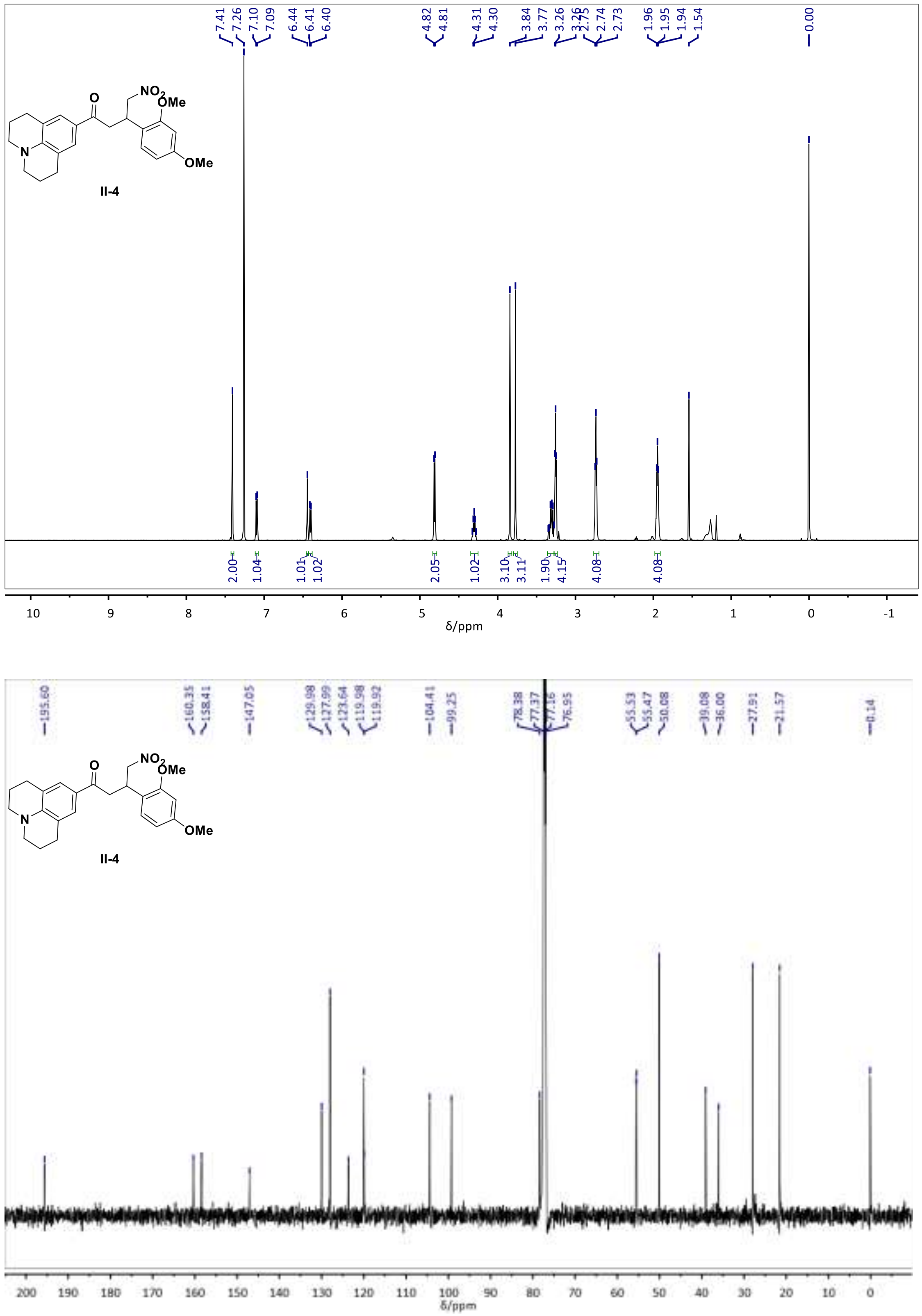

Figure S30 ^{1}H NMR (600 MHz, $CDCl_3$) and ^{13}C NMR (151 MHz, $CDCl_3$) spectra of II-4.

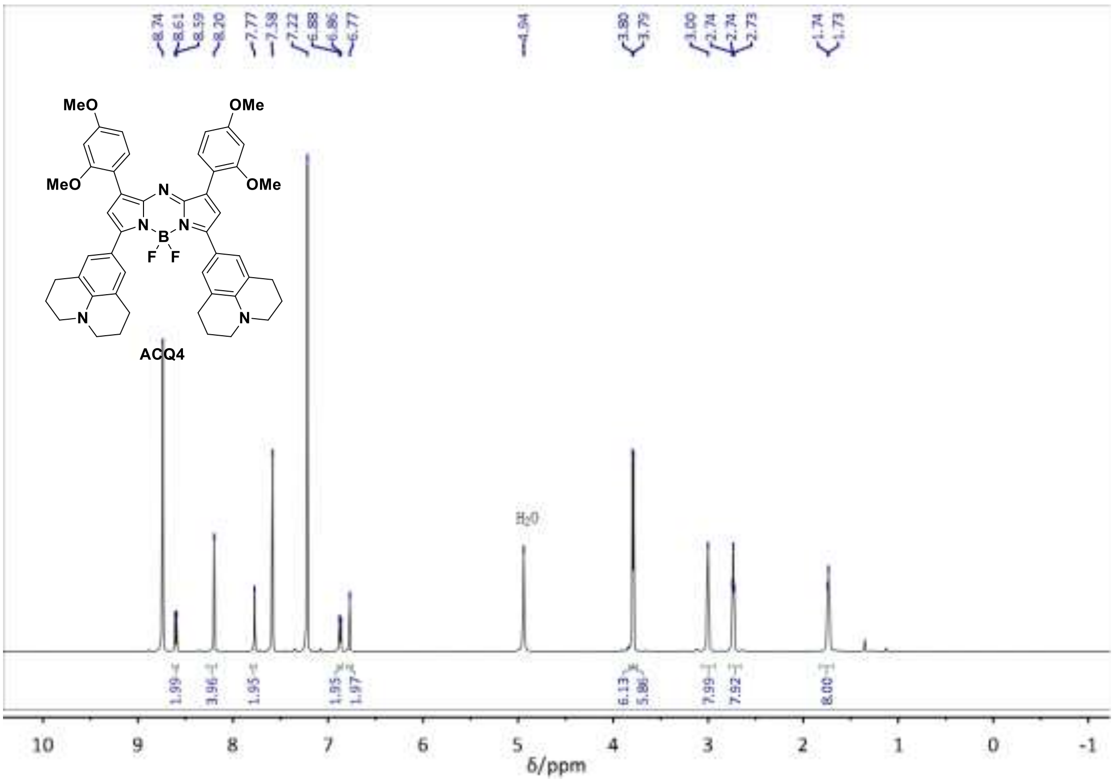

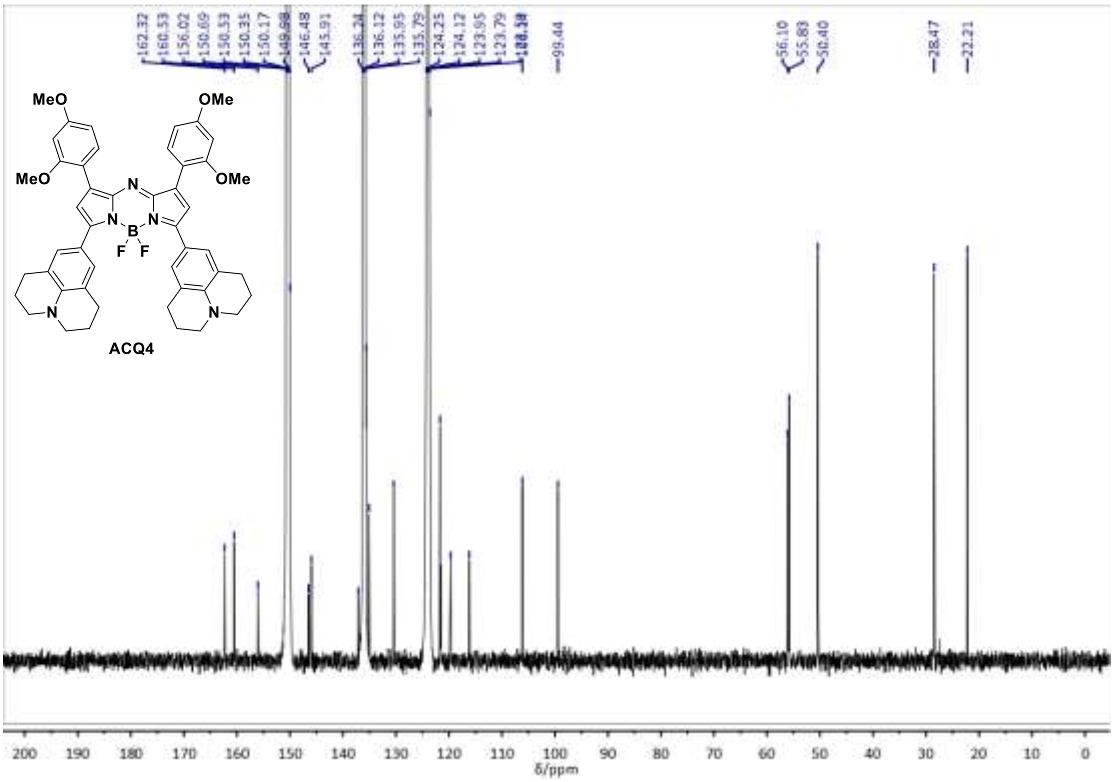

Figure S31 ^{1}H NMR (600 MHz, pyridine-d_5) and ^{13}C NMR (151 MHz, pyridine-d_5) spectra of ACQ4.

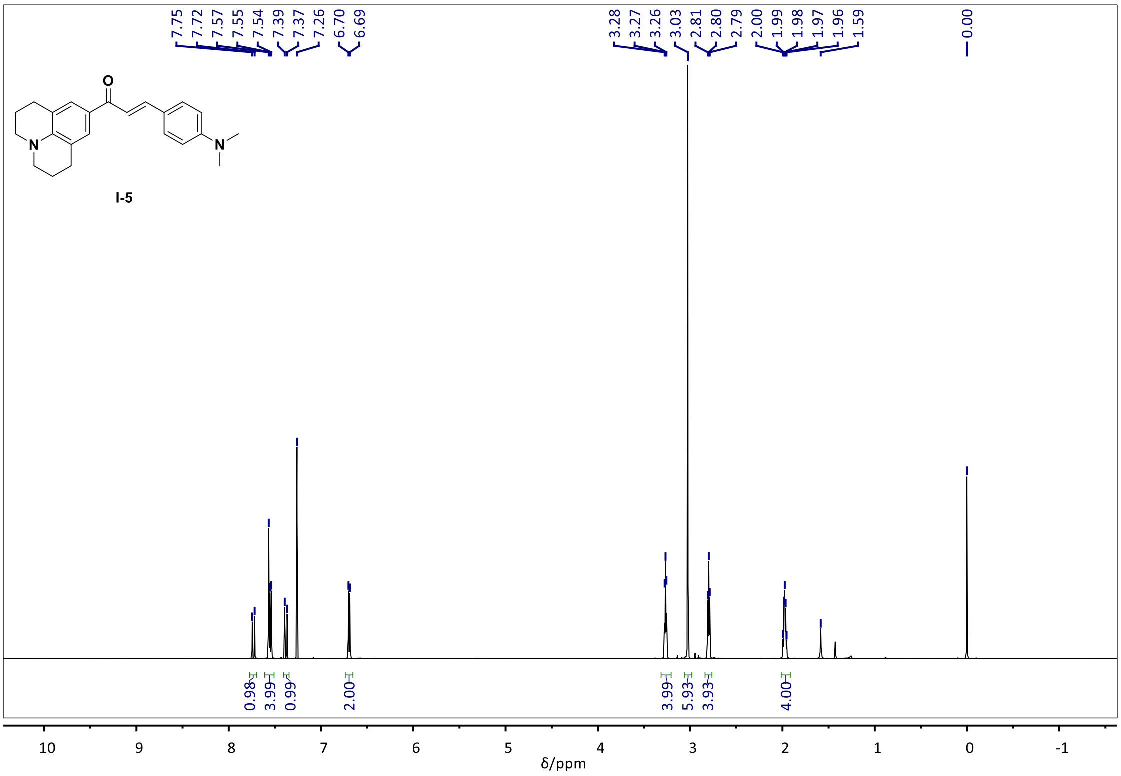

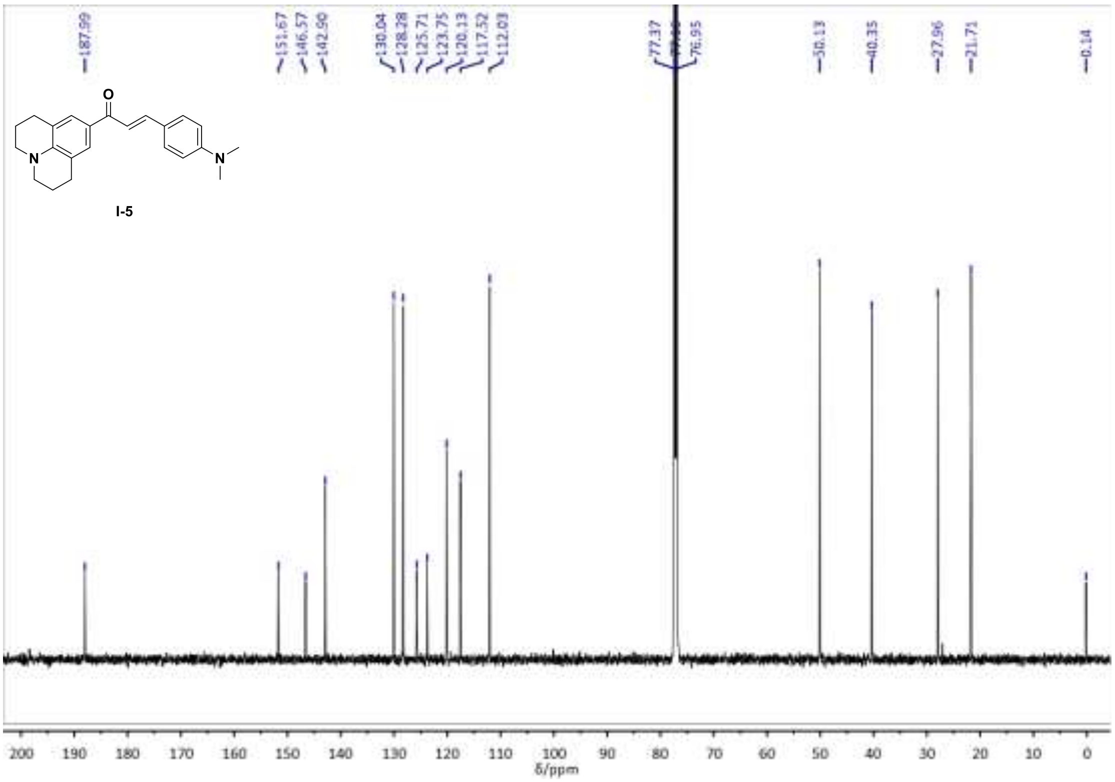

Figure S32 ^{1}H NMR (600 MHz, $CDCl_3$) and ^{13}C NMR (151 MHz, $CDCl_3$) spectra of I-5.

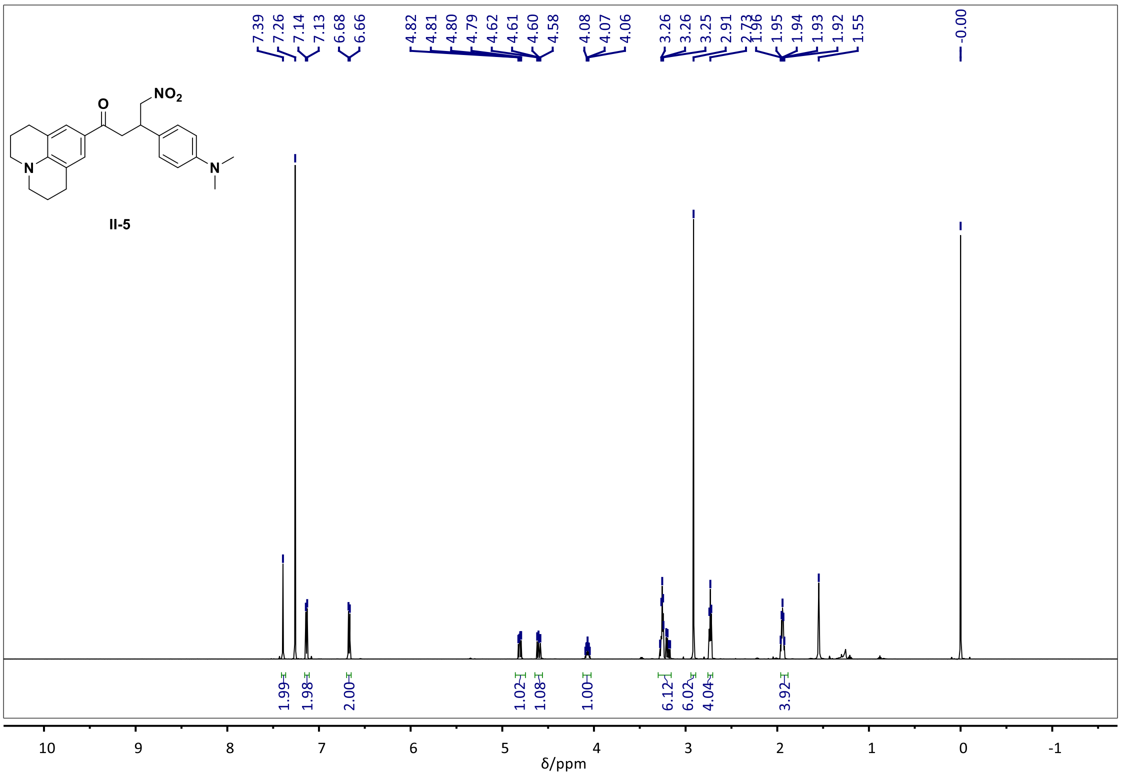

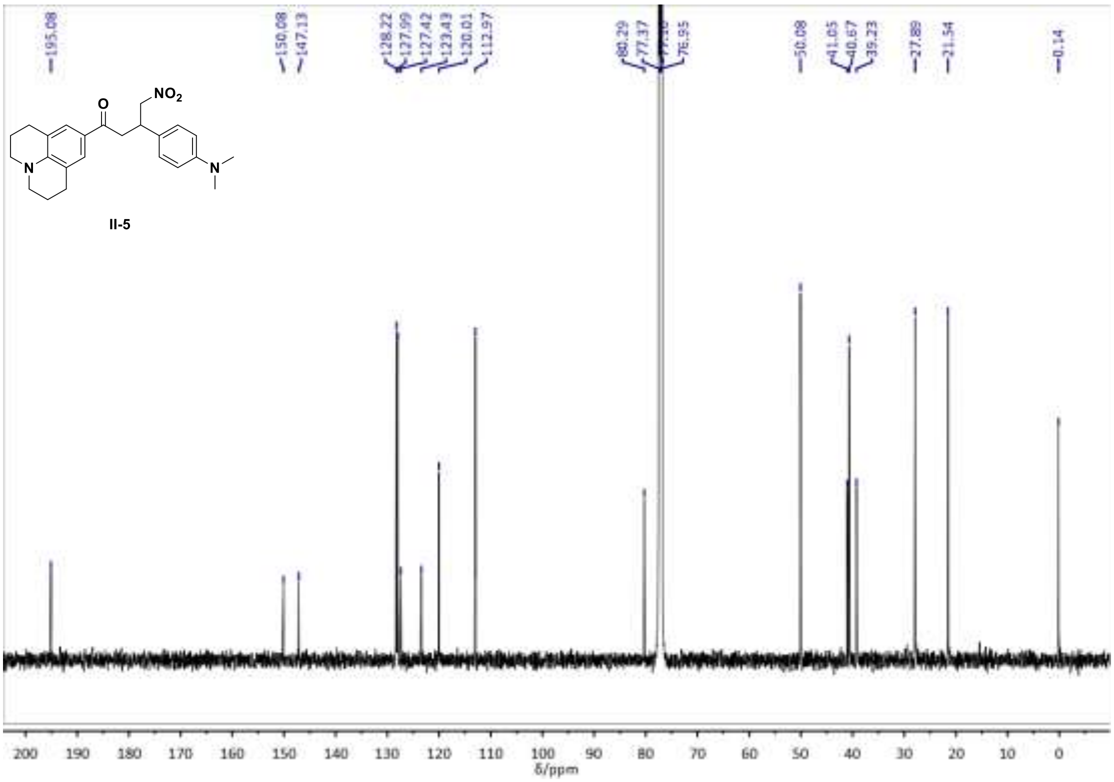

Figure S33 ^{1}H NMR (600 MHz, $CDCl_3$) and ^{13}C NMR (151 MHz, $CDCl_3$) spectra of II-5.

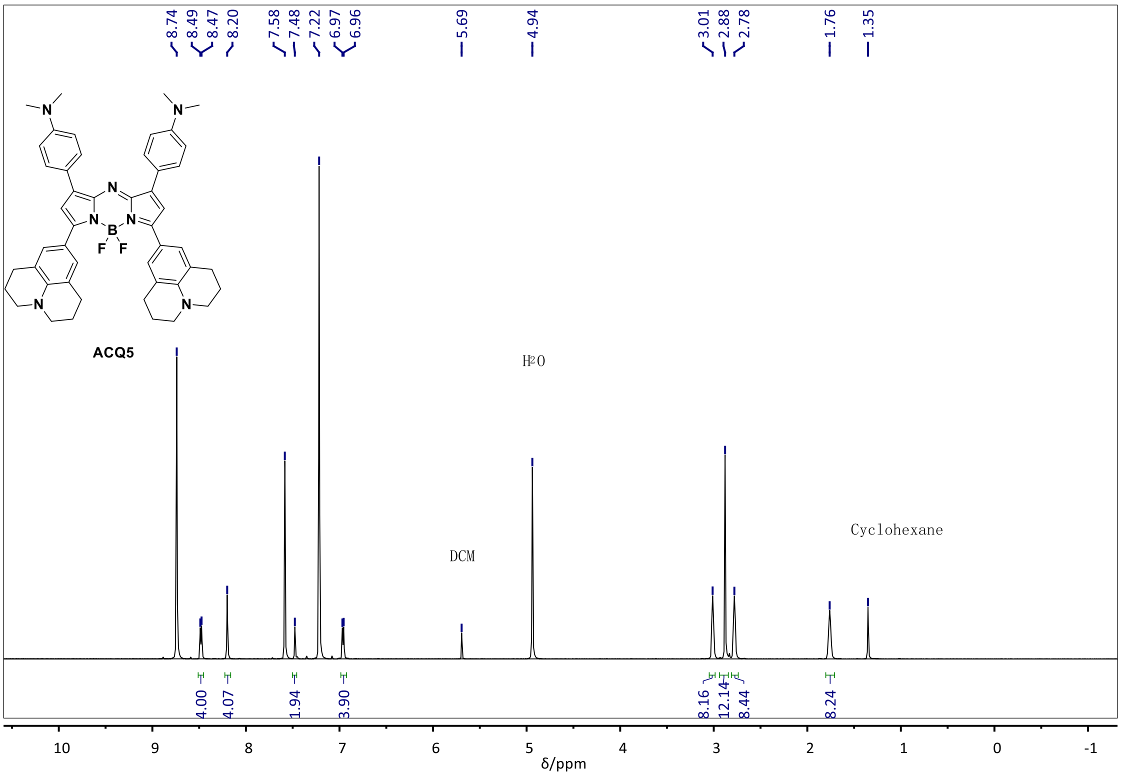

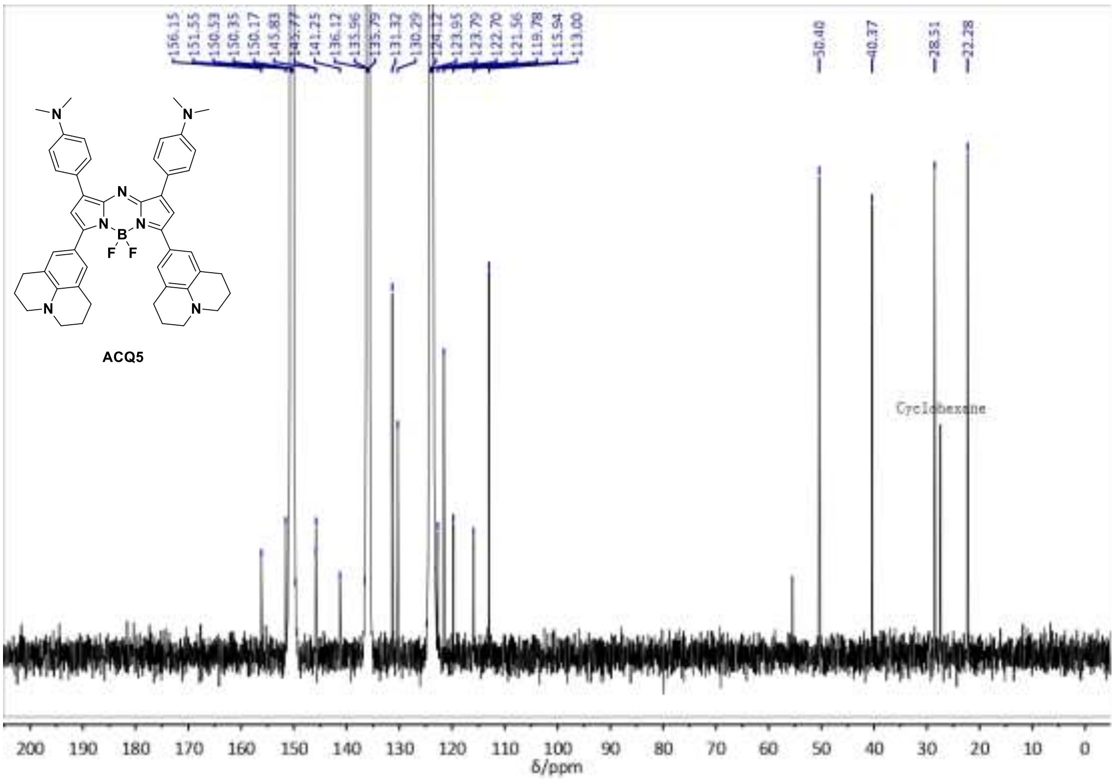

Figure S34 ^{1}H NMR (600 MHz, pyridine-d_5) and ^{13}C NMR (151 MHz, pyridine-d_5) spectra of ACQ5.

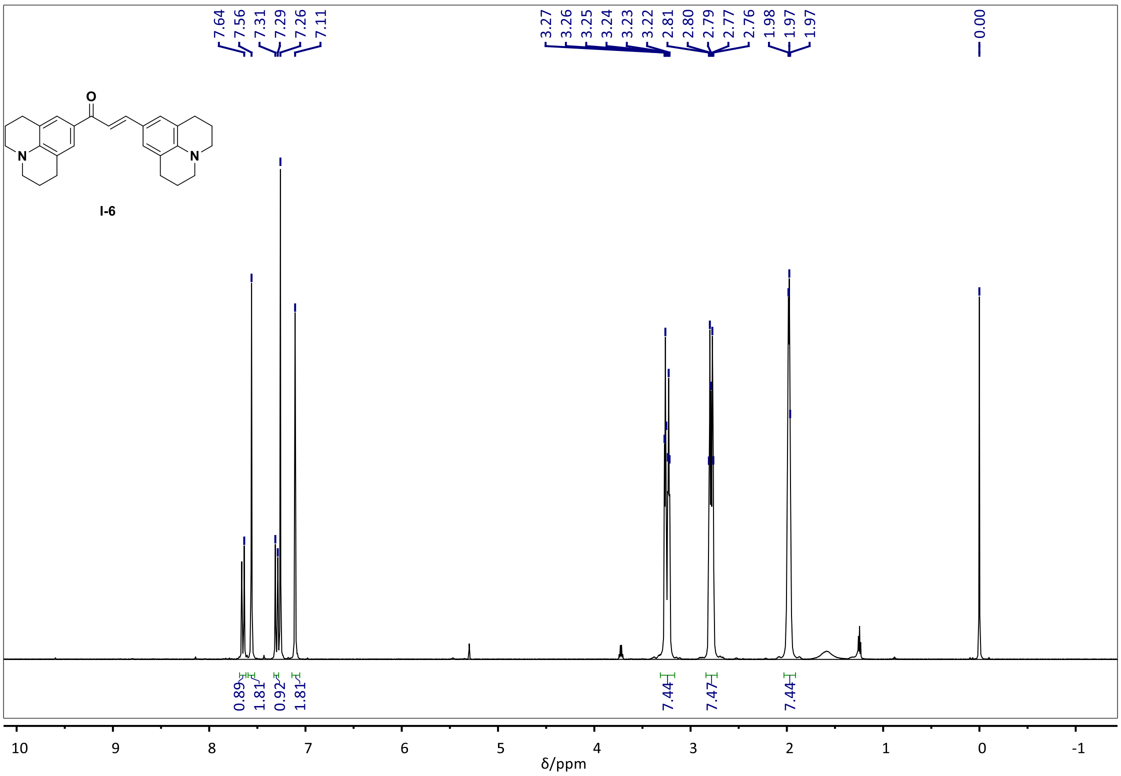

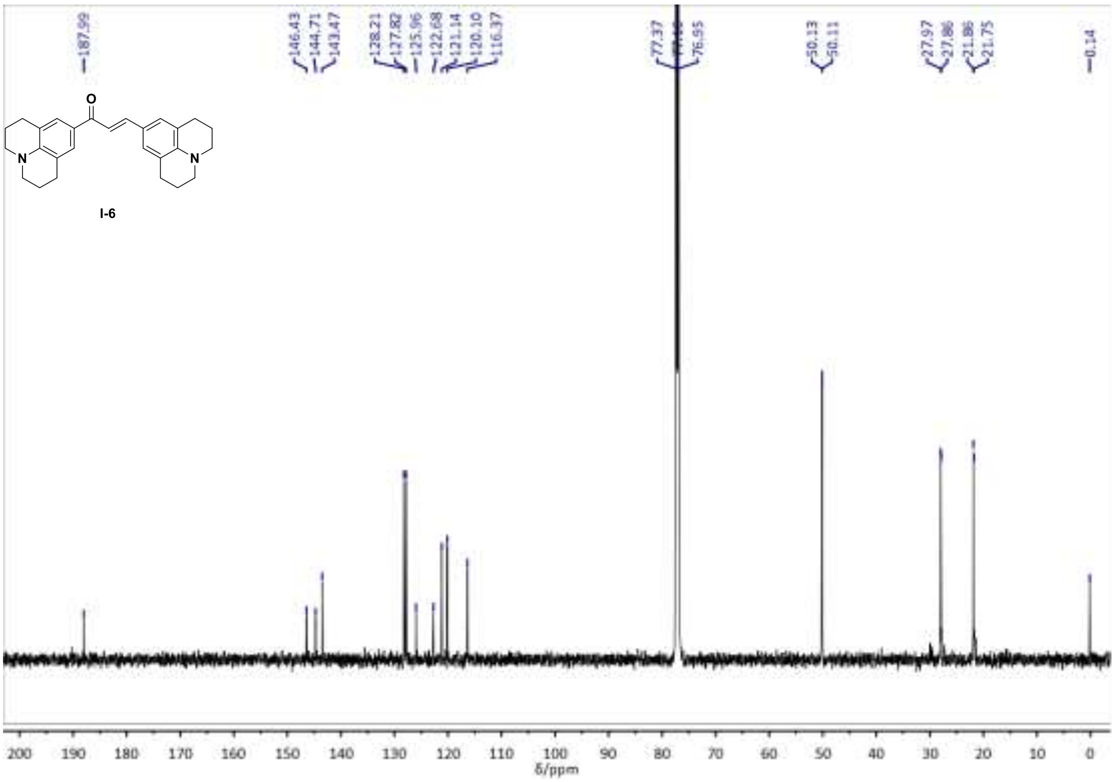

Figure S35 ^{1}H NMR (600 MHz, $CDCl_3$) and ^{13}C NMR (151 MHz, $CDCl_3$) spectra of I-6.

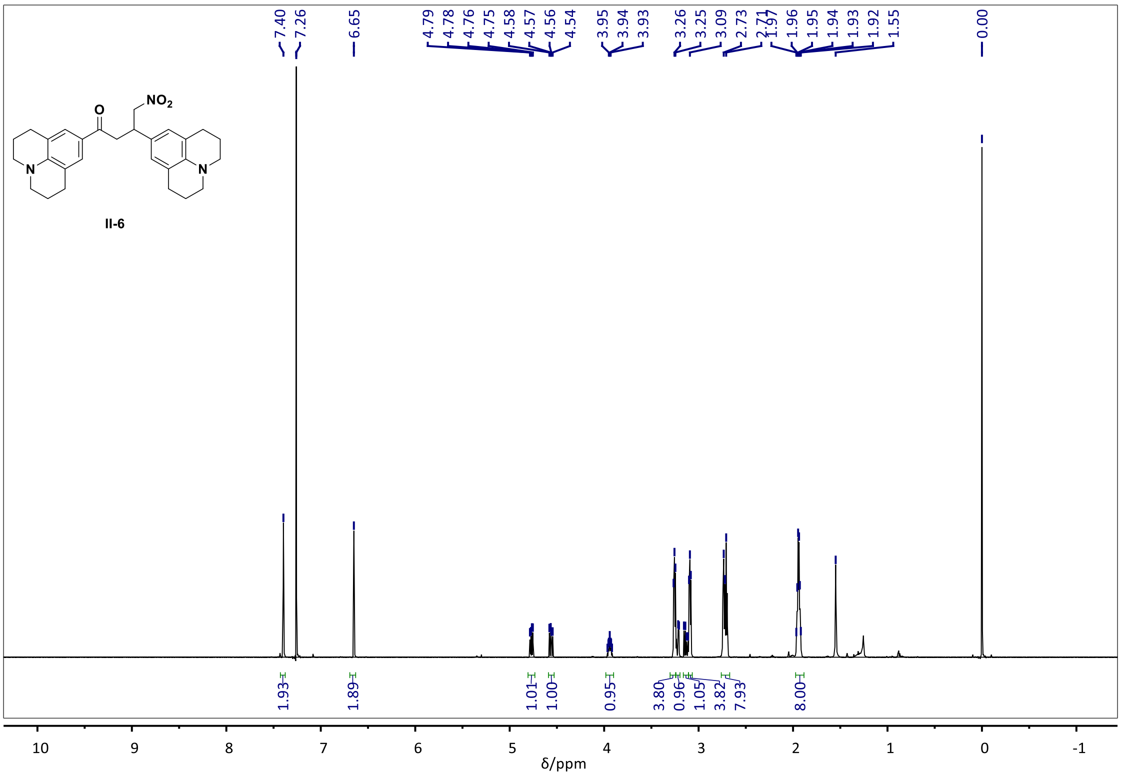

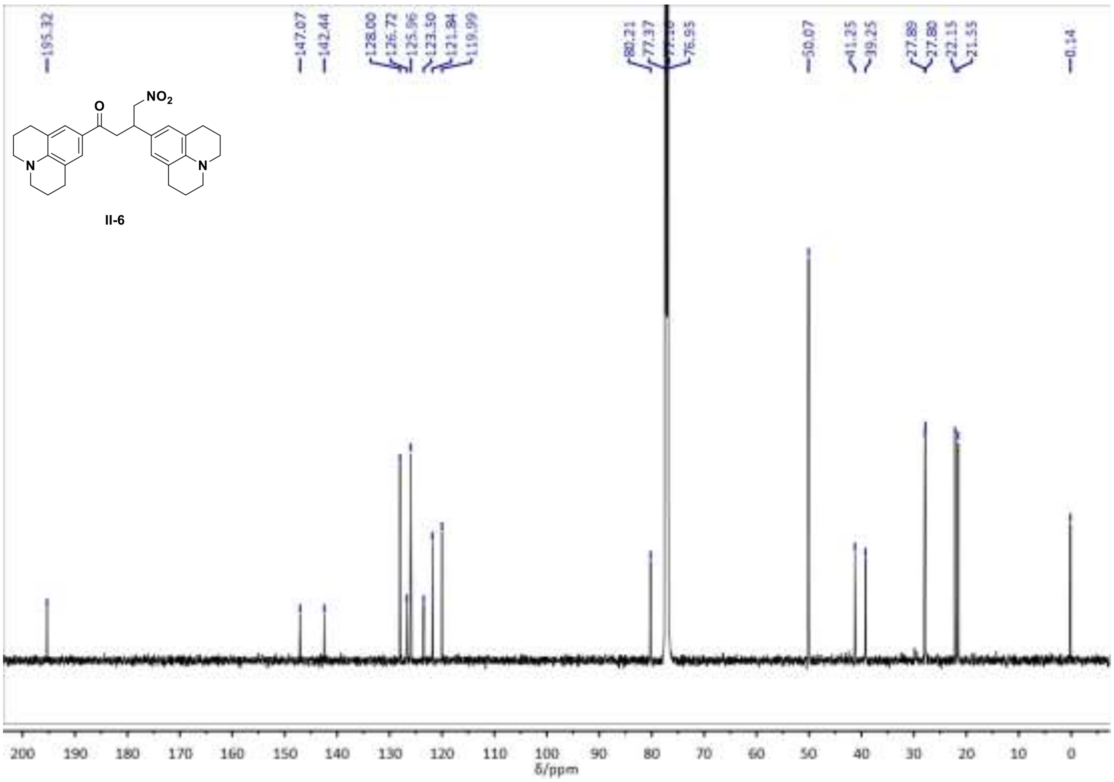

Figure S36 ^{1}H NMR (600 MHz, $CDCl_3$) and ^{13}C NMR (151 MHz, $CDCl_3$) spectra of II-6.

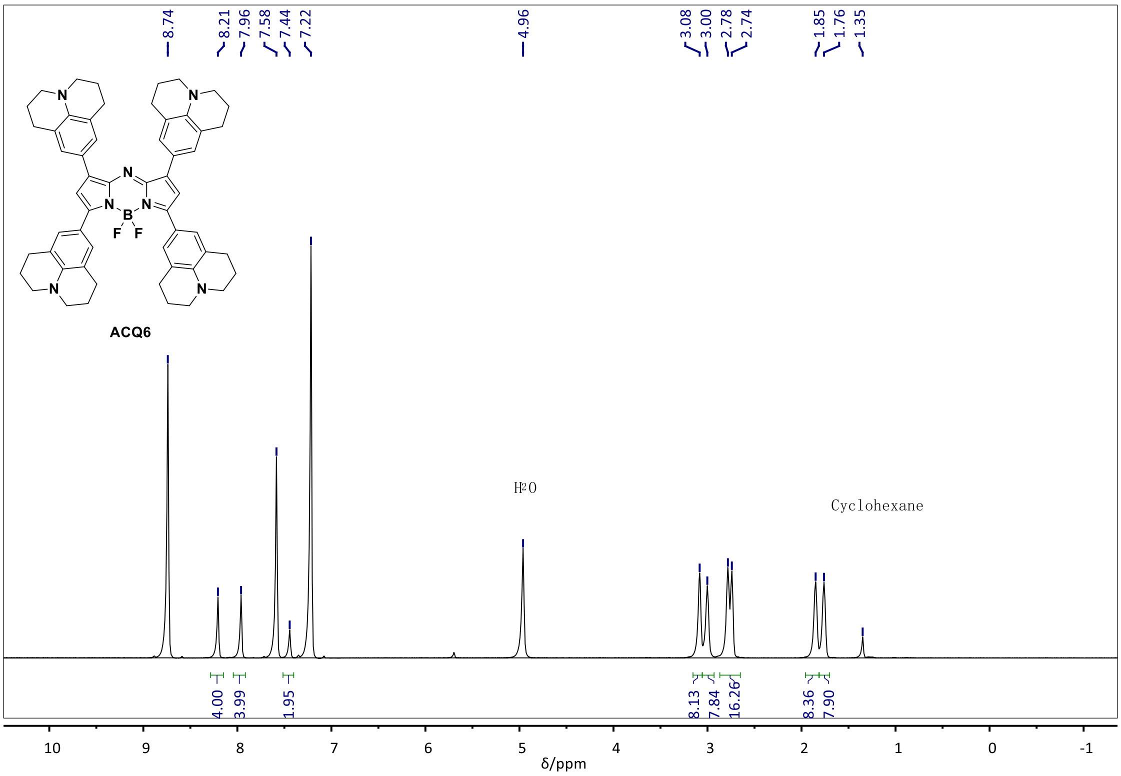

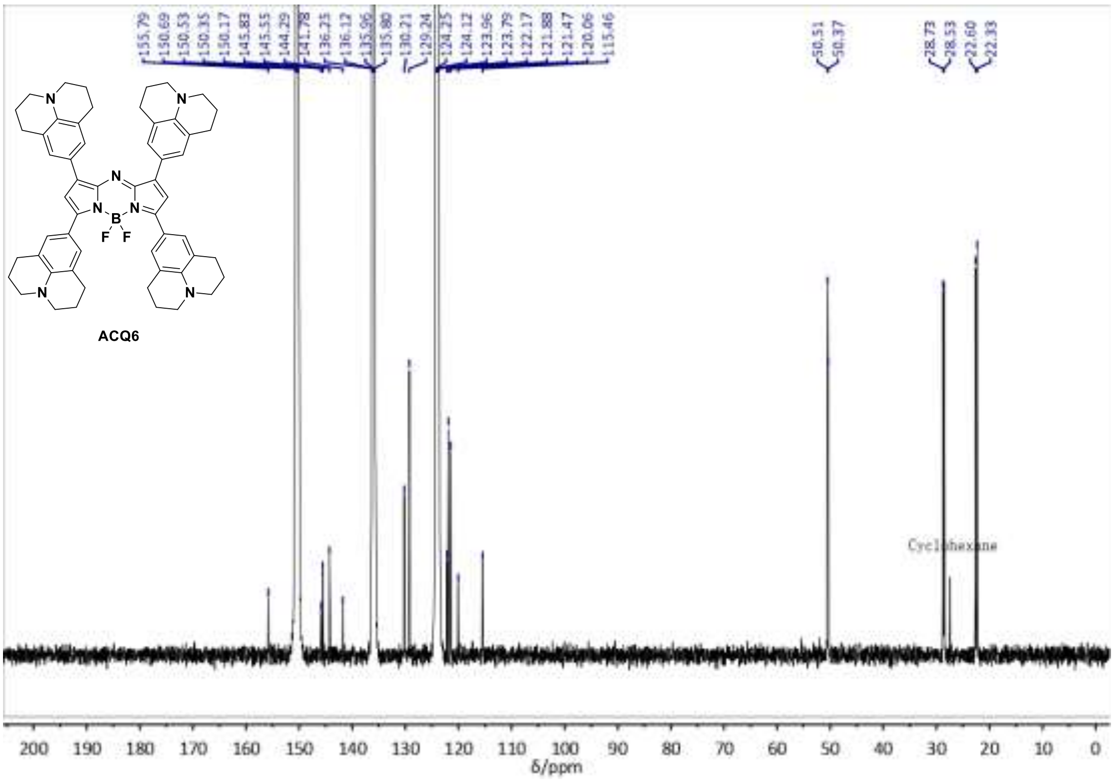

Figure S37 ^{1}H NMR (600 MHz, pyridine-d_5) and ^{13}C NMR (151 MHz, pyridine-d_5) spectra of ACQ6.

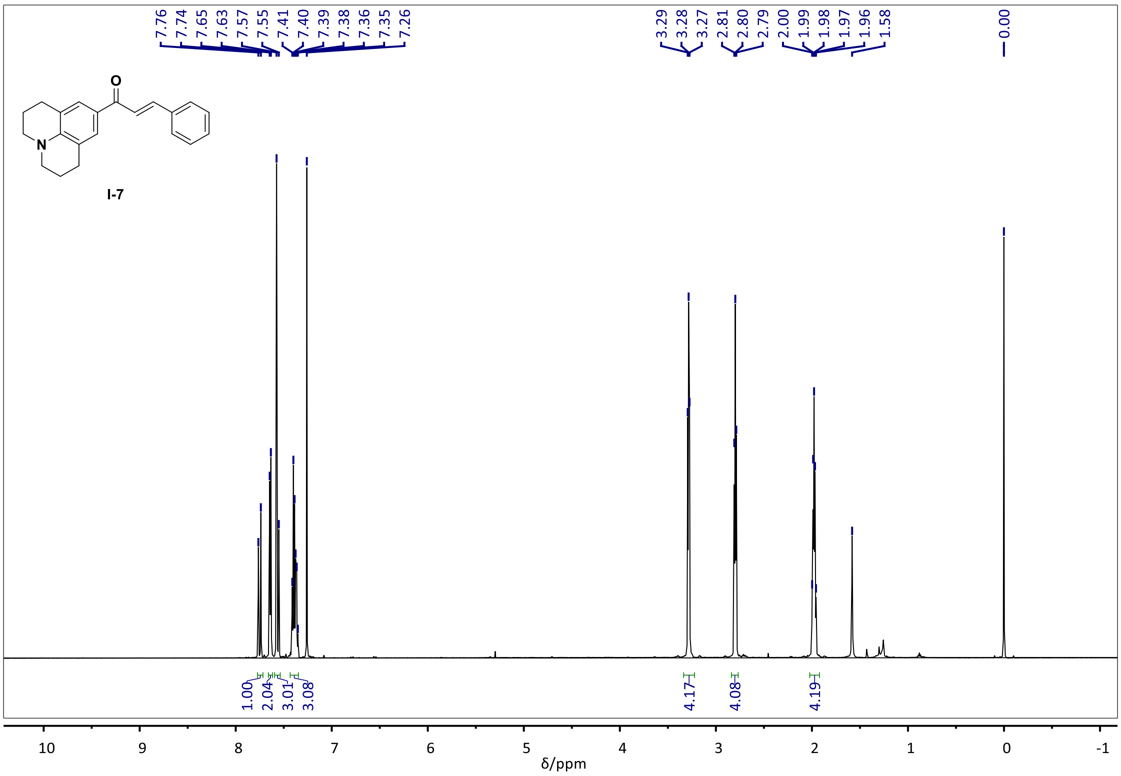

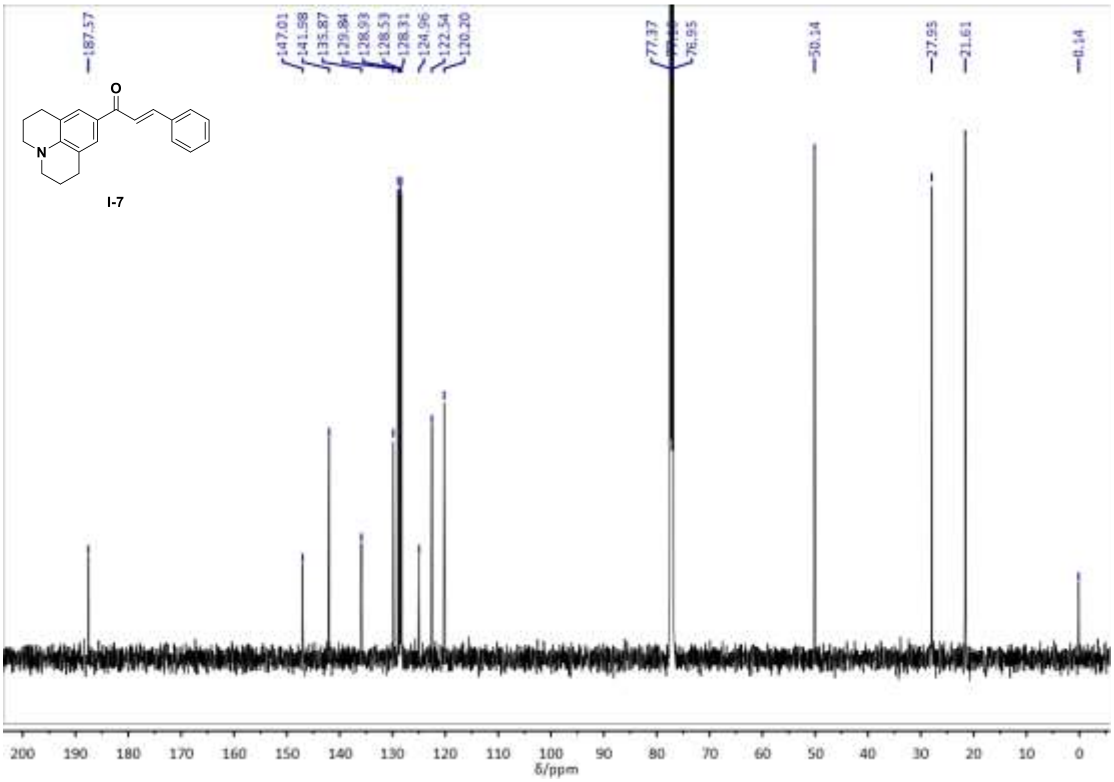

Figure S38 ^{1}H NMR (600 MHz, $CDCl_3$) and ^{13}C NMR (151 MHz, $CDCl_3$) spectra of I-7.

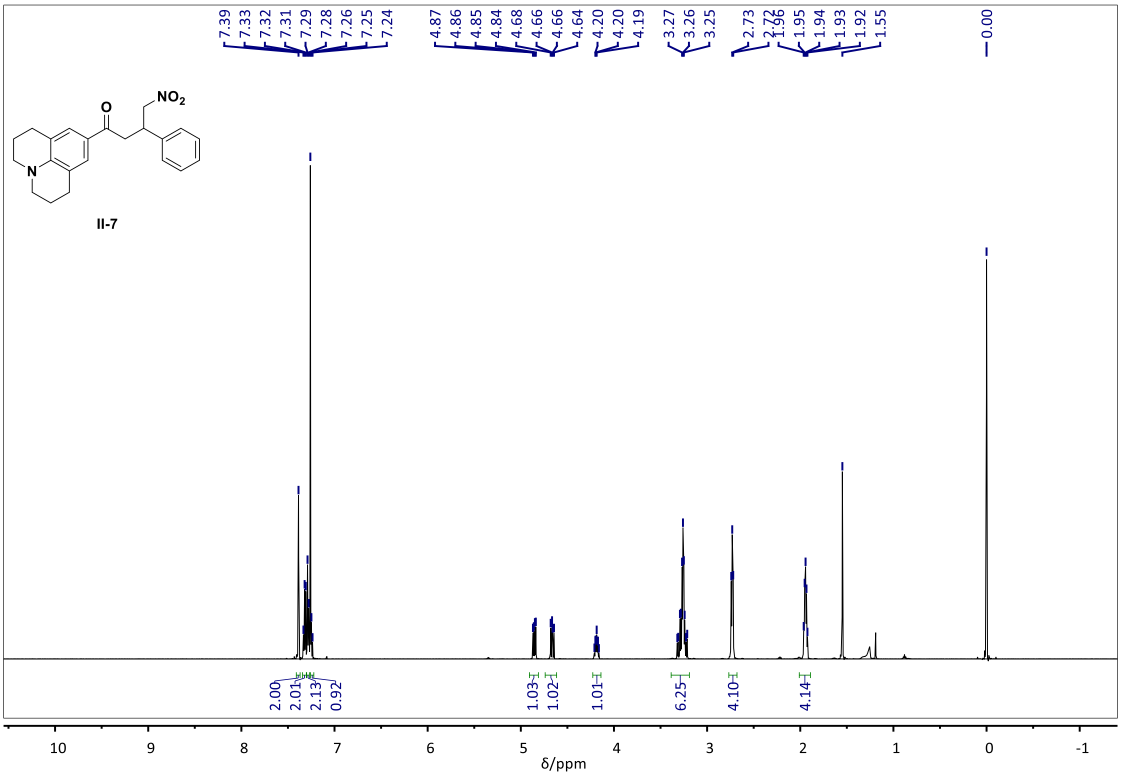

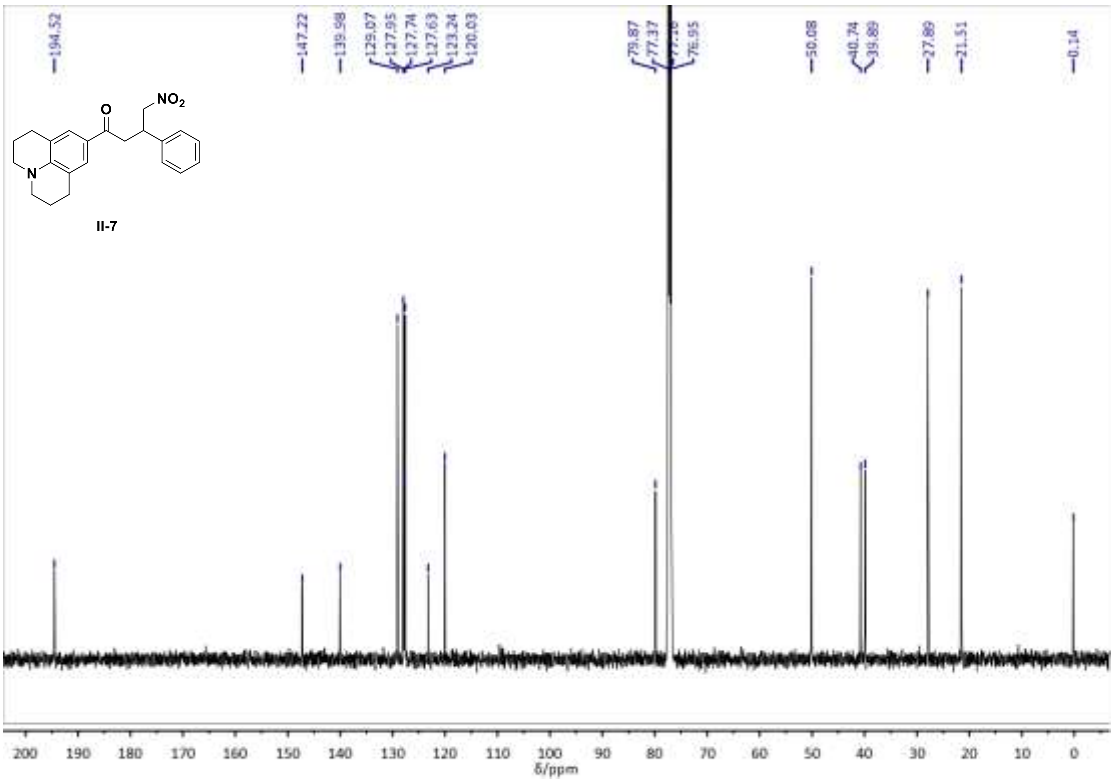

Figure S39 ^{1}H NMR (600 MHz, $CDCl_3$) and ^{13}C NMR (151 MHz, $CDCl_3$) spectra of II-7.

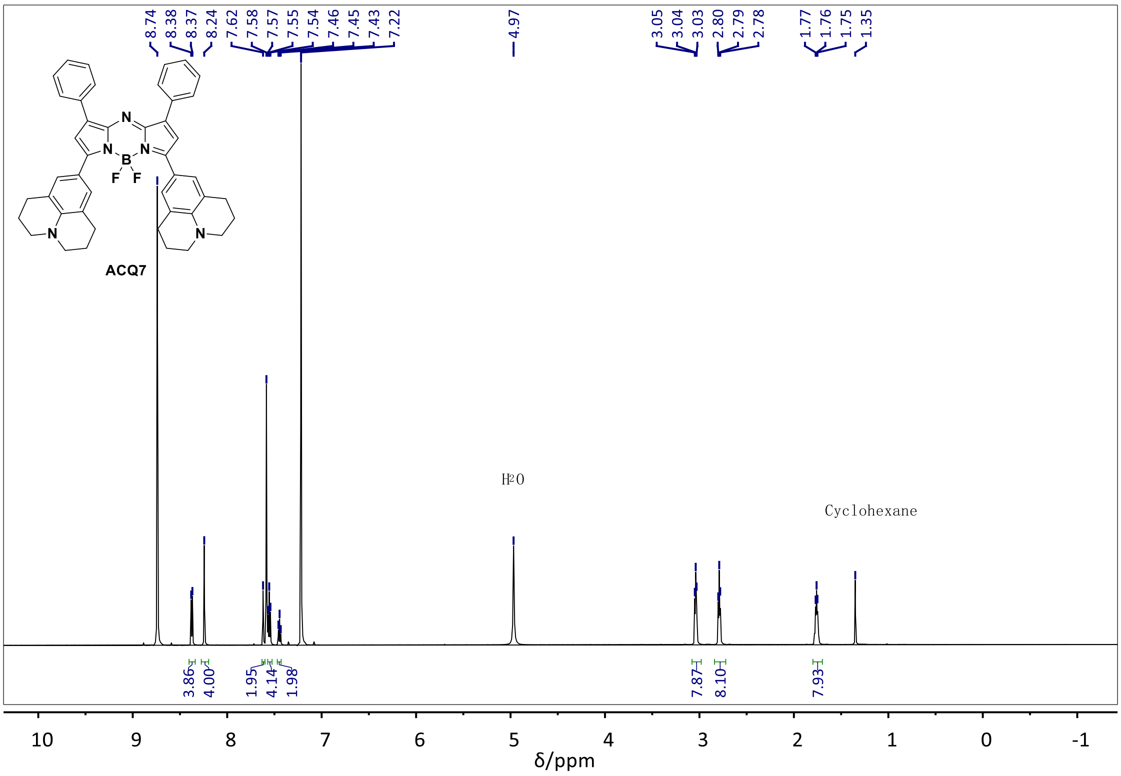

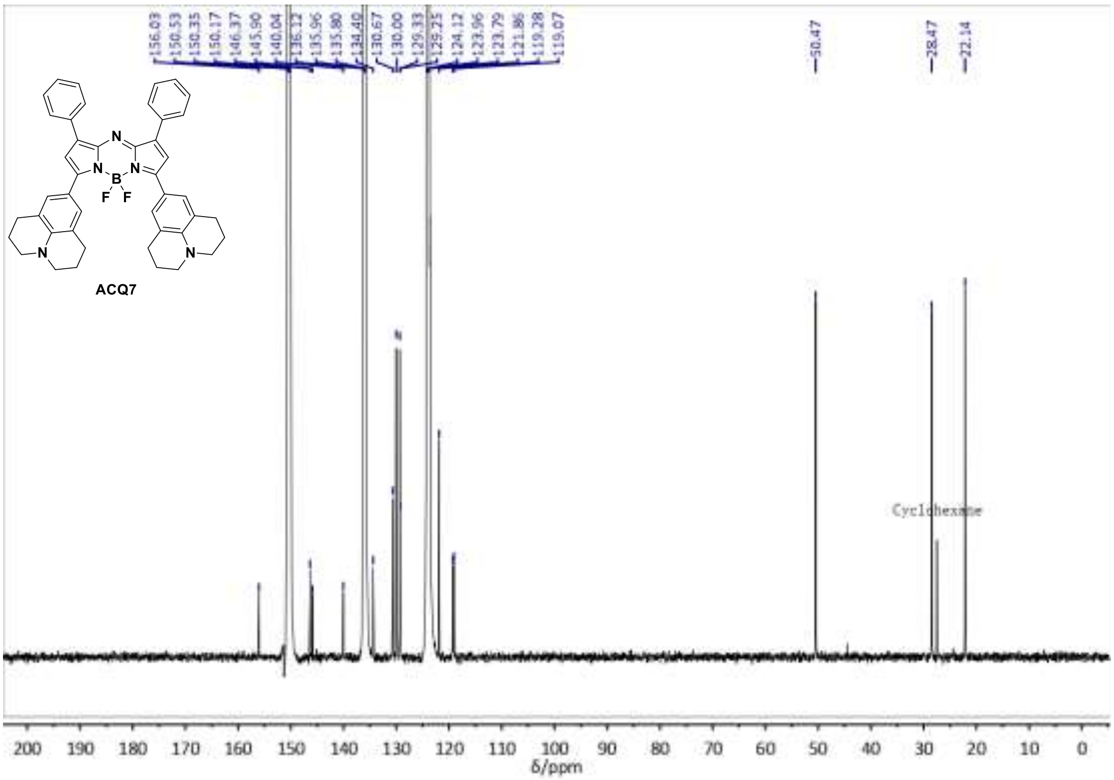

Figure S40 ^{1}H NMR (600 MHz, pyridine-d_5) and ^{13}C NMR (151 MHz, pyridine-d_5) spectra of ACQ7.

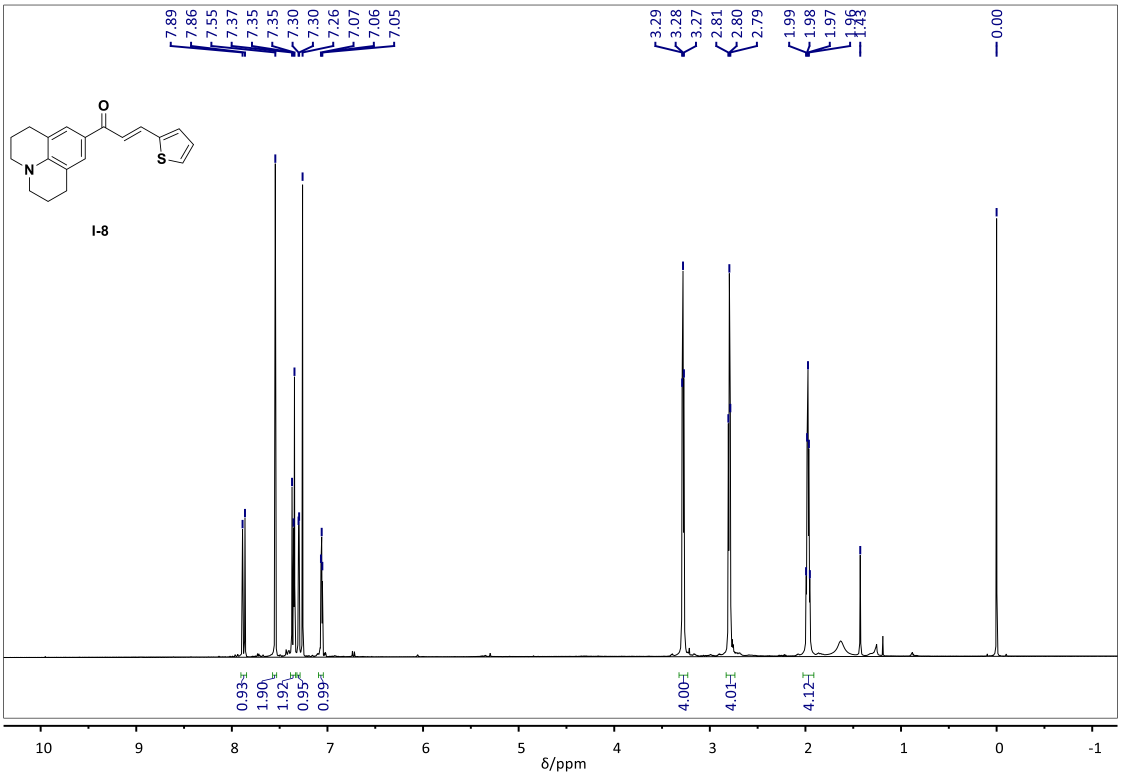

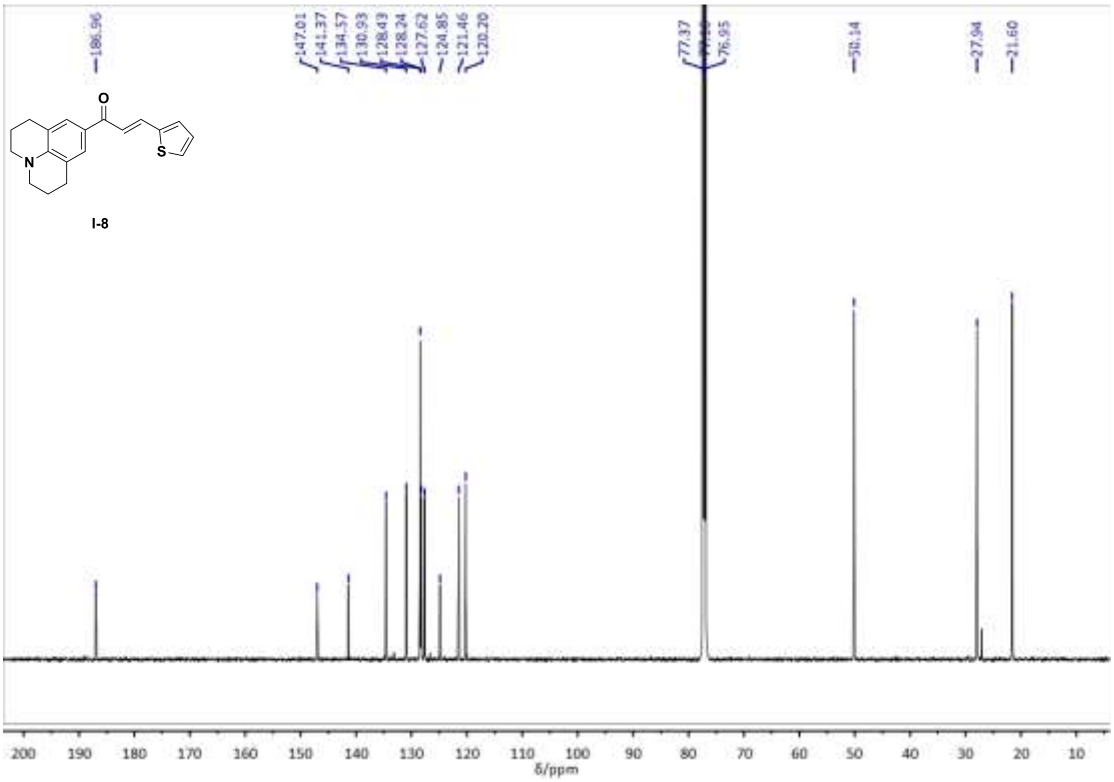

Figure S41 ^{1}H NMR (600 MHz, $CDCl_3$) and ^{13}C NMR (151 MHz, $CDCl_3$) spectra of I-8.

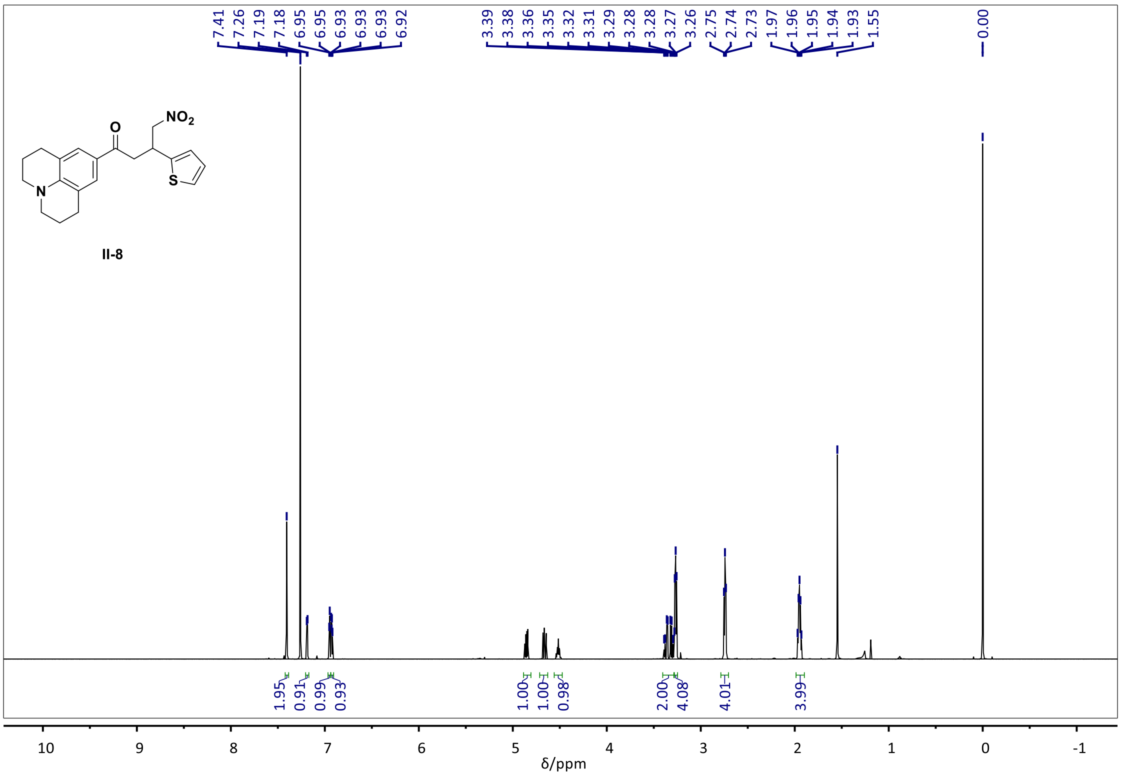

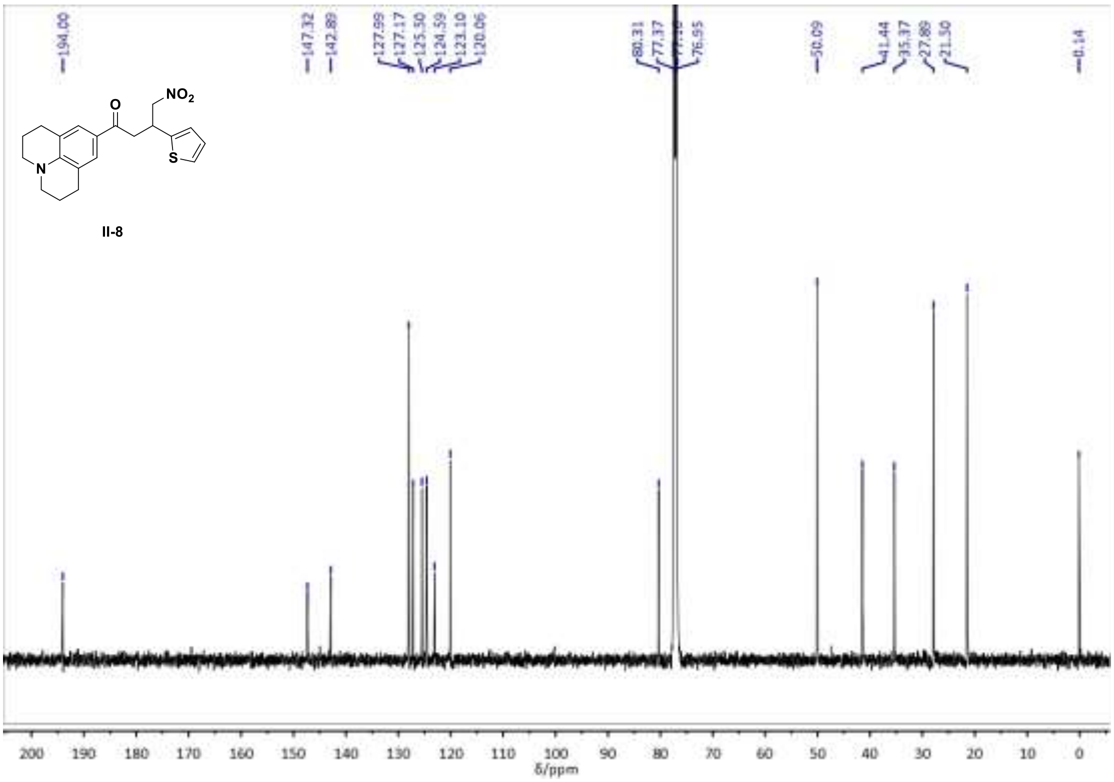

Figure S42 ^{1}H NMR (600 MHz, $CDCl_3$) and ^{13}C NMR (151 MHz, $CDCl_3$) spectra of II-8.

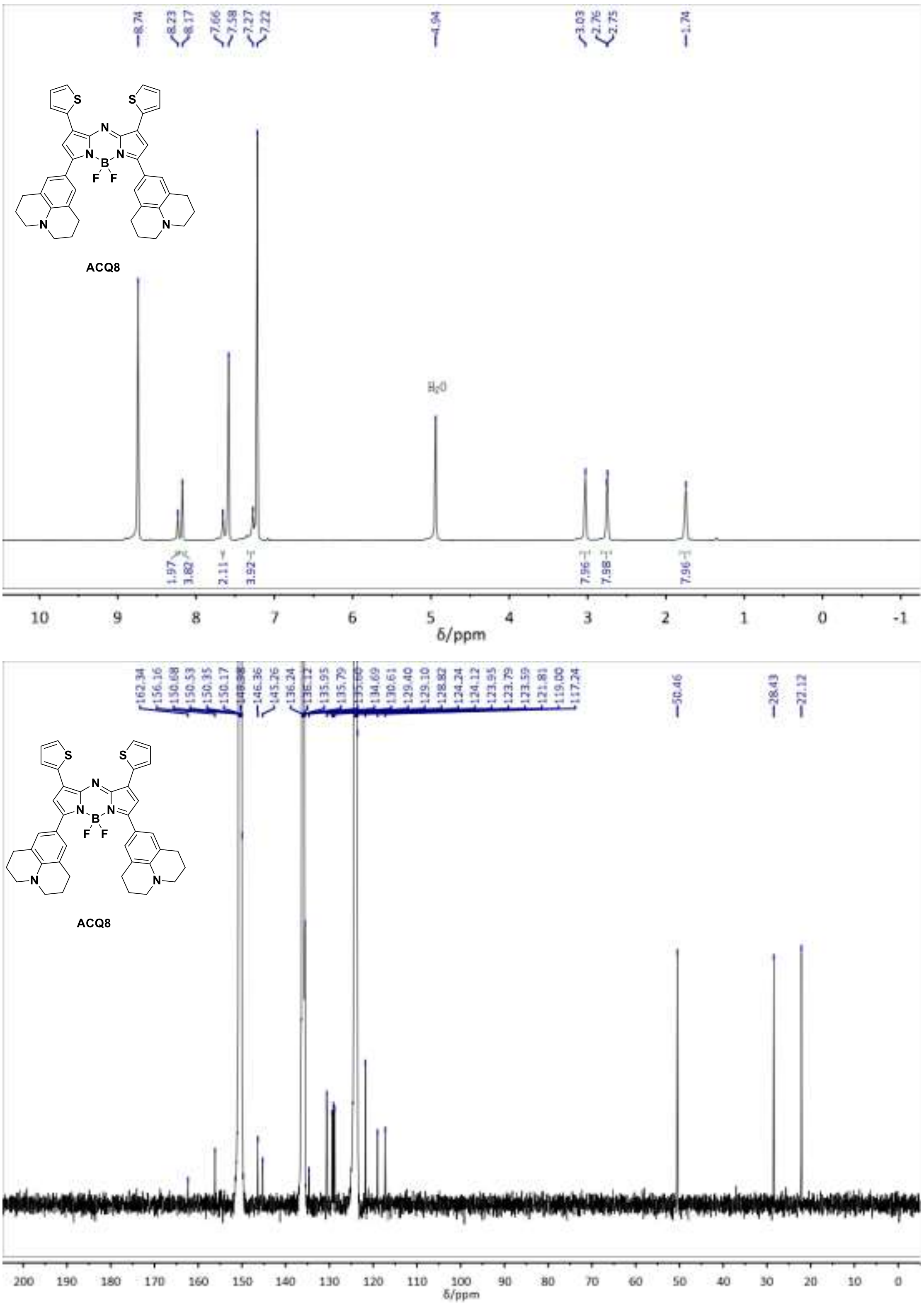

Figure S43 ^{1}H NMR (600 MHz, pyridine-d_5) and ^{13}C NMR (151 MHz, pyridine-d_5) spectra of ACQ8.

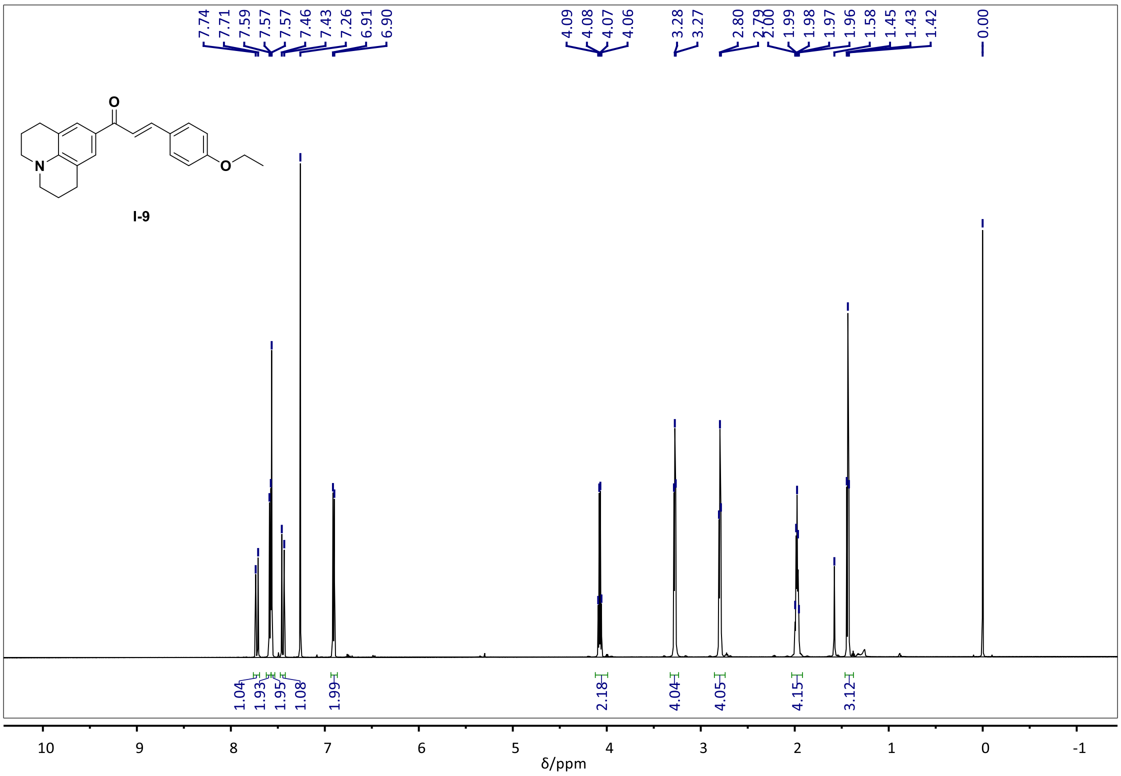
7.74
7.71
7.59
7.57
7.57
7.46
7.43
7.26
6.91
6.90
4.09
4.08
4.07
4.06
3.28
3.27
2.80
2.79
2.00
1.99
1.98
1.97
1.96
1.58
1.45
1.43
1.42
0.00
O
N
O
I-9
1.04
1.93
1.95
1.08
1.99
2.18
4.04
4.05
4.15
3.12
10
9
8
7
6
5
4
3
2
1
0
-1
δ/ppm

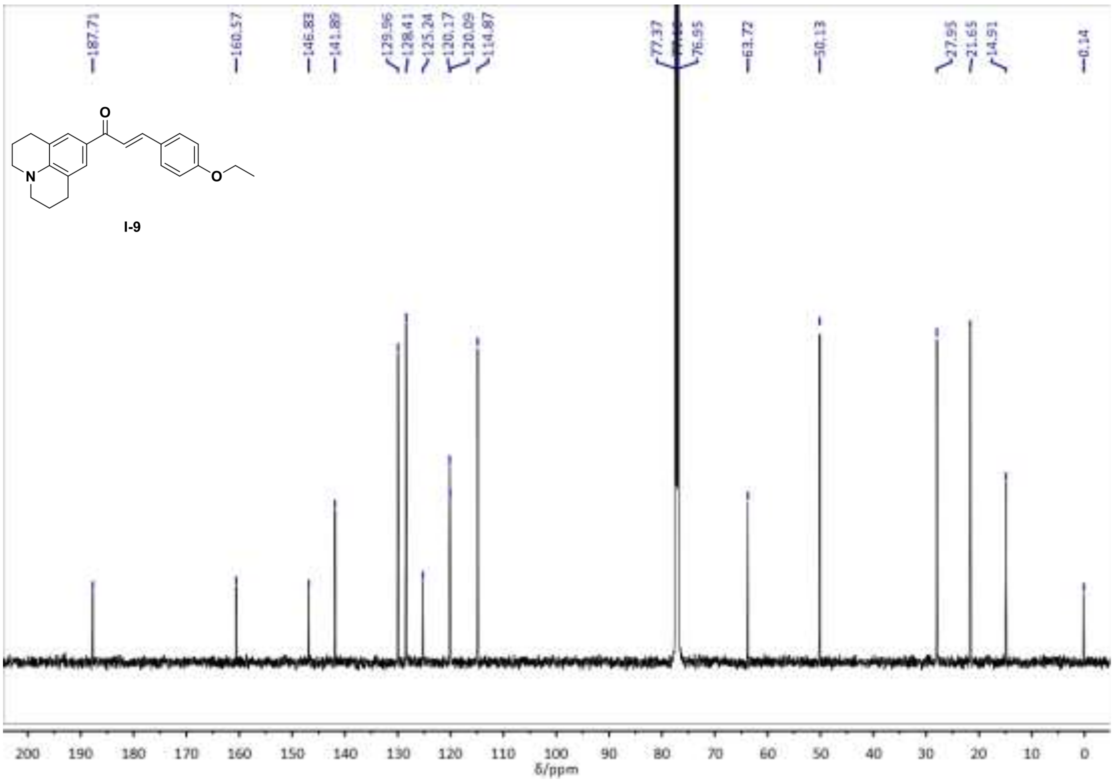
187.71
160.57
146.83
141.89
129.96
128.41
125.24
120.17
120.09
114.87
77.37
76.95
63.72
50.13
27.95
21.65
14.91
0.14
O
N
O
I-9
200
190
180
170
160
150
140
130
120
110
100
90
80
70
60
50
40
30
20
10
0
δ/ppm

Figure S44 ^{1}H NMR (600 MHz, $CDCl_3$) and ^{13}C NMR (151 MHz, $CDCl_3$) spectra of I-9.

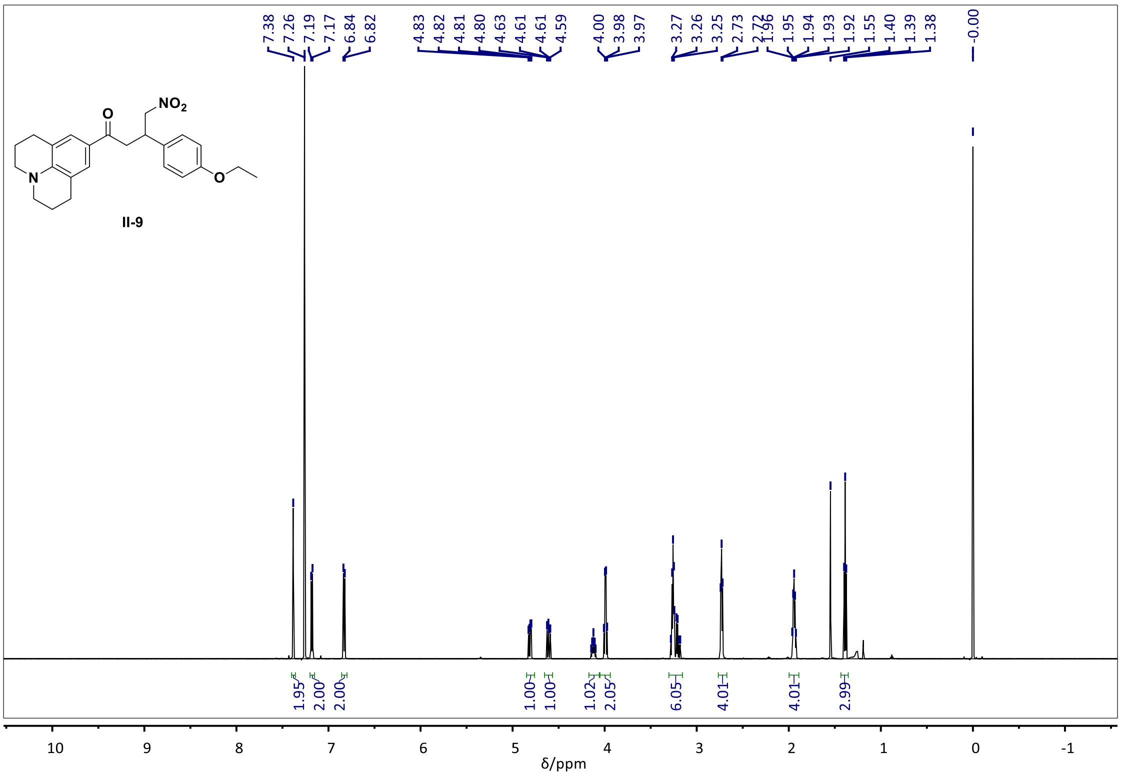

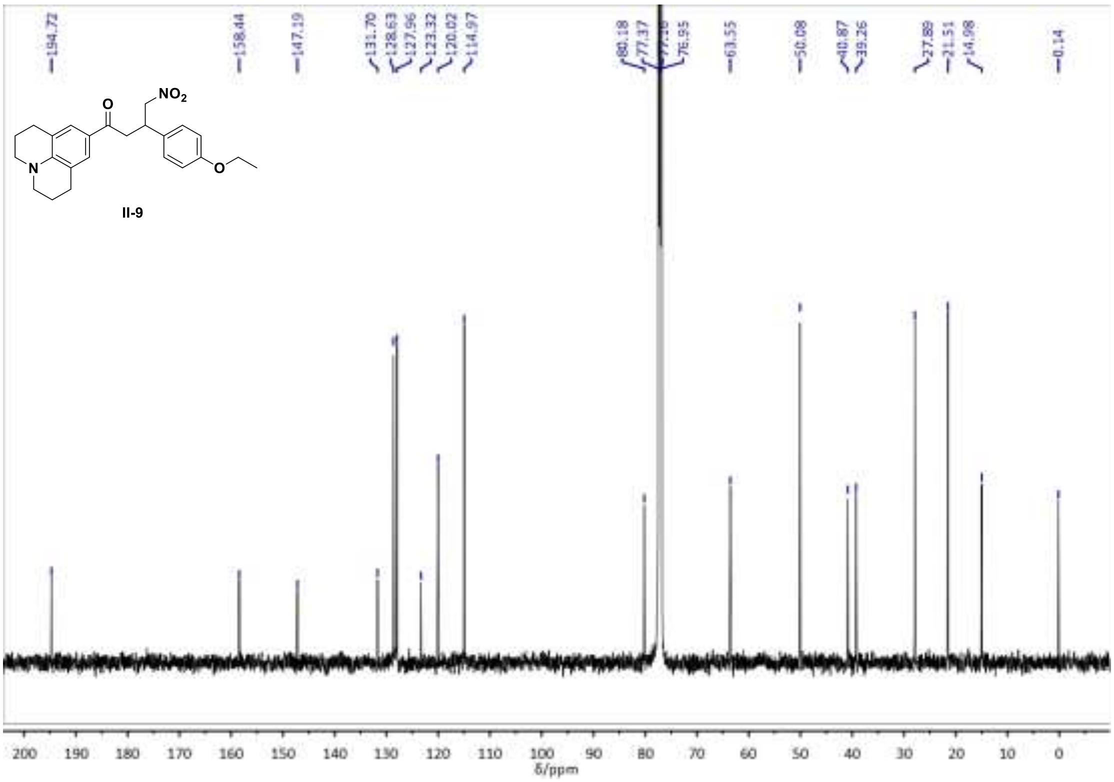

Figure S45 ^{1}H NMR (600 MHz, $CDCl_3$) and ^{13}C NMR (151 MHz, $CDCl_3$) spectra of II-9.

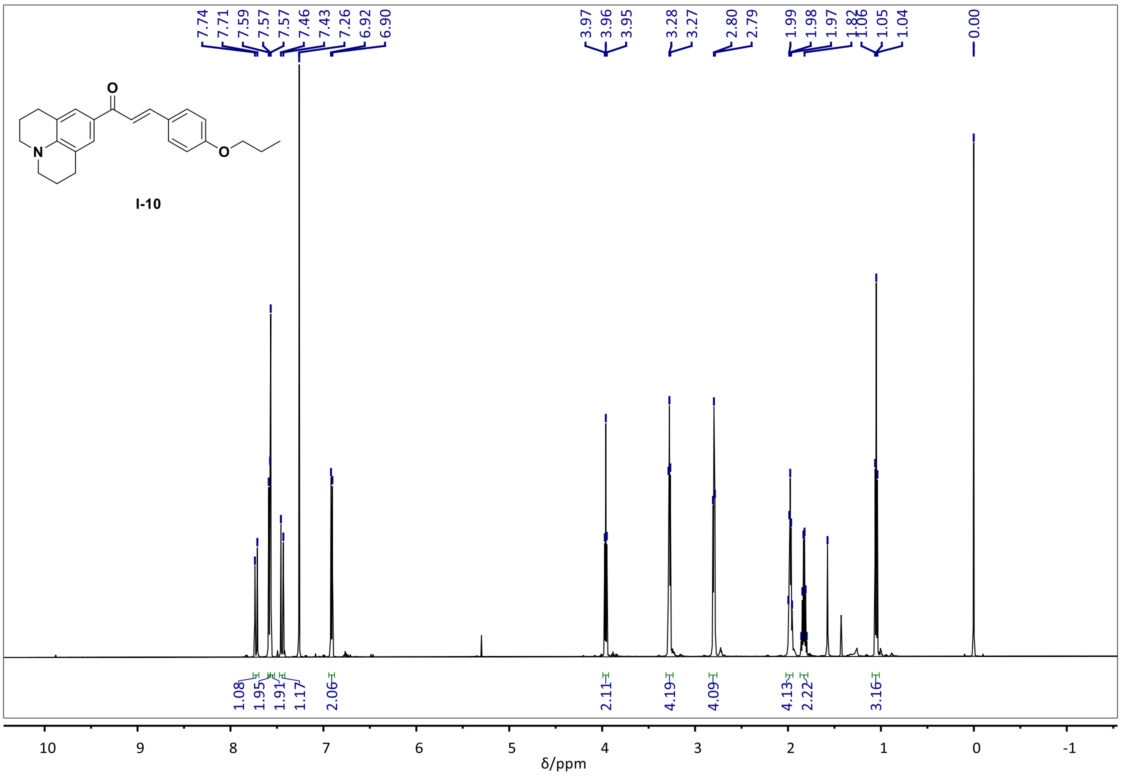

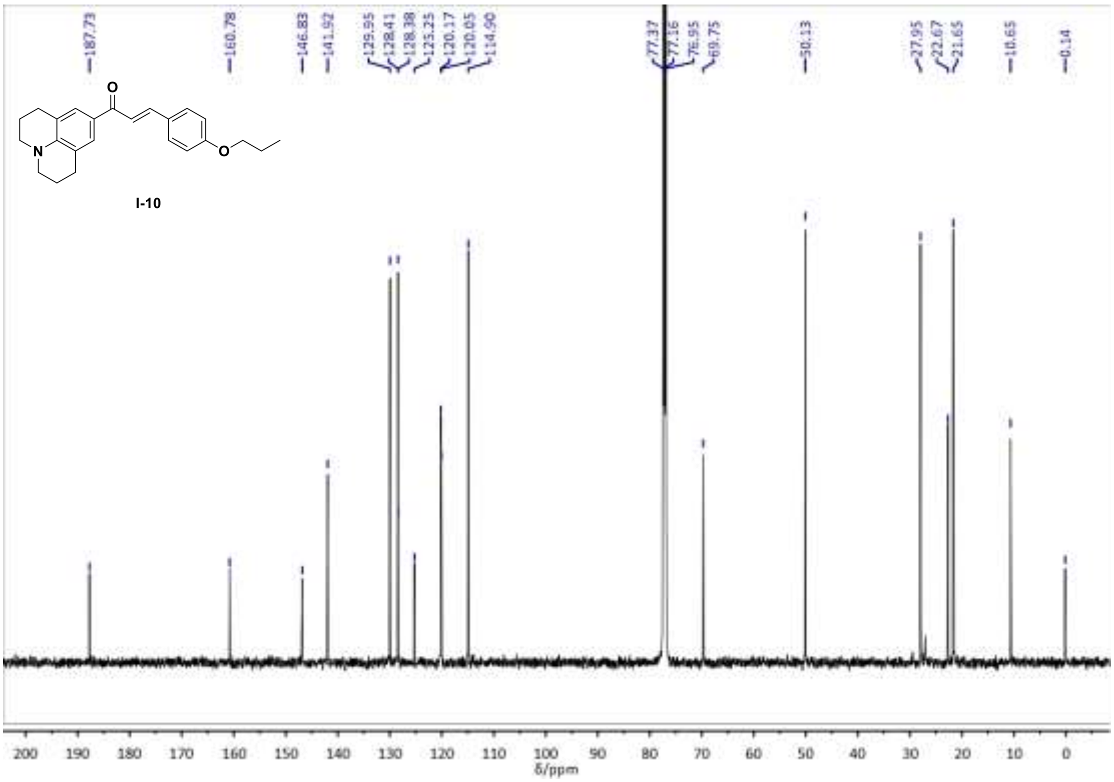

Figure S46 ^{1}H NMR (600 MHz, $CDCl_3$) and ^{13}C NMR (151 MHz, $CDCl_3$) spectra of I-10.

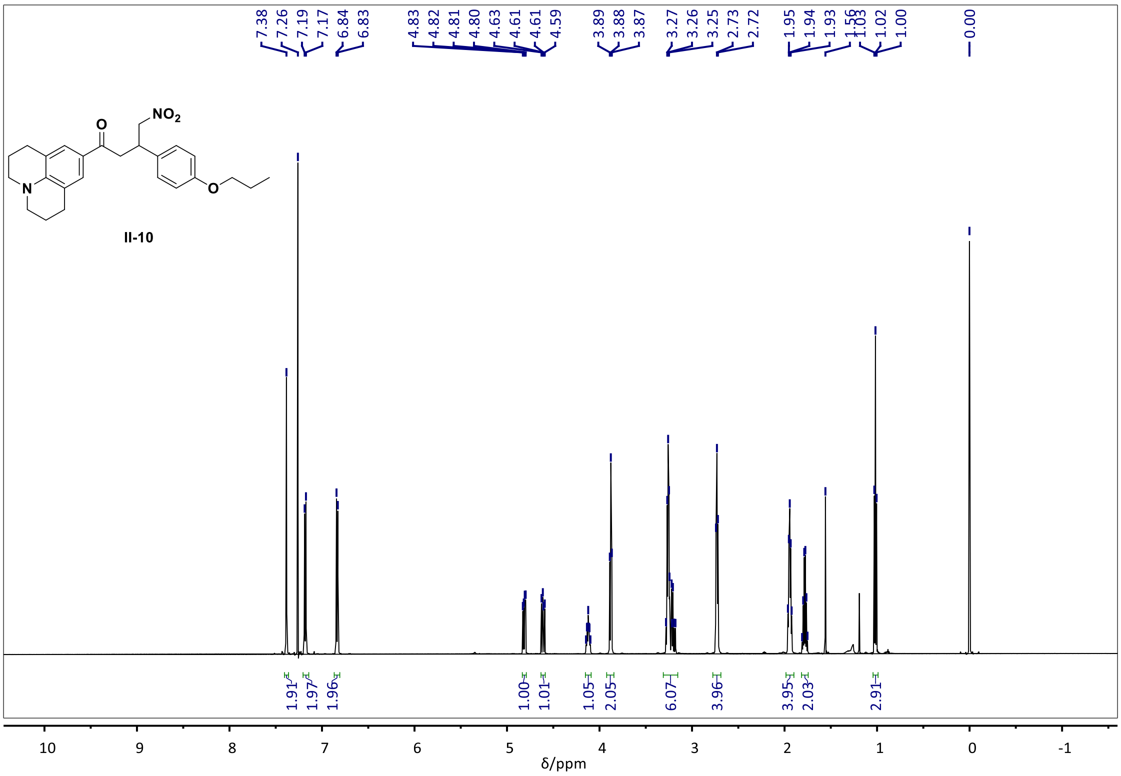

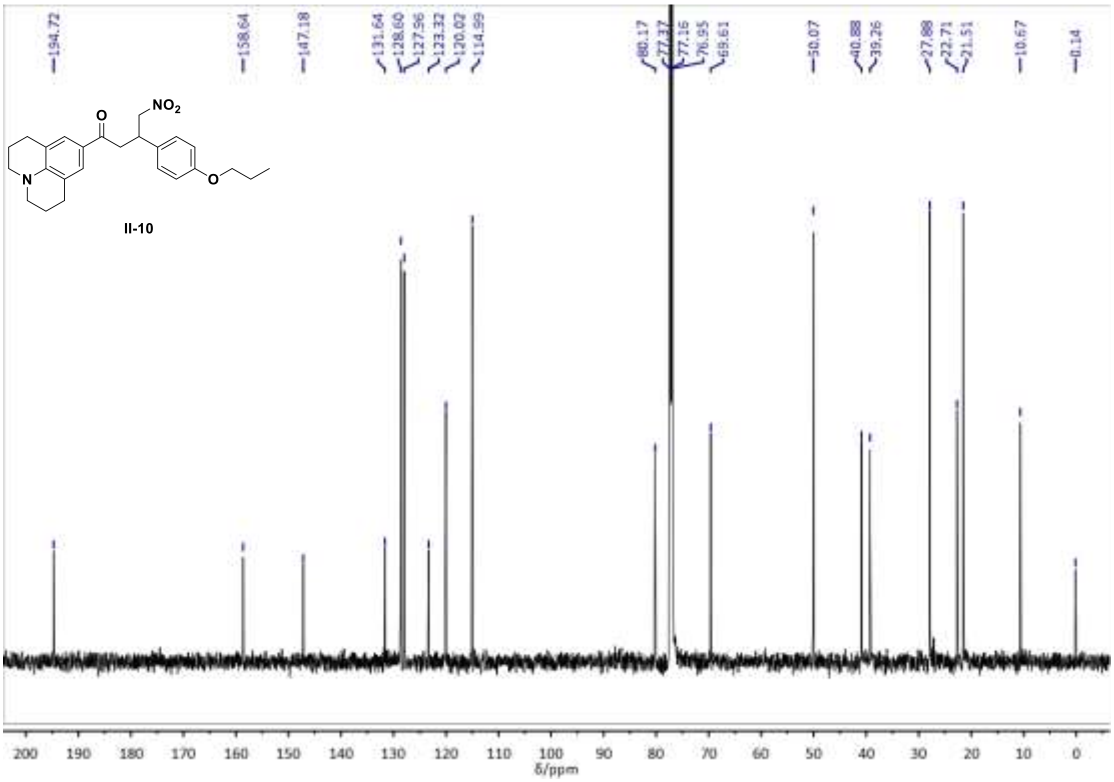

Figure S47 ^{1}H NMR (600 MHz, $CDCl_3$) and ^{13}C NMR (151 MHz, $CDCl_3$) spectra of II-10.

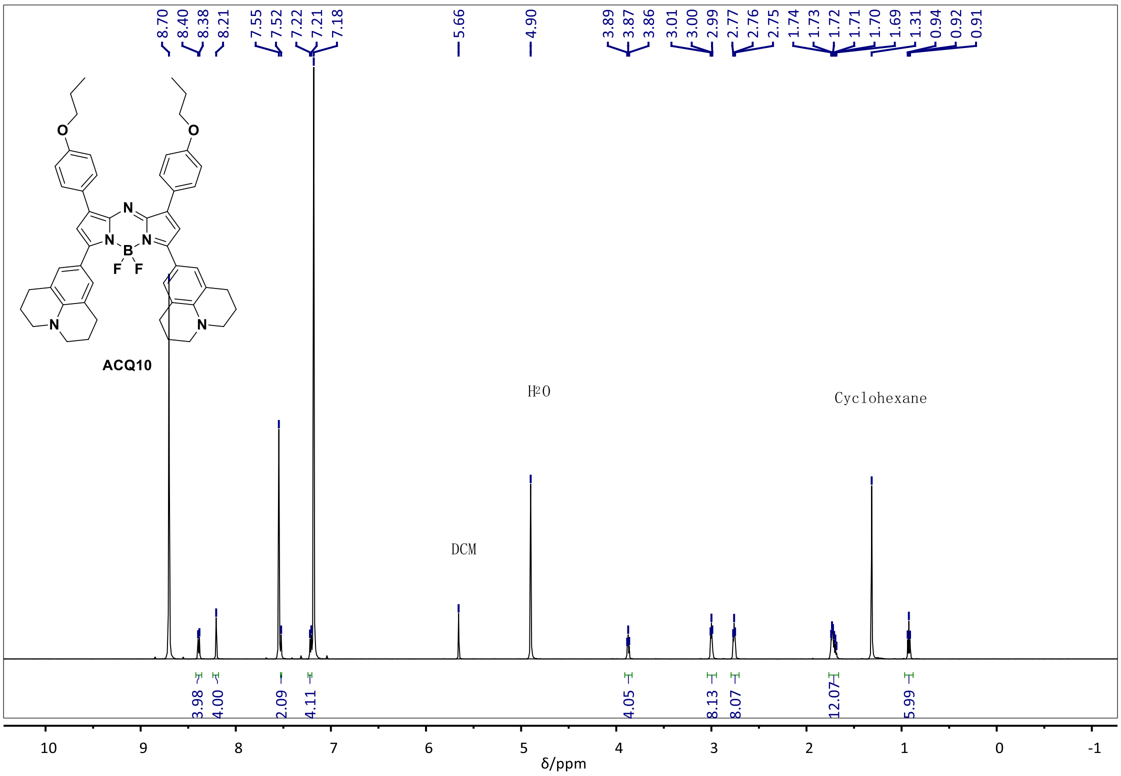

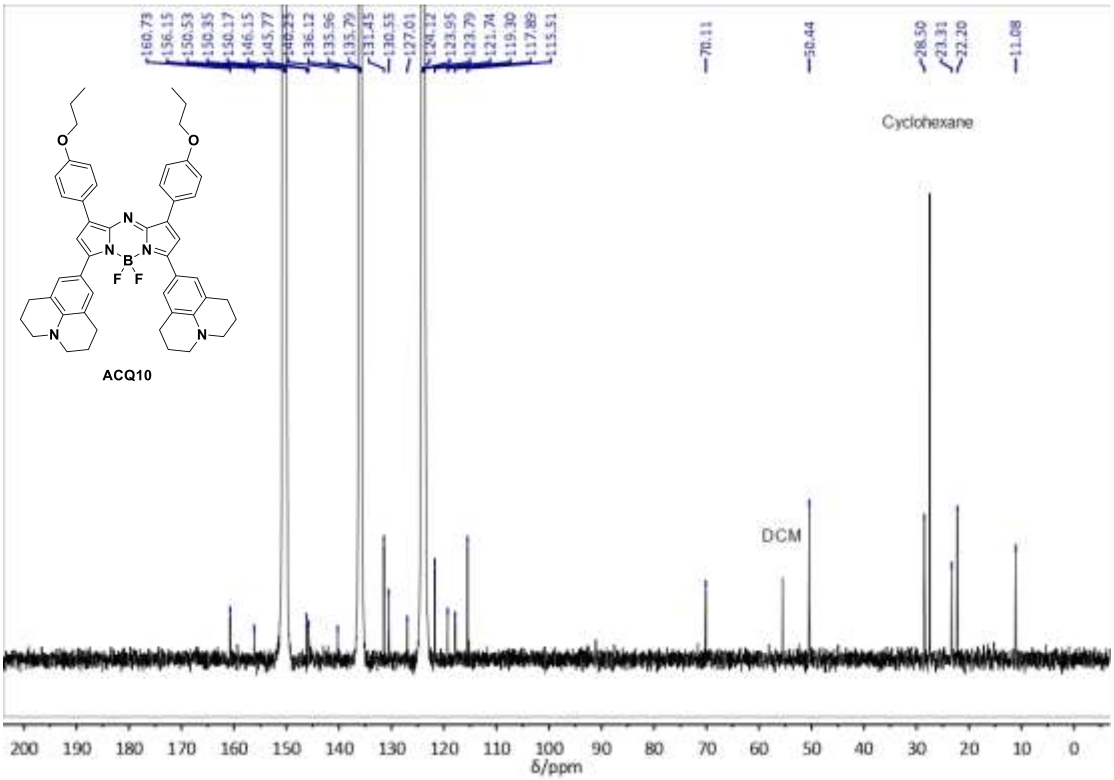

Figure S48 ^{1}H NMR (600 MHz, pyridine-d_5) and ^{13}C NMR (151 MHz, pyridine-d_5) spectra of ACQ10.

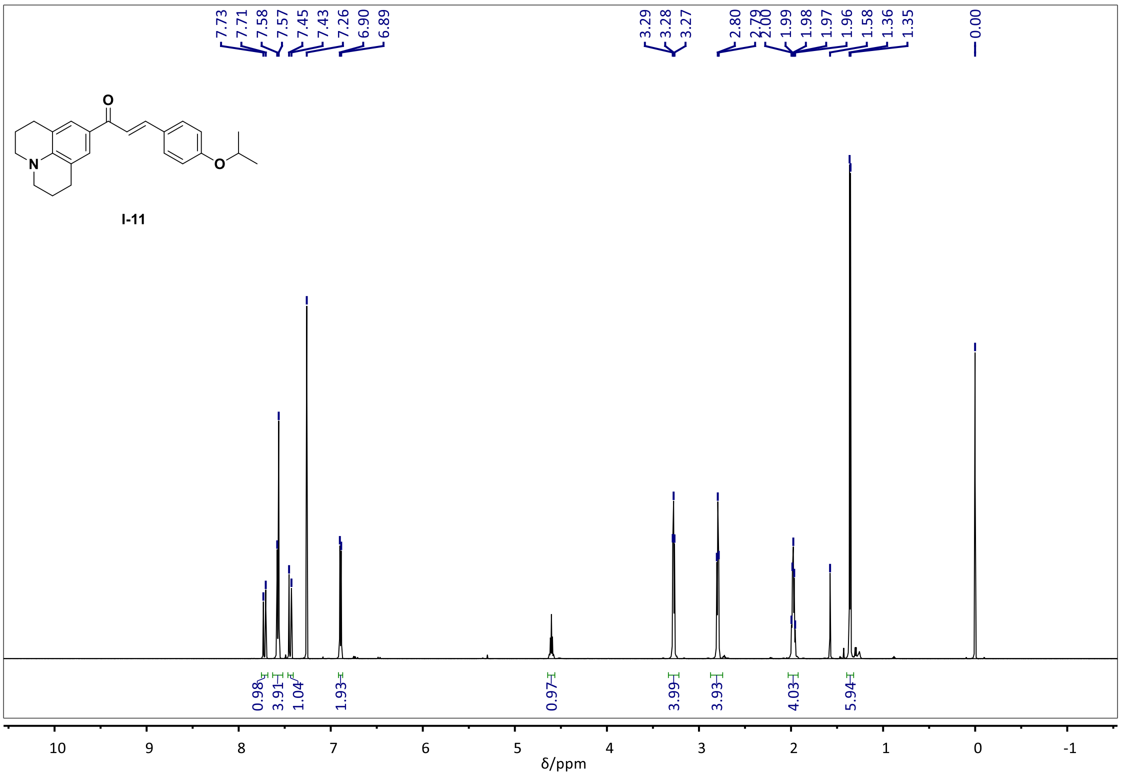

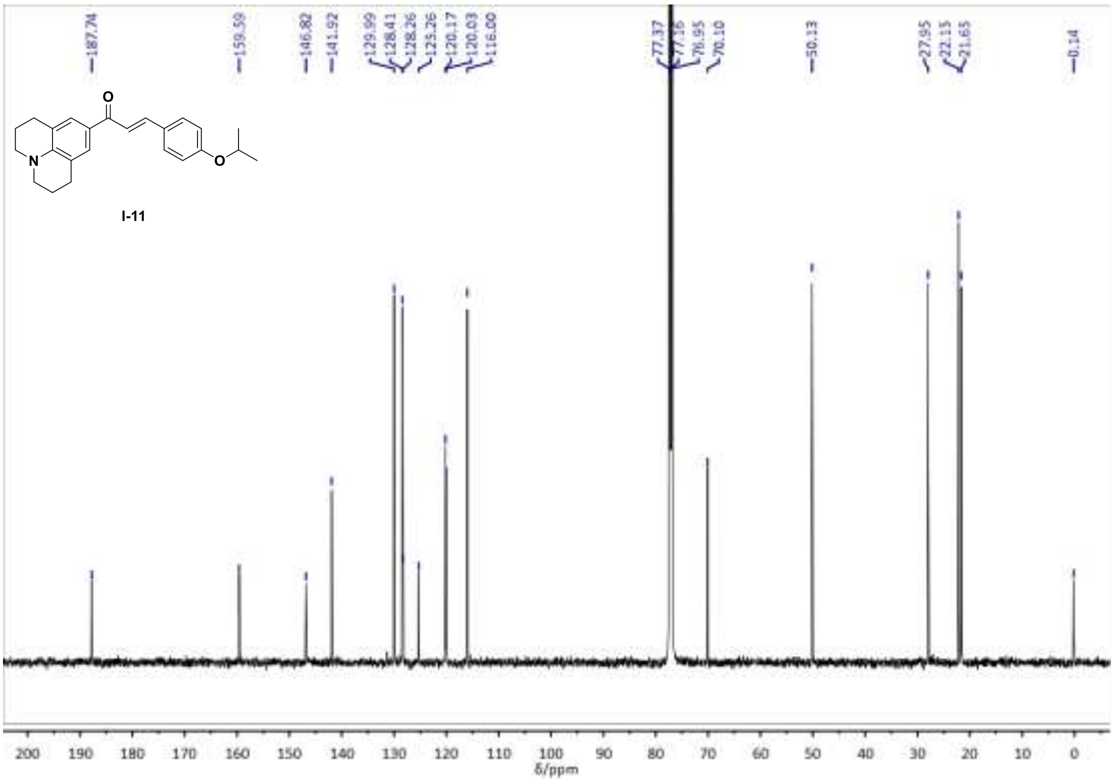

Figure S49 ^{1}H NMR (600 MHz, $CDCl_3$) and ^{13}C NMR (151 MHz, $CDCl_3$) spectra of I-11.

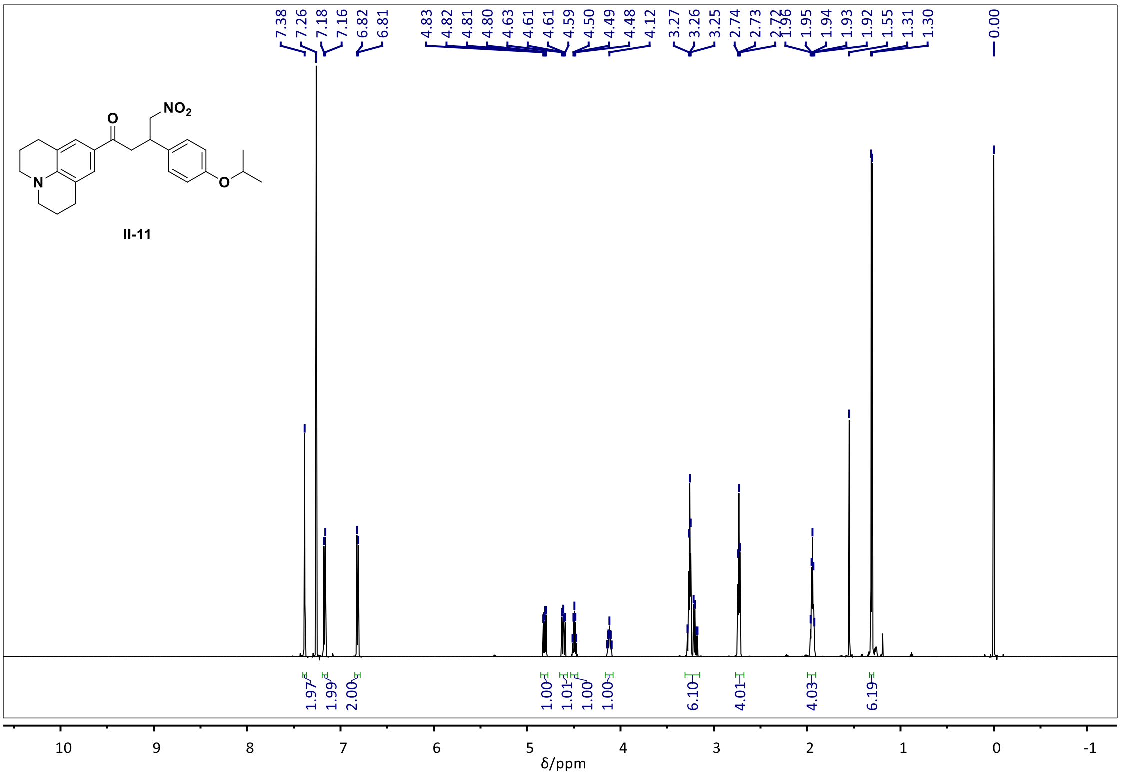

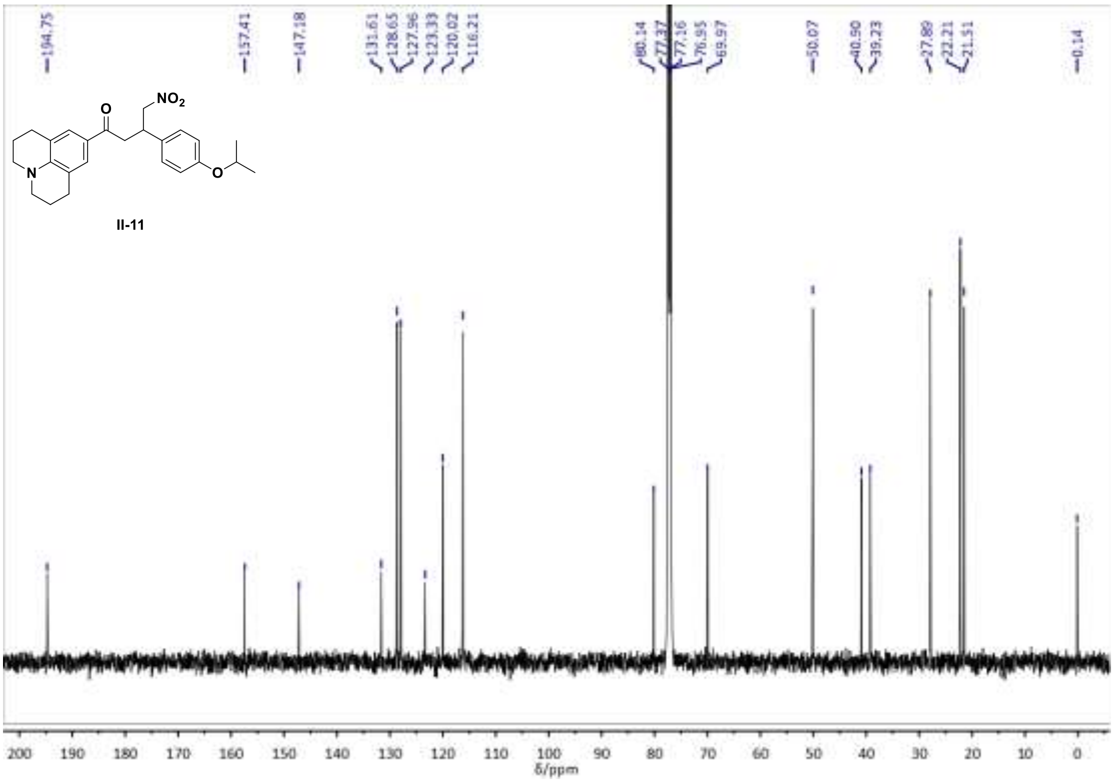

Figure S50 ^{1}H NMR (600 MHz, $CDCl_3$) and ^{13}C NMR (151 MHz, $CDCl_3$) spectra of II-11.

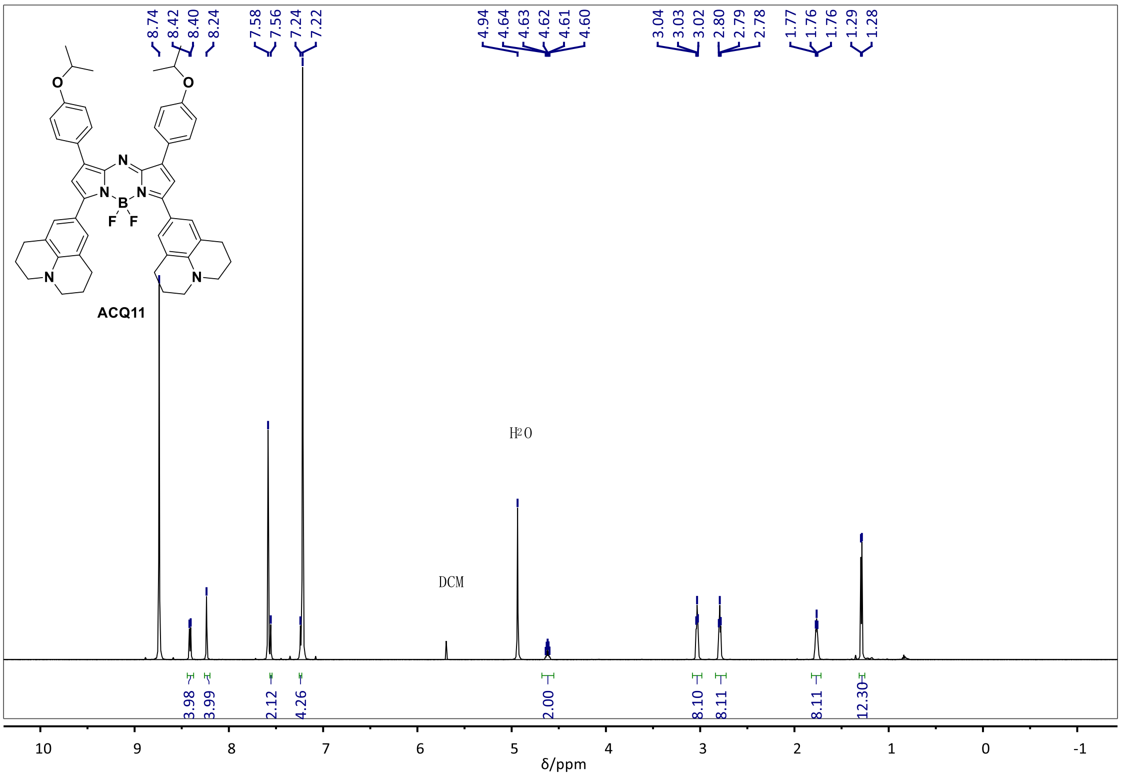

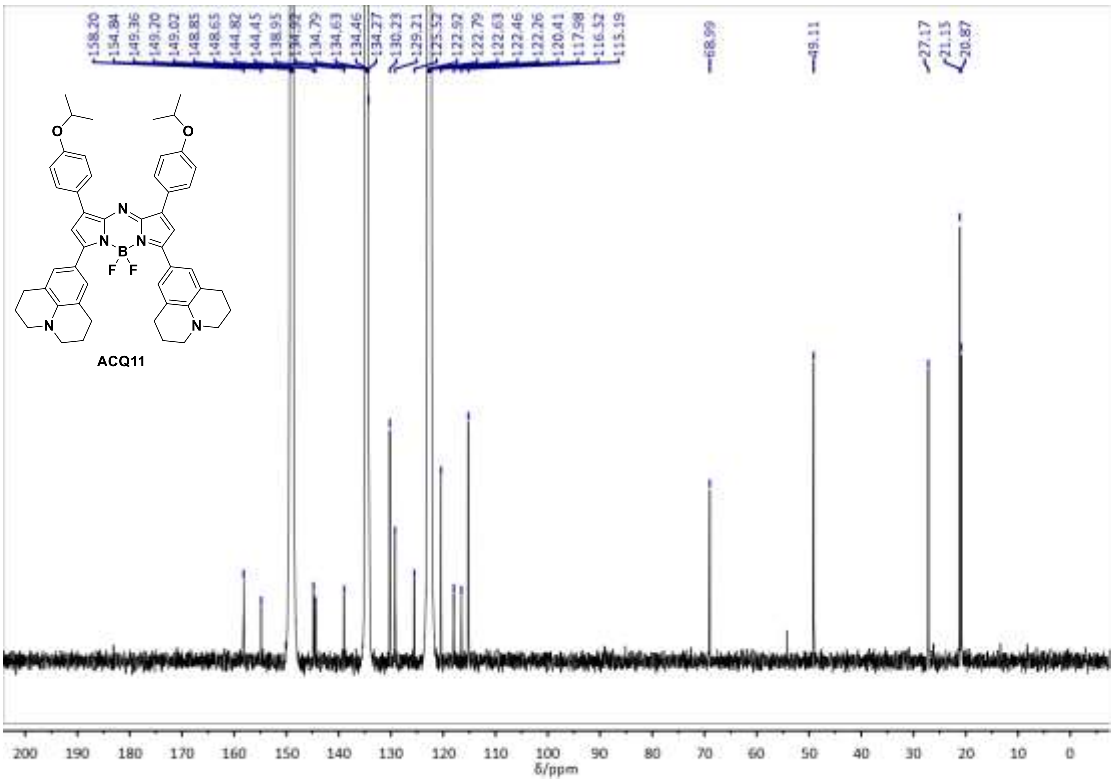

Figure S51 ^{1}H NMR (600 MHz, pyridine-d_5) and ^{13}C NMR (151 MHz, pyridine-d_5) spectra of ACQ11.

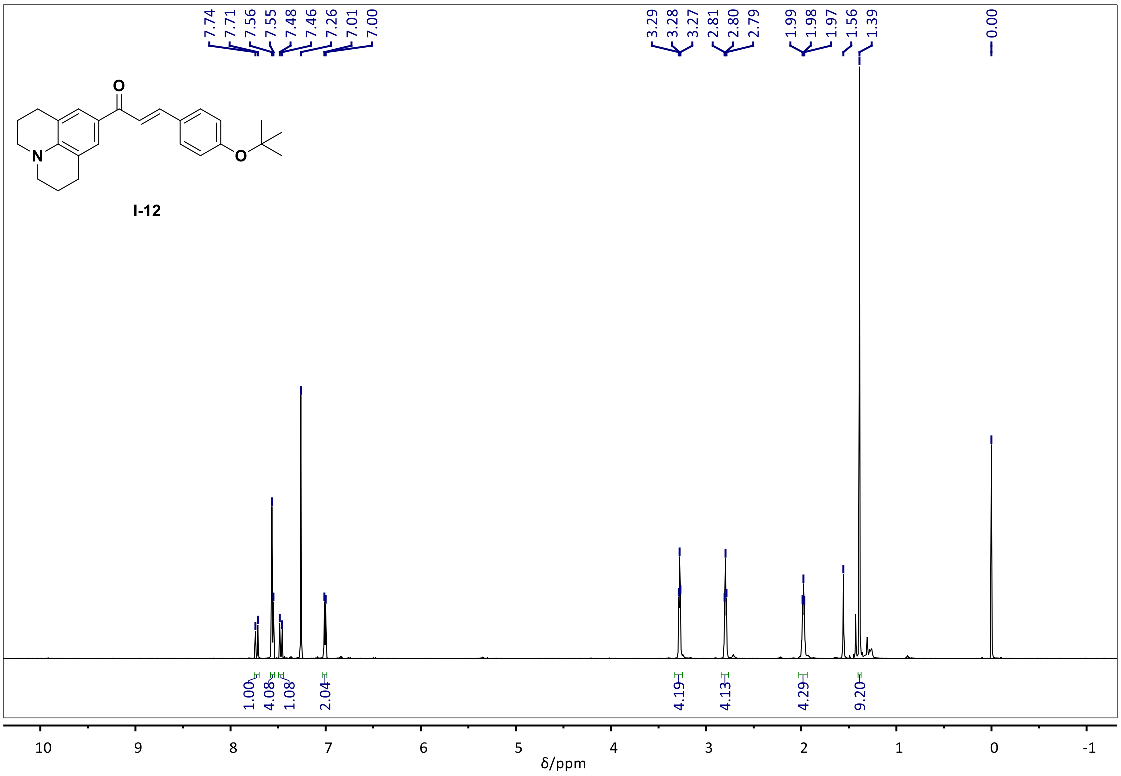

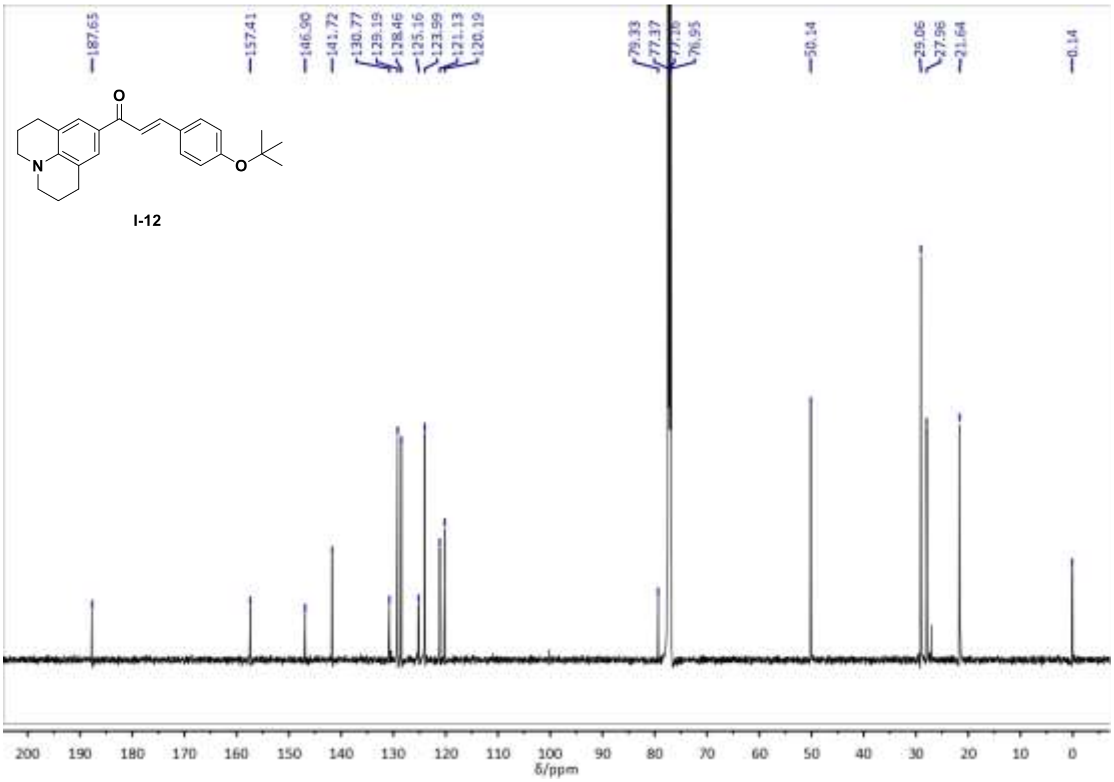

Figure S52 ^{1}H NMR (600 MHz, $CDCl_3$) and ^{13}C NMR (151 MHz, $CDCl_3$) spectra of I-12.

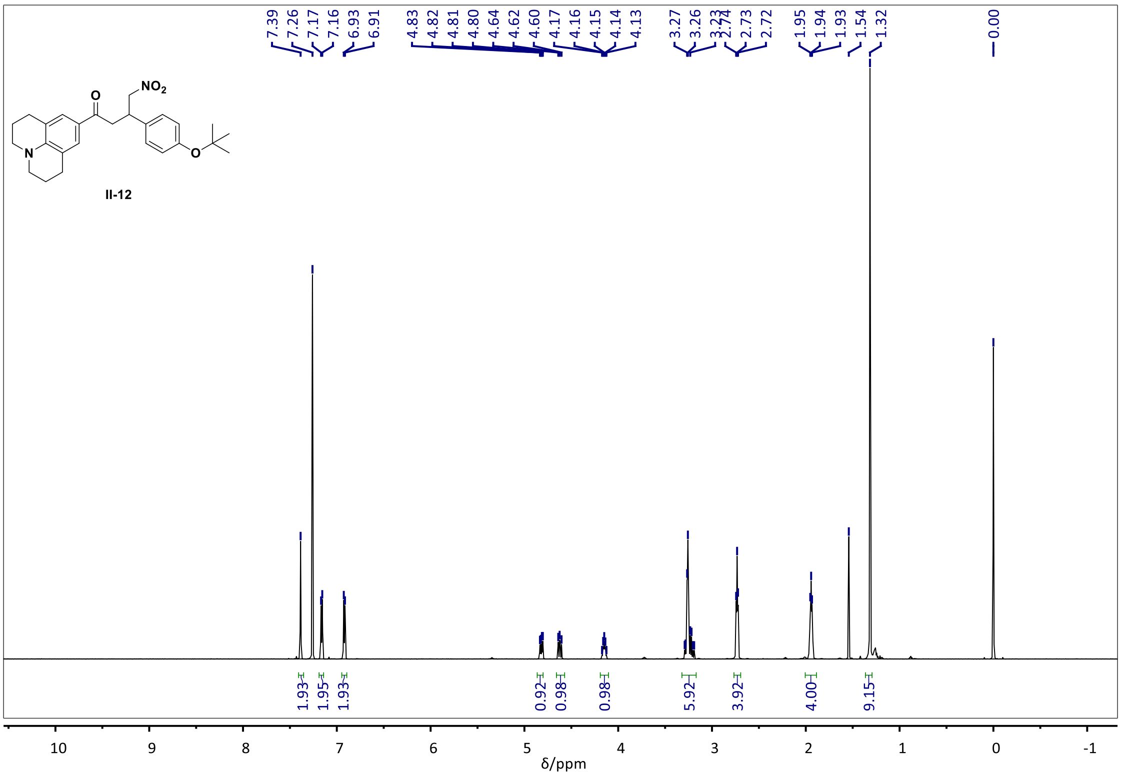

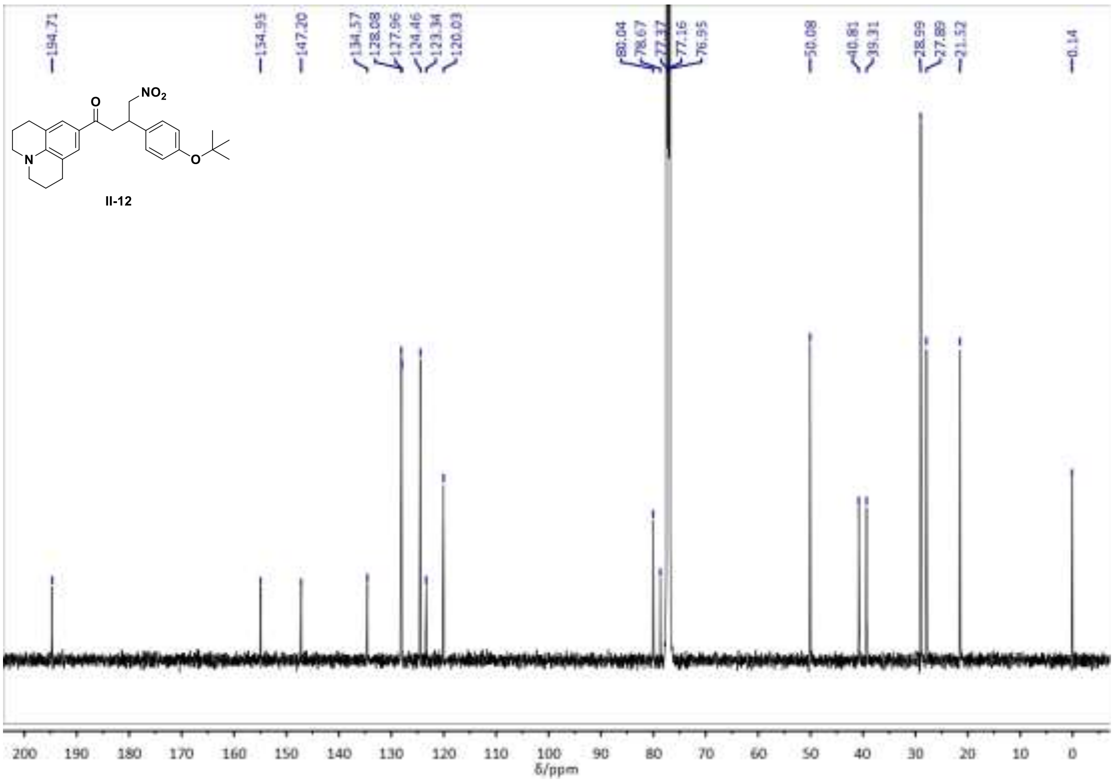

Figure S53 ^{1}H NMR (600 MHz, $CDCl_3$) and ^{13}C NMR (151 MHz, $CDCl_3$) spectra of II-12.

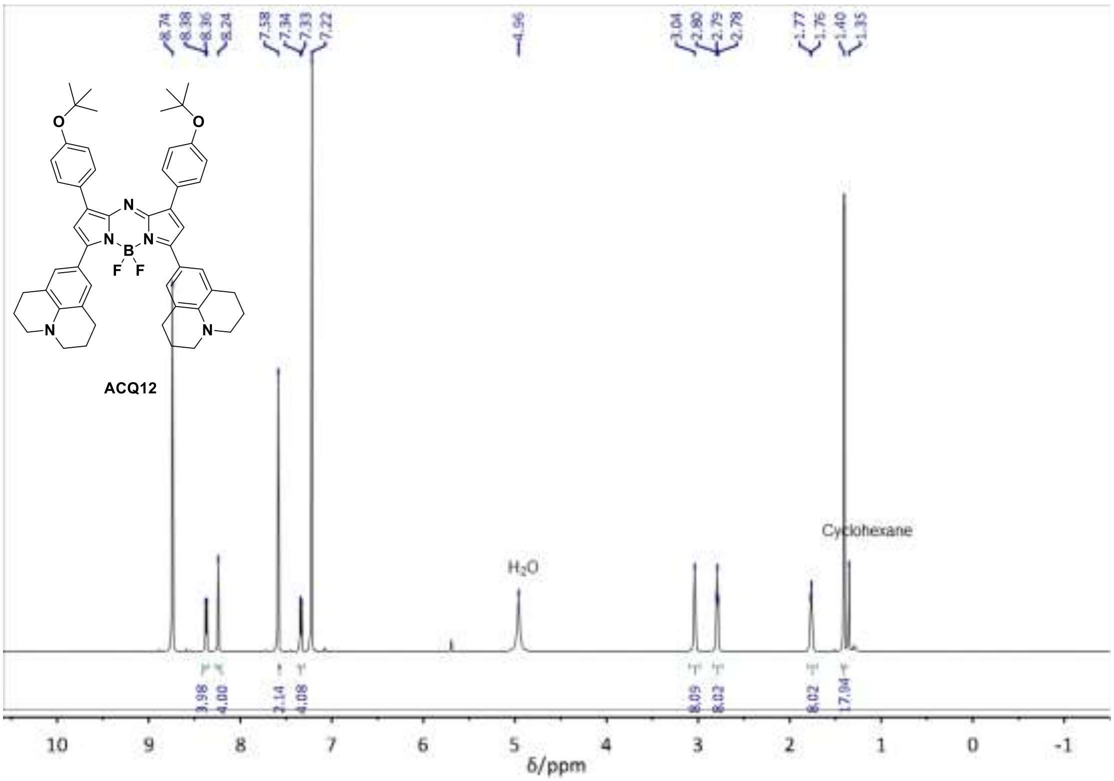

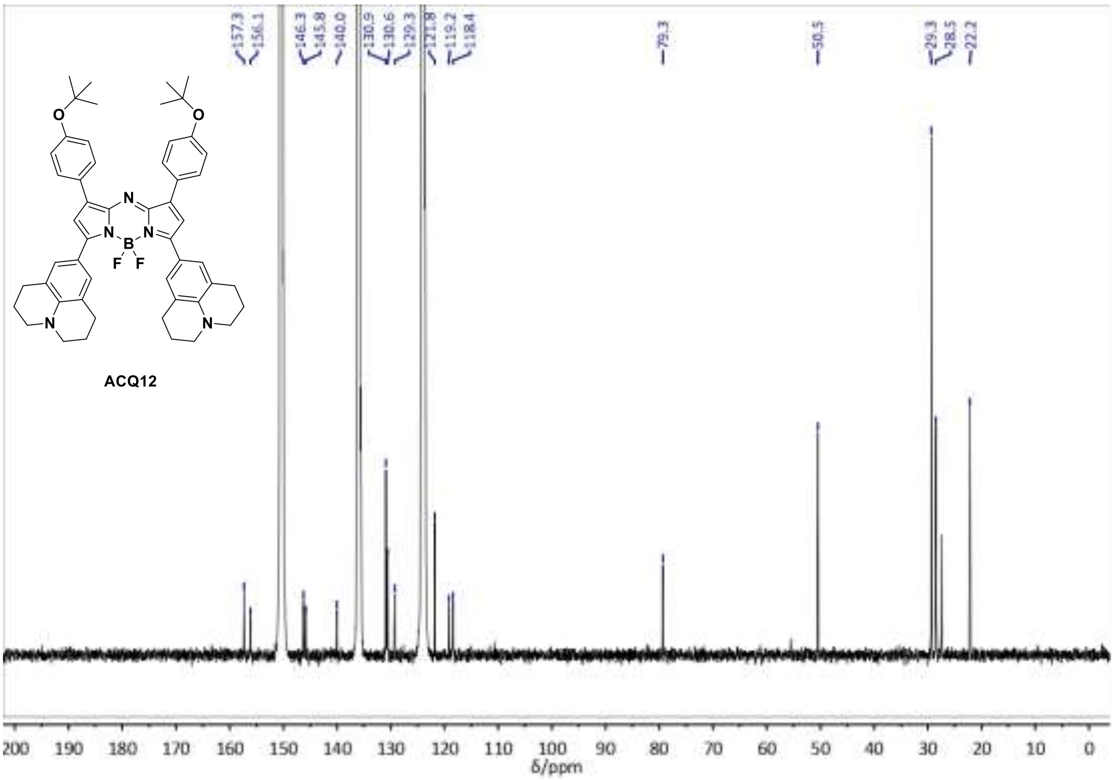

Figure S54 ^{1}H NMR (600 MHz, pyridine-d_5) and ^{13}C NMR (151 MHz, pyridine-d_5) spectra of ACQ12.

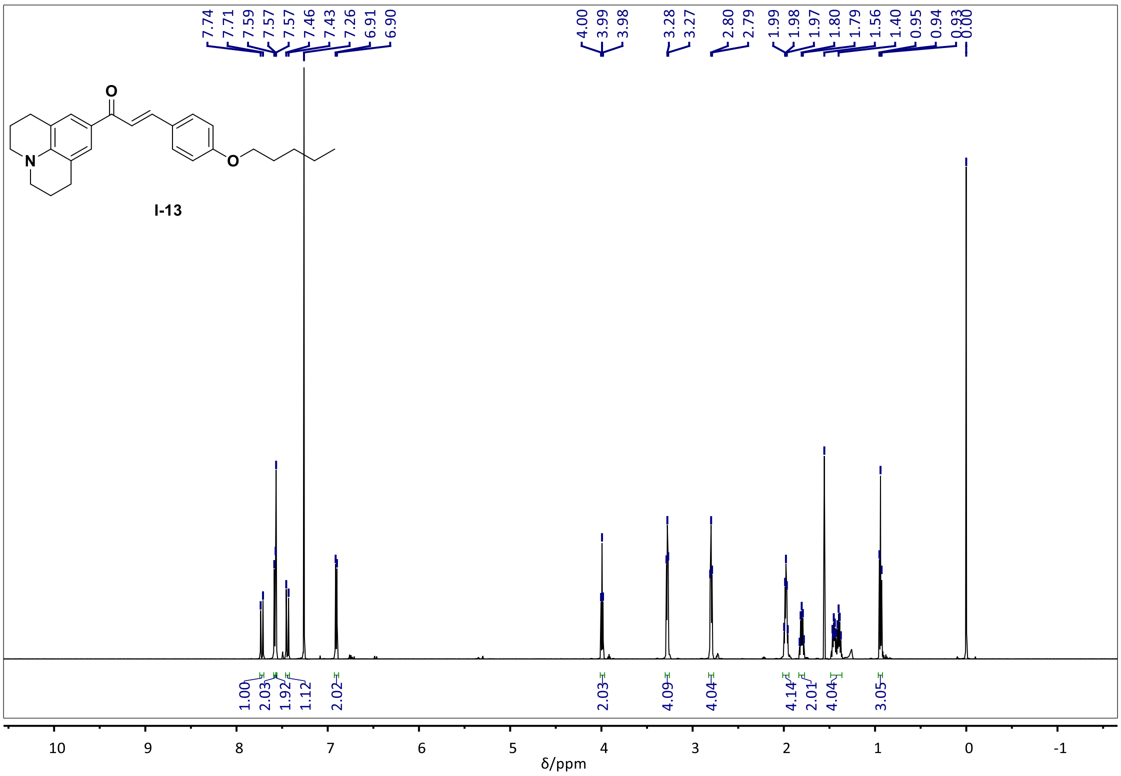

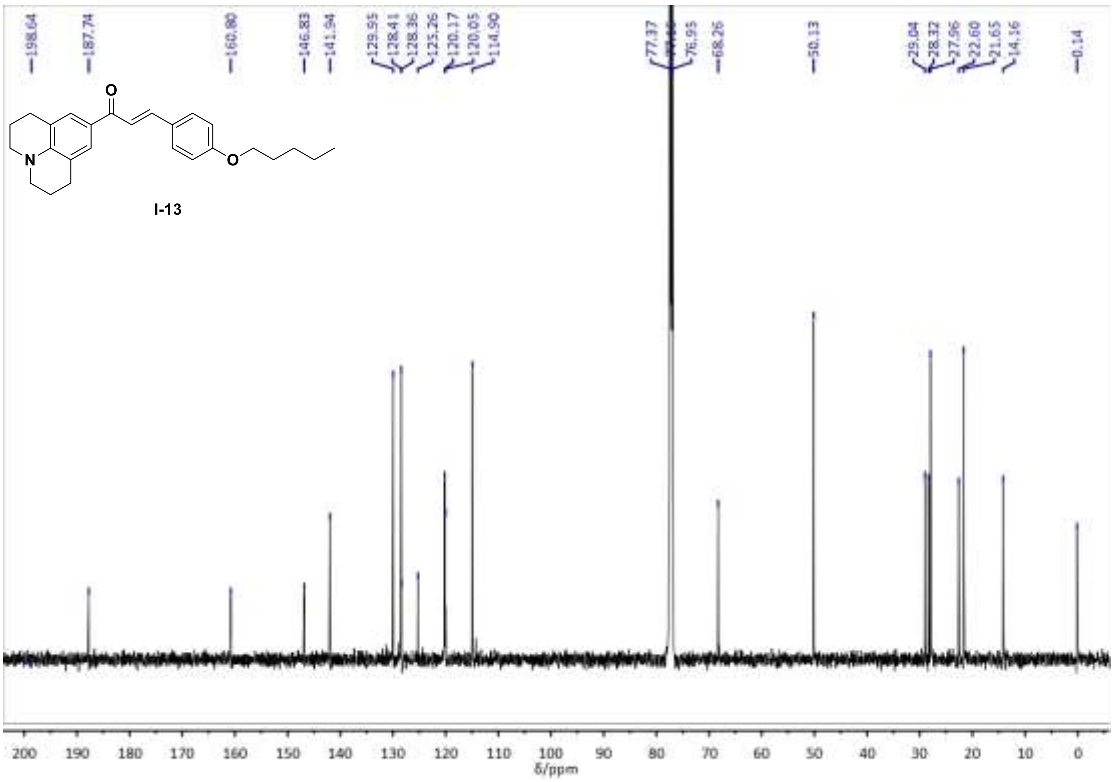

Figure S55 ^{1}H NMR (600 MHz, $CDCl_3$) and ^{13}C NMR (151 MHz, $CDCl_3$) spectra of I-13.

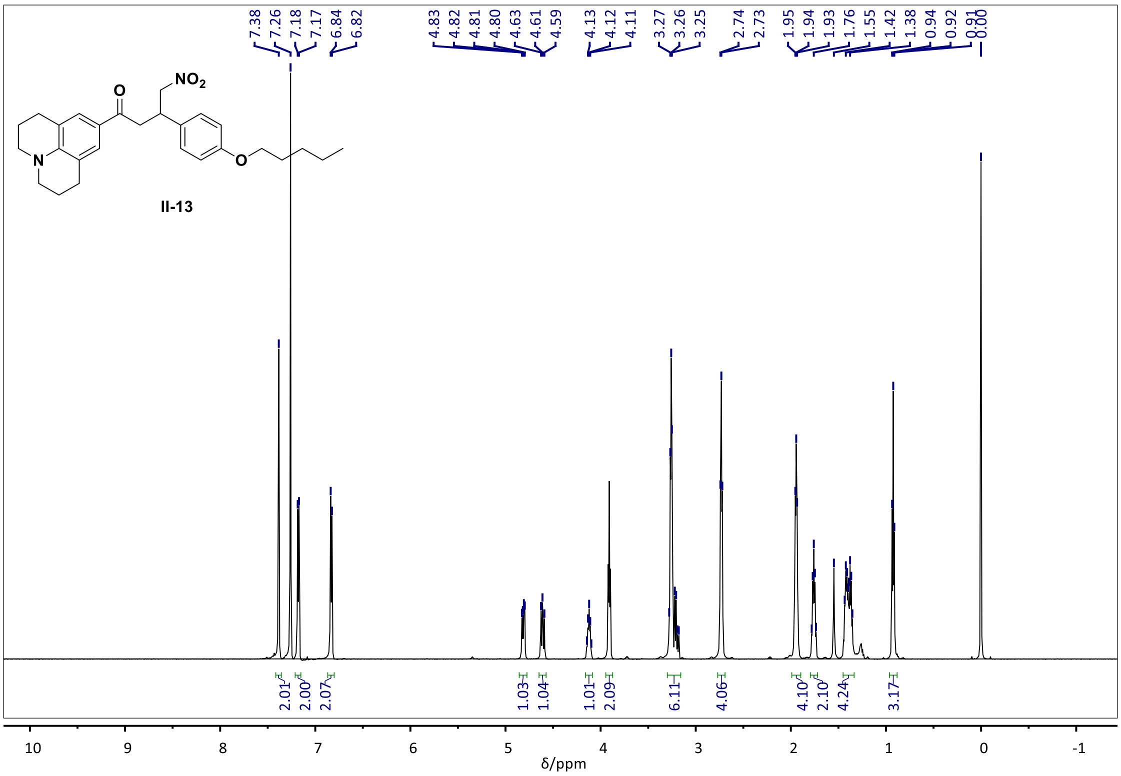

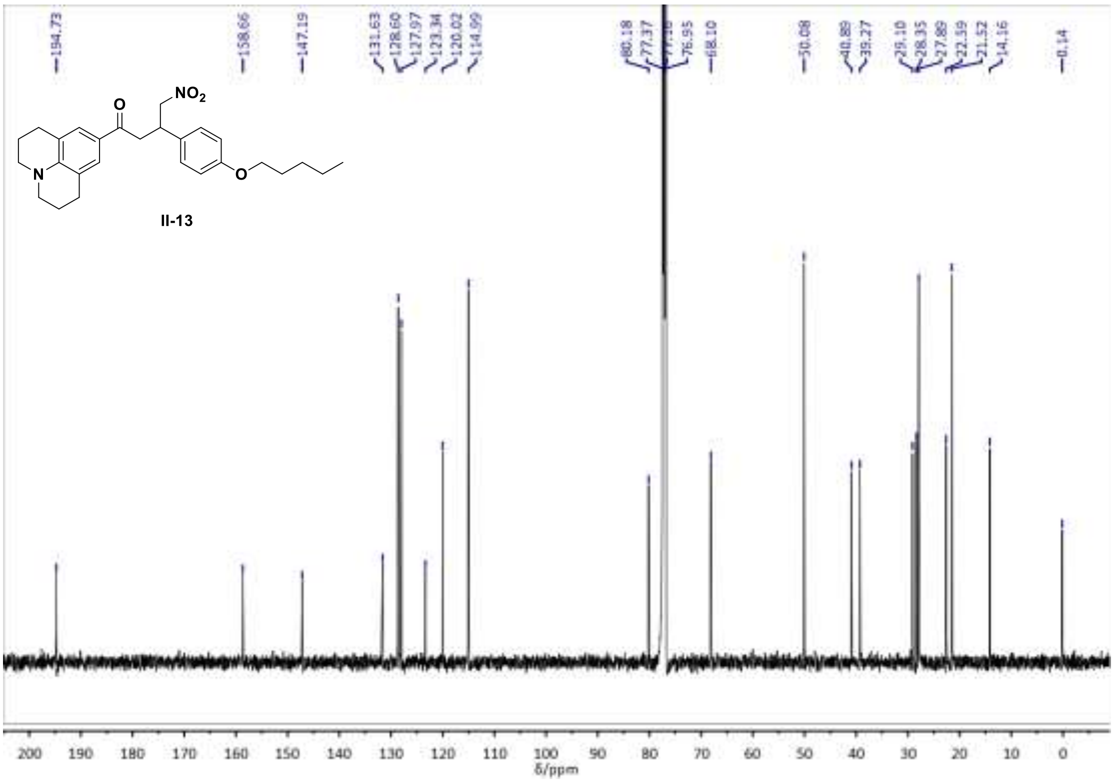

Figure S56 ^{1}H NMR (600 MHz, $CDCl_3$) and ^{13}C NMR (151 MHz, $CDCl_3$) spectra of II-13.

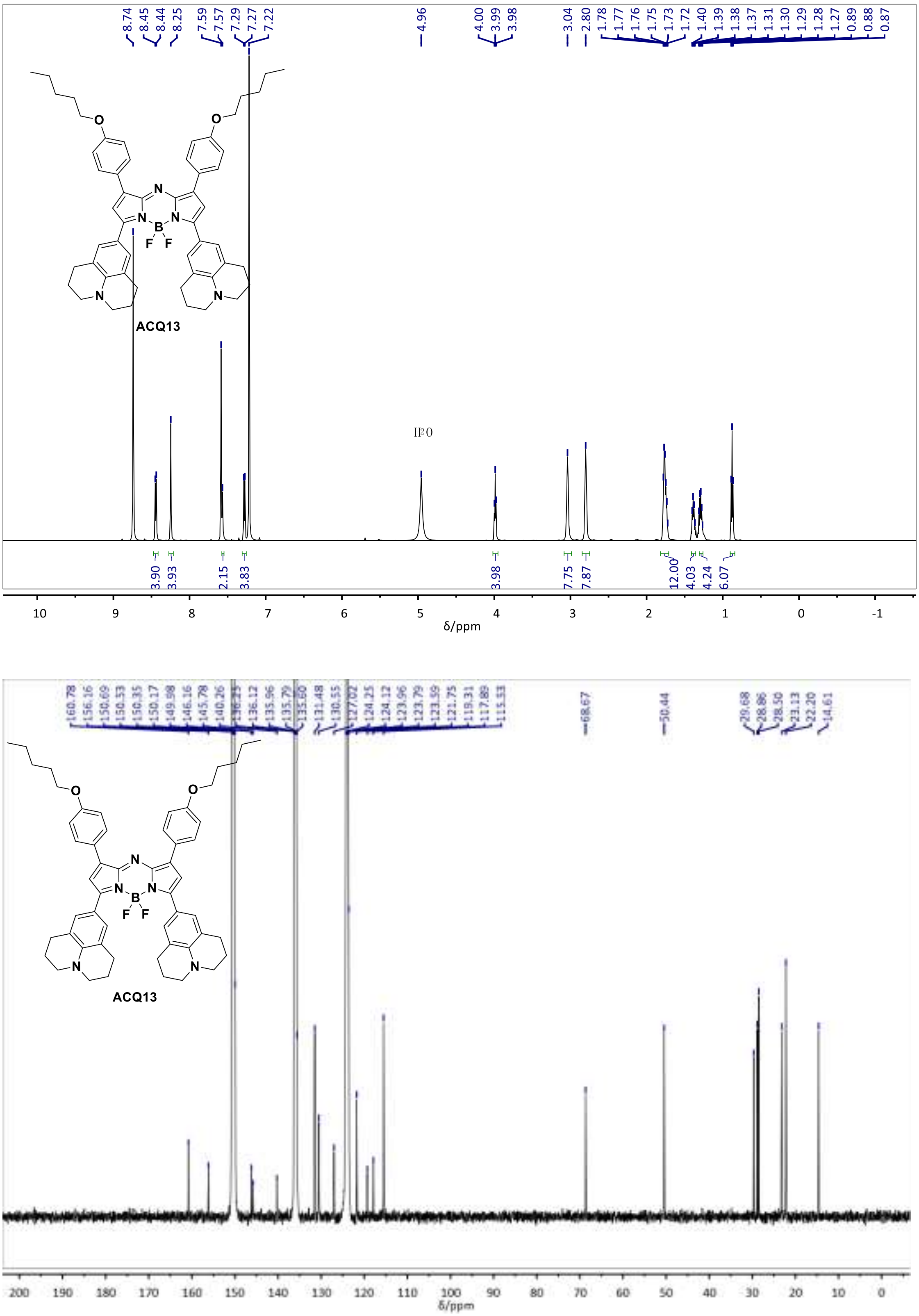

Figure S57 ^{1}H NMR (600 MHz, pyridine-d_5) and ^{13}C NMR (151 MHz, pyridine-d_5) spectra of ACQ13.

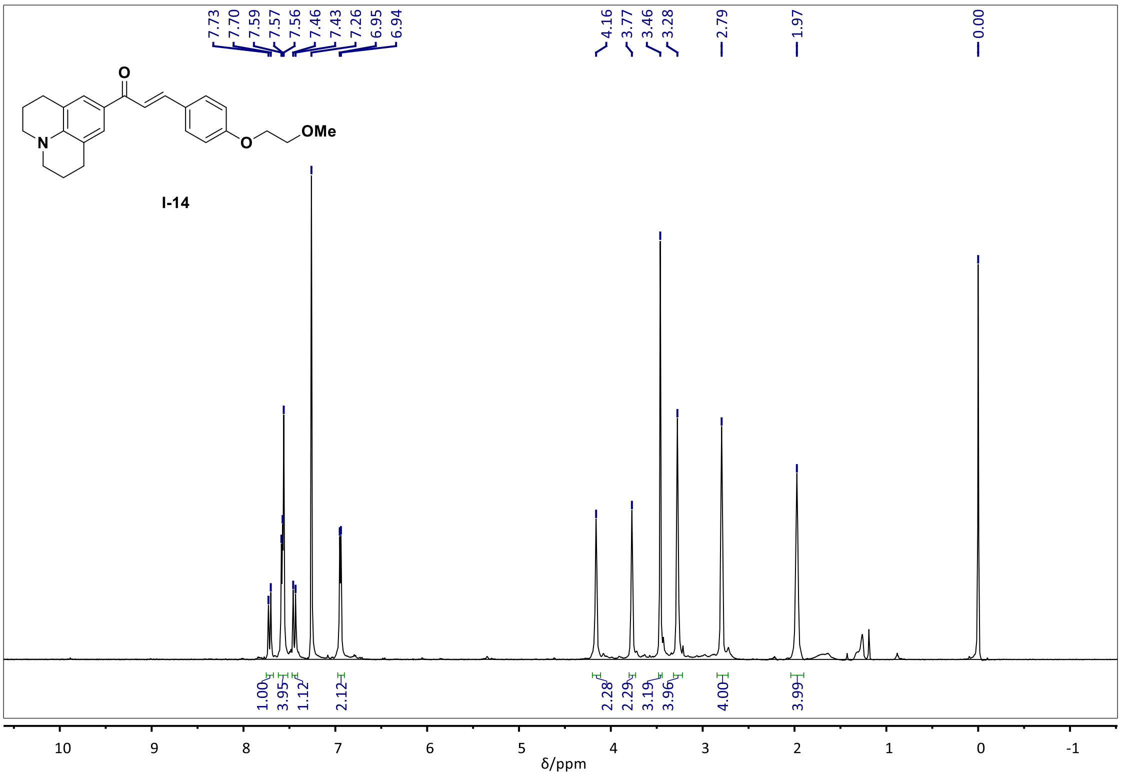

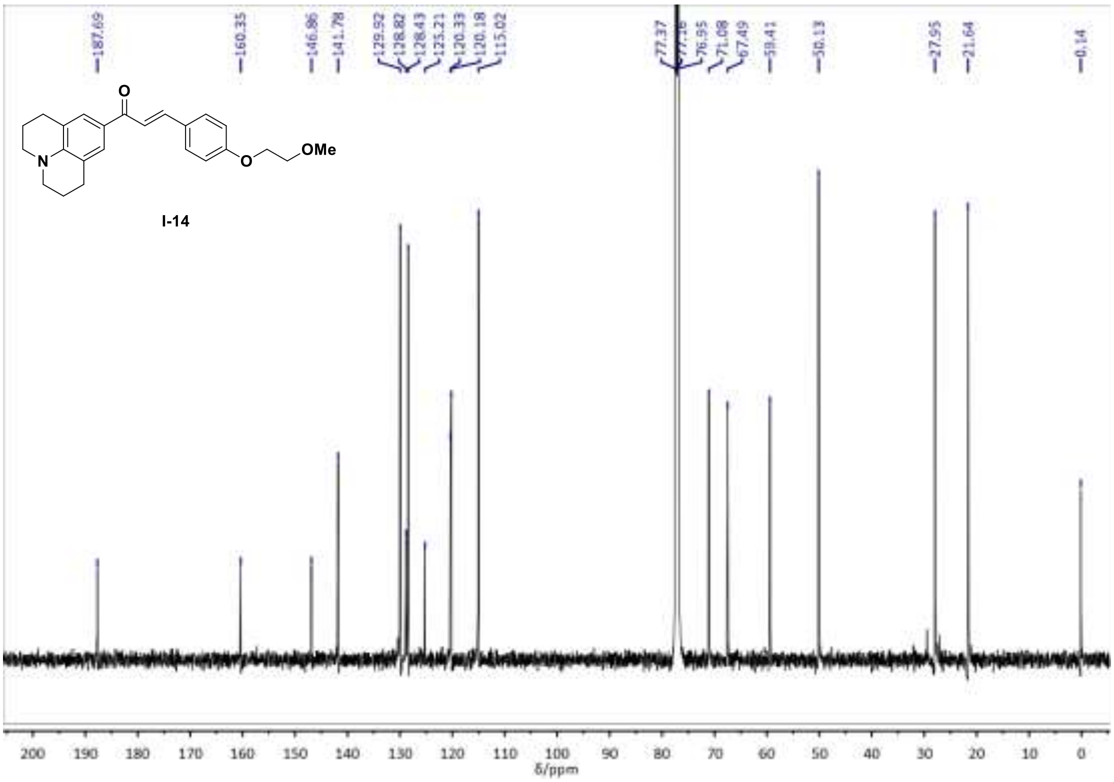

Figure S58 ^{1}H NMR (600 MHz, $CDCl_3$) and ^{13}C NMR (151 MHz, $CDCl_3$) spectra of I-14.

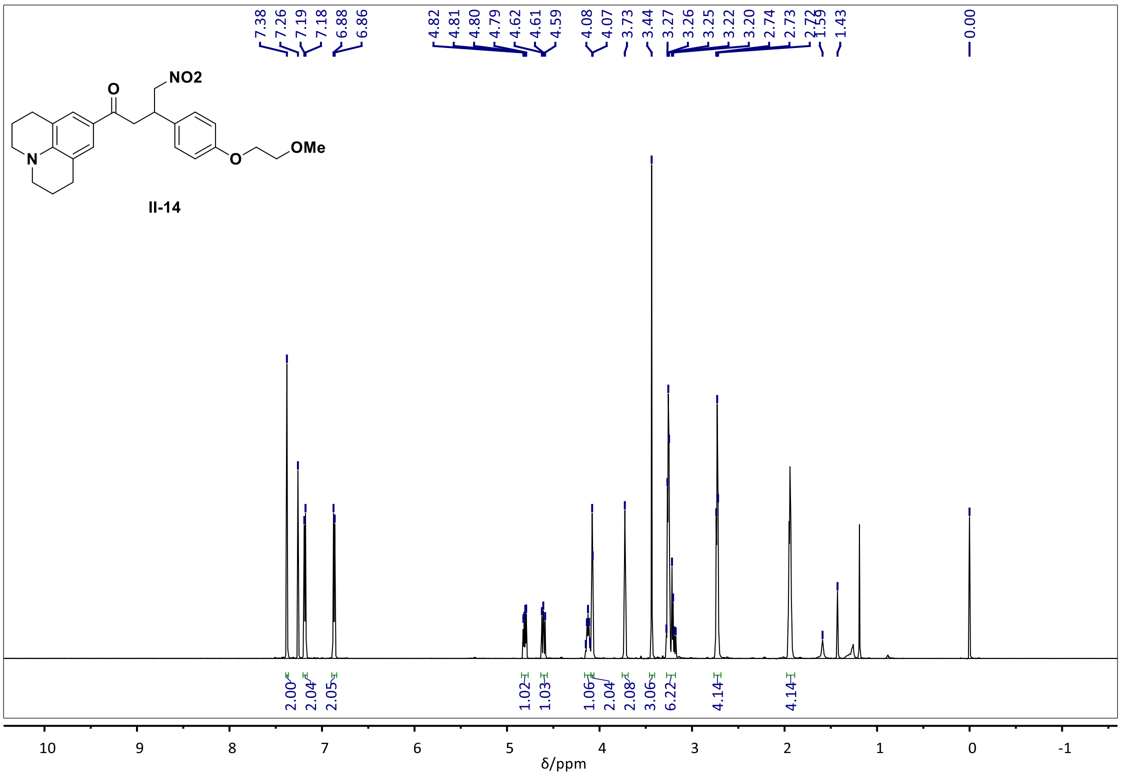

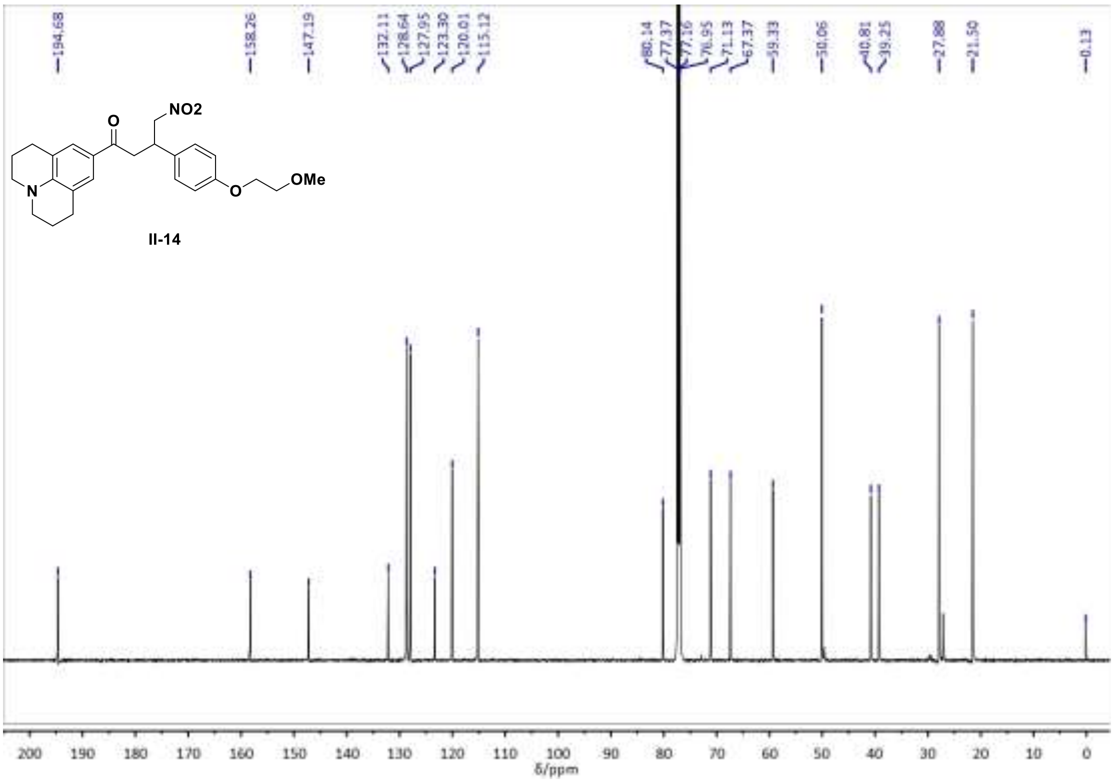

Figure S59 ^{1}H NMR (600 MHz, $CDCl_3$) and ^{13}C NMR (151 MHz, $CDCl_3$) spectra of II-14.

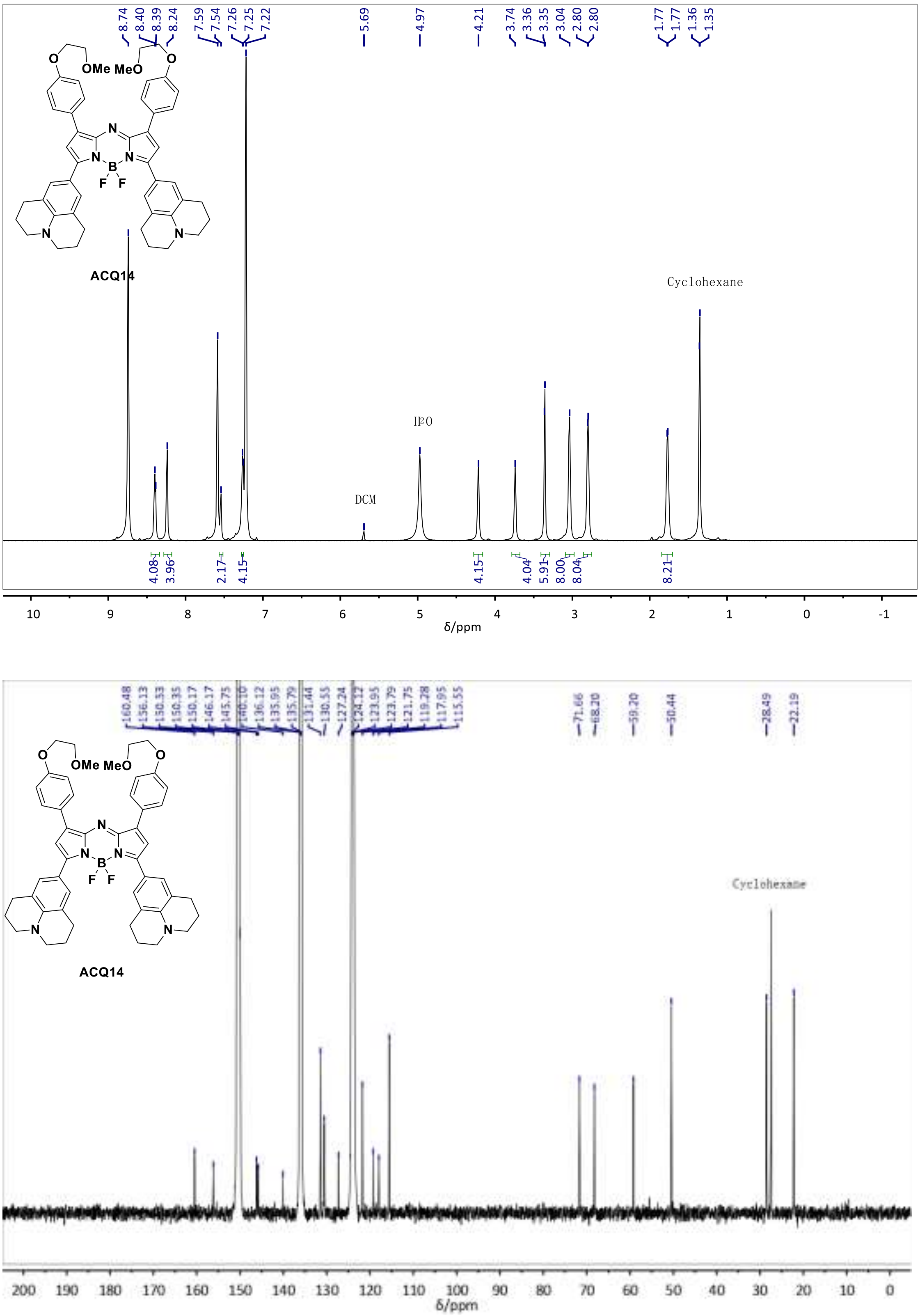

Figure S60 ^{1}H NMR (600 MHz, pyridine-d_5) and ^{13}C NMR (151 MHz, pyridine-d_5) spectra of ACQ14.

Reference

1. Zhao W, Carreira EM. Conformationally restricted aza-BODIPY: highly fluorescent, stable near-infrared absorbing dyes. *Chem Eur J* 2006;**12**:7254-63
